# Migration and the persistence of violence

**DOI:** 10.1073/pnas.2500535122

**Published:** 2025-11-24

**Authors:** Martin Vinæs Larsen, Gabriel S. Lenz, Anna Mikkelborg

**Affiliations:** ^a^Department of Political Science, Aarhus University, Aarhus 8000, Denmark; ^b^Travers Department of Political Science, University of California, Berkeley, CA 94720; ^c^Department of Political Science, Colorado State University, Fort Collins, CO 80523

**Keywords:** violence, migration, persistence

## Abstract

Using data on millions of internal US migrants, we document that historical homicide rates follow migrants around the United States. Individuals born in historically safe states remain safer wherever they go, while individuals born in historically dangerous states face a greater risk, including from police violence. This pattern holds across demographic characteristics such as age, gender, and marital status, across migrant groups with different average levels of education, income, and even when comparing migrants from different states who reside in the same county. To help understand why, we conducted a large national survey that oversampled internal White US migrants. The results suggest this persistence may reflect a sociocultural adaptation to dangerous settings. Residents and migrants from historically unsafe states—mainly former frontier states and the deep South—see the world as more dangerous, react more forcefully in aggressive scenarios, value toughness, distrust law enforcement, and say they rely on self and family in violent situations. These adaptations may have kept them safe in historically dangerous states, but may increase their vulnerability to harm in safer states.

## Significance

Why do some regions experience high rates of violence for generations, while others remain safe? This research uncovers a crucial insight: When individuals move from historically dangerous to safer areas, a significant part of their original risk of violent victimization travels with them. This suggests that the roots of violence are not solely determined by a person’s current circumstances but also by persistent characteristics—perhaps learned behaviors or cultural adaptations—that migrants carry from their original environments. Our findings, based on millions of US migrants, help explain how high homicide rates can stubbornly endure across different places and times.

Homicide is a leading cause of death for young men in the United States and many parts of the world, yet there is little consensus on its causes. A striking feature of homicide—and interpersonal violence more broadly—is its tendency to vary regionally, with this variation showing remarkable historical persistence (1–4). For example, Louisiana had about four times more homicides per capita than Massachusetts in the 1800s, and it still has about four times more today (5). In this paper, we show that this persistent regional variation in homicide follows people as they migrate around the United States: Those born in historically unsafe states remain at risk even after moving to safer states, while those born in safe states maintain a comparatively lower risk regardless of where they relocate.

To ensure that these results are not driven by selection—e.g., migrants from historically violent states self-selecting into more dangerous settings—we compare demographically similar White migrants who moved from different states but settled in the same state and county. With millions of migrants in our data, we are able to employ precise fixed effects for geographic and demographic categories. The results reveal that, even within the same county or same demographic group (e.g., 25- to 29-y-old males), those from more violent US states are considerably more likely to be victims of homicide than those from safer states. This heightened risk is evident even among those generally considered at low risk for lethal violence, such as married women, the elderly, and migrant groups with higher education and income. The consistency in the pattern of persistence suggests that it is not merely an artifact of particular types of migrants selecting into particular types of places. It is also not an artifact of gun ownership among migrants from historically unsafe states, as persistence rates are similar for gun and nongun homicides.

We focus on White internal US migrants because they are the only group with sufficient variation in historical homicide rates across states. Historically, Black Americans were concentrated in a small number of states, many of which had high homicide rates. Other groups were similarly concentrated until more recent decades.

Prior research on regional differences in violence suggests that an important reason for its persistence may lie in enduring cultural attitudes, particularly those associated with the “culture of honor” (6–13). This culture emphasizes defensive traits, especially a personal reputation for toughness and a readiness to respond to slights with force. It is a protective strategy against aggression (14, 15). Writing about Icelandic sagas, for instance, one scholar states, “honor, at root, still meant ‘Don’t tread on me’” (16). It has been linked to environments marked by historical violence and, in some cases, weak or mistrusted state institutions (1, 4, 17–21). However, the roots of such cultural traits are complex and contested—they may arise in response to weak state institutions, or alternatively, may themselves contribute to institutional weakness (22). What is clear is that such cultures have been observed in a wide range of historical settings characterized by violence and self-reliance. These include Corsican villages (23), Greek mountains (24), medieval Iceland (25), the samurai of Japan (26), and contemporary US inner cities (27).

To examine whether migrants carry such cultural traits with them, we fielded a large preregistered national survey, oversampling White interstate migrants. The survey reveals that migrants from historically violent regions report attitudes and behaviors consistent with descriptions of honor culture even after relocating. They are less trusting of criminal justice institutions like the police and tend to rely on themselves and their families for protection. They are also more likely to report a willingness to respond to slights with violence.

A culture-of-honor framework may also help explain several surprising aspects of the persistent homicide patterns we observe, such as why persistence appears across such a wide variety of groups, from married women to those over 75. This framework also helps explain why persistence occurs with homicide victimization, even when we might expect it to occur only with perpetration. In contexts where justice is handled privately, individuals often shift roles—meting out justice in one moment and being on the receiving end in another. “This private provision of security,” one scholar writes about the culture of honor, “creates a hair-trigger society…prone to unleash violent reprisals” (28). At the same time, we emphasize that identifying mechanisms is inherently difficult—other factors likely contribute to the persistence of homicide rates among migrants—and our results should be interpreted as suggestive of a culture-of-honor mechanism.

## Results

### Does Violence Persist among Interstate Migrants?

**Fig. 1.**
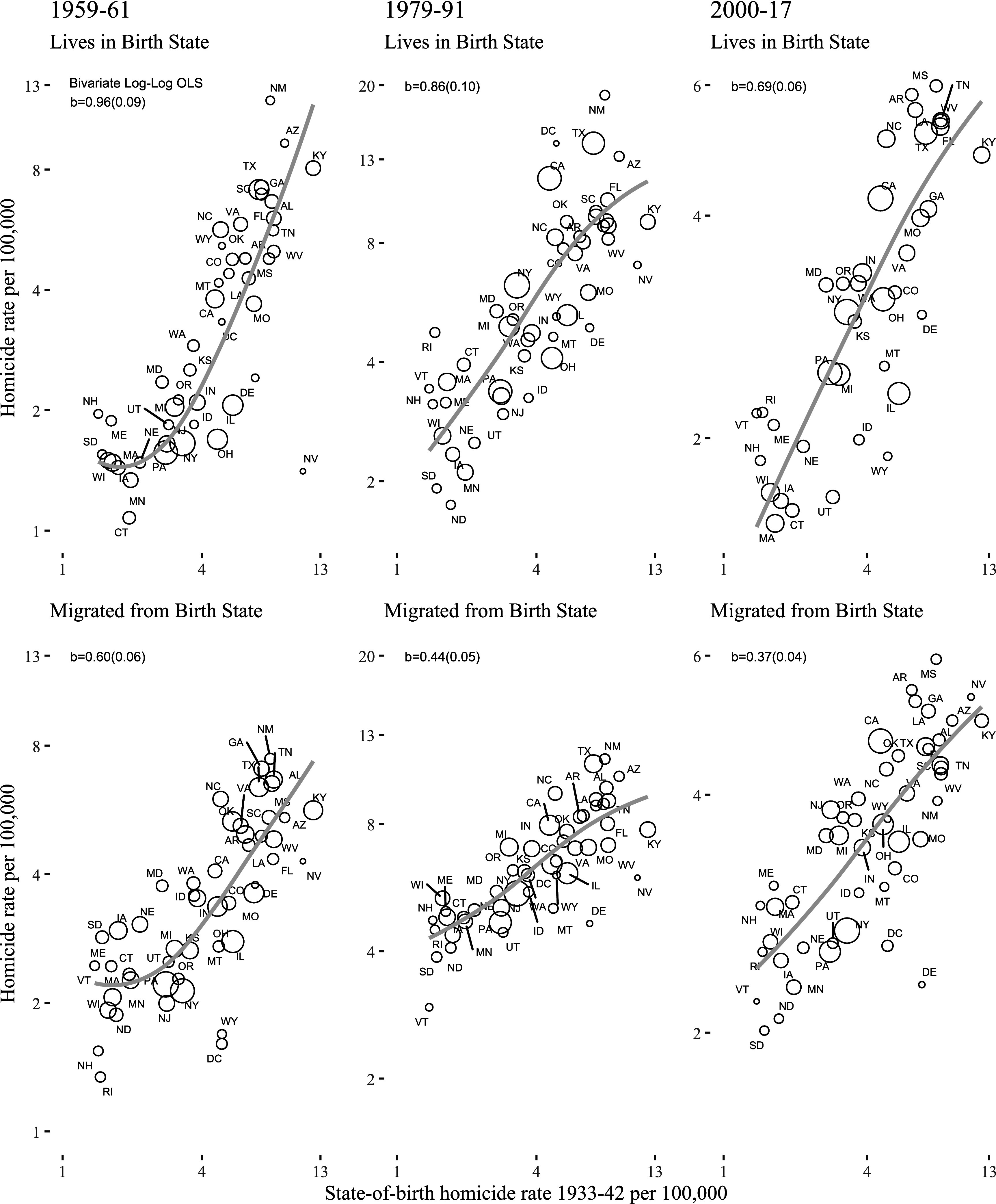
US Homicide Victimization Rate 1959–61, 1979–91, and 2000–17 by 1933–42 Historical State-of-Birth Homicide Victimization Rate for Whites Ages 15 to 59 by Interstate Migration Status. The *Top* panels show the scatterplots for nonmigrants while the corresponding *Bottom* panels show the scatterplots for migrants. For migrants, each point shows the homicide rate averaged across the US states those migrants ended up in. Loess lines are weighted and circles are sized by White population. Note that the y-axis scales vary across columns to make states visible. See *SI Appendix*, Table S3 for homicide rates for nonmigrants and migrants from each state in each era.

How much of the regional variation in the risk of violent victimization do migrants carry with them? Using data from US death certificates, Fig. 1 examines this question at the state level for all White internal migrants, plotting the nonmigrant (*Top*) and migrant (*Bottom*) homicide victimization rate by the birth state homicide rate from 1933–1942, the first decade for which we can measure it. Homicides are rare events, but aggregated to the state of birth, they reveal a clear pattern.

The figure’s top panels indicate strong persistence over time, showing that individuals born and residing in states with high homicide rates in the 1930s, such as Kentucky, continue to face the highest homicide risks in 1959–61, 1979–91, and 2000–17. The figure’s bottom panels show the key result of this paper: Much of that persistence carries over to migrants who leave their birth states and settle somewhere else in the United States. That is, the historical homicide rate in a migrant’s birth state strongly predicts their risk of homicide victimization, even after they relocate to another state. Here, each circle shows the homicide rate of migrants wherever they ended up. For example, the Kentucky data points show the average homicide rate for individuals born in Kentucky—the most violent state for Whites in the 1930s—but who migrated to some other US state. As can be seen from this figure, they retain much of their elevated risk of violent death after migrating, which they mainly did to the safer Midwest states. By contrast, individuals from say Wisconsin—one of the safest states for Whites in the 1930s—maintain a lower risk of violent death even after moving elsewhere.

Table 1 presents formal estimates of persistence by regressing the log of homicide rates (+1) on the log of 1933–42 homicide rates. We weight the estimates by the size of each migrant group. Due to space constraints, we only show the estimates for 1959–61 and 2000–17 and present the 1979–91 estimates in the *SI Appendix*, Tables S1 and S2. For descriptive statistics, see *SI Appendix*, Tables S3–S9. The first row of this table presents the bivariate linear regressions corresponding exactly with Fig. 1, with one data point for each state of birth. They reveal that a one-percent increase in the state-of-birth homicide rate corresponds with a 0.96 percent increase for nonmigrants and a 0.60 percent increase for migrants in 1959–61, which implies a (0.60/0.96*100=) 62% migrant persistence rate. The corresponding coefficients for 2000–17 are 0.69 and 0.37, a 54% persistence rate (for 1979–91, they are 0.86 and 0.44, which implies about a 50% persistence rate).

**Table 1.** Persistence of homicide victimization rates among White internal US migrants compared to nonmigrants from the 1930s to 1959–61 and 2000–17

Model	1959–61					2000–17				
	Non-Mig.		Migrants		Persistence	Non-Mig.		Migrants		Persistence
	Coef.	SE	Coef.	SE	%	Coef.	SE	Coef.	SE	%
*Analysis at the birth-state level (n = 49 states of birth)*										
1. Bivariate reg. on data in Fig. 1	0.96*	0.09	0.60*	0.06	62	0.69*	0.06	0.37*	0.04	54
Analysis at the birth-state * residence-state * age-group level with controls for state-residence * age-group FE for migrants and age-group FE for non-migrants										
2. Baseline reg. at this level	0.94*	0.09	0.47*	0.04	50	0.67*	0.06	0.21*	0.04	31
3. Females	0.42*	0.08	0.29*	0.05	69	0.46*	0.05	0.14*	0.04	30
4. Males	1.23*	0.11	0.53*	0.05	43	0.80*	0.07	0.25*	0.05	31
5. Married females	0.41*	0.09	0.30*	0.04	73	0.38*	0.04	0.08*	0.03	21
6. Married males	1.32*	0.10	0.50*	0.05	38	0.68*	0.08	0.21*	0.04	31
7. Unmarried females	0.34*	0.09	0.10	0.06	30	0.51*	0.08	0.19*	0.06	37
8. Unmarried males	1.09*	0.15	0.36*	0.08	33	0.83*	0.07	0.26*	0.07	31
9. Age less than 15	0.08	0.05	0.01	0.03	16	0.25*	0.04	0.12*	0.03	48
10. Age 15-29	0.86*	0.10	0.49*	0.05	57	0.62*	0.06	0.21*	0.05	34
11. Age 30-44	1.08*	0.10	0.57*	0.05	53	0.73*	0.07	0.27*	0.05	37
12. Age 45-59	0.91*	0.09	0.35*	0.06	39	0.66*	0.06	0.17*	0.04	26
13. Age 60-74	0.68*	0.07	0.27*	0.06	40	0.58*	0.05	0.15*	0.03	26
14. Age 75 and up	0.62*	0.15	0.04	0.06	6	0.48*	0.06	0.12*	0.03	25
*Analysis at the county by state-of-birth level controlling for age, age squared, female percent, and log of group size, and county FEs for migrants*										
15. Baseline reg. at the county level	0.80*	0.17	0.39*	0.05	49	0.61*	0.09	0.18*	0.03	30
16. Above the median county population	0.91*	0.21	0.43*	0.06	47	0.55*	0.12	0.17*	0.03	31
17. Below the median county population	0.56*	0.14	0.19*	0.05	33	0.51*	0.14	0.20*	0.04	39

Note: This table shows estimates of the effect of the log of 1933–42 state-of-birth White homicide rate on the log +1 of homicide rates in 1959–61 and 2000–17. Each set of coefficients and SE is from a separate regression. Except for the first row, SE are clustered by state of birth. Data are weighted by population. In row 1, the Ns are 49. In row 2, the Ns for migrants are 49 states of birth (including DC, but excluding AK and HI) within each of 51 states of residence (including DC) separately for nine 5-y age groups (ages 15 to 59), so (49 times (51 − 1) times 9=) 22,050 groups, though we have missing population data for small migrant groups, especially in small states—1,810 groups in 1959–61 and 15 in 2000–17. It is 51 − 1 in this calculation since each of the 49 states of birth can pair with 51 − 1 possible migrant groups, not 51, because those born in their state of residence cannot be migrants in their own state. For counties in 1959–61, we use all data points for which the 5 percent census files in 1980 and 1990 contain respondents. In 1959–61, this yields an N of 383 nonmigrant counties and 15,936 migrant groups in US counties. In 2000–17, these Ns are 454 and 21,866. For nonmigrants, the models do not include state of residents fixed effects or county fixed effects because these are colinear with the historical state-of-birth homicide rate. Ages 15 to 59 except rows 9 to 14. *P < 0.05 (two-sided, as are all tests).

See *SI Appendix*, Tables S12 and S13, for the full models.

This persistence among migrants could simply reflect self-selection. Migrants from dangerous states may disproportionately choose to settle in higher-risk areas, or perhaps younger individuals or unmarried males—groups at higher risk of violence—are more likely to migrate from violent states. To address these and other potential sources of selection bias, we estimate linear regression models that incorporate a range of geographic and demographic fixed effects and analyze various subgroups. These models allow us to observe persistence patterns within each state or county of residence and within each demographic group. For example, by including county fixed effects, we ensure that comparisons are made between similar contexts—such as Texans living in Los Angeles County compared to New Yorkers living in Los Angeles County—rather than across disparate locations, like Texans in Los Angeles County and New Yorkers in Suffolk County, Massachusetts.

We first address selection on age and into specific destinations by including fixed effects for state of residence and for 5-y age groups. To include these, the models in rows 2 to 14 of Table 1 disaggregate the data to the state-of-residence, state-of-birth, and 5-y age group level and cluster the standard errors on state of birth. The units of analysis for migrants are specific groups, such as 25- to 29-y-old White individuals born in Kentucky and living in Illinois. For nonmigrants, the units are comparable groups—e.g., 25- to 29-y-old White individuals born in Kentucky and still living there. For migrants, the models in these rows include state-of-residence times age-group fixed effects, so all comparisons are within each state-of-residence-age group. The estimates in row 2 show that including these fixed effects decreases estimated persistence among migrants from 0.60 to 0.47 in 1959–61 and from 0.37 to 0.21 in 2000–17, implying that some of the persistence pattern may be attributable to selection on these variables, but much remains. Consistent with selection not fully accounting for the findings, we find that people from historically violent places select into more violent states at lower rates than might be expected (*SI Appendix*, Figs. S1 and S2). In rows 3 to 14, we take exactly the same model, but break the analysis up and reestimate it separately among females, males, married females, married males, unmarried females, unmarried males, individuals under 15 and then in 15-y age groups up to 75+. The estimates are understandably noisy, but they suggest that the persistence pattern holds up across these categories from unmarried females to the elderly. Strikingly, even migrants 75 y and older in 2000–17 and 1979–91 (see *SI Appendix*, Table S1, for the latter) are more at risk of homicide when they originate from more dangerous states. (This pattern is imprecisely estimated in 1959–61, because there were not as many Americans above the age of 75.)

Even after accounting for these forms of selection, the persistence pattern remains large. Based on the row 2 estimate for 1959–61, the increase in homicide rates for White migrants born in the most violent states exceeded the entire decline in rates associated with aging from 20 to 80 by 140%. It also amounted to 50% of the total gap between Black and White nonmigrants (for details on these calculations, see *SI Appendix*, section S5).

To address potential selection into dangerous counties, rows 15 to 17 disaggregate the data to the county-of-residence level and include county-fixed effects, with standard errors clustered at the state-of-birth level. We do not disaggregate by age as well due to the limited number of migrants from each state of birth in most counties. Instead, we control for age, age squared, percent female, and the log of group size. Row 15 shows that persistence patterns remain largely consistent when we examine them within each county. Rows 16 and 17 indicate that this persistence occurs in both large and small counties by population.

Overall, these findings suggest that the persistence of violence among migrants is broad-based and not attributable to the age or gender of those migrating, or to where they settle. Whatever drives this persistence appears to operate across demographic groups. Notably, the patterns by age and gender are strikingly similar for migrants and nonmigrants, implying that comparable forces shape outcomes in both populations. These parallels are difficult to reconcile with explanations based primarily on selection.

Another possible source of selection is that migrants from historically violent states might choose economic activities that put them at greater risk of homicide, or settle in places where they are especially marginalized. While death certificates do not contain socioeconomic information, the census does. We therefore link group-level measures of education and income from the census to our migrant groups. For example, we calculate the average years of schooling among Louisiana-born migrants living in Los Angeles County. Using these averages, we classify migrant groups into quartiles of education and income. We then reestimate the persistence regressions within each quartile. Table 2 shows results for 1959–61 and 2000–17, with state-level results in the top panel and county-level results in the bottom panel. Importantly, nothing in our data is changing in these analyses—the dependent variable, independent variables, and models are the same—we are simply reestimating by migrant group-level characteristics as opposed to, say, reestimating by individual or geographical characteristics. We find evidence of persistence across almost all quartiles of migrant group education and income, though the strength of persistence is somewhat weaker among the most educated and highest-income groups. We also classify migrant groups by their relative position compared to nonmigrants in the receiving area (i.e., quartiles of education and income inequality relative to locals). Persistence is again evident, including among groups who are better educated and more affluent than their new neighbors. Because these estimates rely on migrant group averages, not individual data, we must be wary of drawing inferences about individuals. Nonetheless, the findings suggest that persistence may not be confined to a single disadvantaged segment of the migrant population, but instead appears across the economic and educational spectrum. Consistent with this, migrants from historically violent states are on average better educated and better off than their new neighbors (*SI Appendix*, Figs. S5–S8).

**Table 2.** Robustness in the persistence of homicide victimization rates among White internal US migrants from the 1930s to 1959–61 and 2000–17

Model	1959–61				2000–17			
	Coef.	SE	R2	N	Coef.	SE	R2	N
*Analysis at the birth-state level (n = 49 states of birth)*								
1. Bivariate regression estimates on data in Fig. 1	0.60*	0.06	0.64	49	0.37*	0.04	0.59	49
Analysis at the birth-state * residence-state * age-group level with controls for state-residence * age-group FE, female %, and log of group size								
2. Baseline regression estimates at this level of disaggregation	0.43*	0.05	0.31	20,240	0.23*	0.03	0.37	22,035
3. 1st quartile years in school among migrant groups	0.35*	0.17	0.50	1,460	0.20*	0.05	0.43	4,286
4. 2nd quartile years in school	0.14*	0.07	0.39	3,951	0.21*	0.04	0.40	4,286
5. 3rd quartile years in school	0.22*	0.06	0.32	5,122	0.23*	0.04	0.35	4,285
6. 4th quartile years in school	0.09*	0.04	0.32	5,065	0.04	0.04	0.34	4,285
7. 1st quartile HH income among migrant groups	0.24*	0.10	0.42	2,581	0.23*	0.04	0.37	5,506
8. 2nd quartile HH income	0.39*	0.07	0.44	5,233	0.24*	0.05	0.39	5,505
9. 3rd quartile HH income	0.38*	0.07	0.36	6,228	0.24*	0.03	0.39	5,506
10. 4th quartile HH income	0.15*	0.06	0.33	6,198	0.11*	0.04	0.35	5,505
11. 1st quartile HH income inequality between migrant group and nonmigrants (migrants best-off)	0.07	0.05	0.20	5,022	0.16*	0.05	0.45	5,312
12. 2nd quartile HH income inequality between migrant group and nonmigrants	0.22*	0.06	0.39	4,872	0.16*	0.03	0.41	5,280
13. 3rd quartile HH income inequality between migrant group and nonmigrants	0.20*	0.06	0.39	4,817	0.16*	0.04	0.43	5,280
14. 4th quartile HH income inequality between migrant group and nonmigrants (migrants worst-off)	0.42*	0.07	0.36	4,722	0.28*	0.04	0.44	5,282
15. 1st quartile education inequality between migrant group and nonmigrants (migrants most-educated)	0.07	0.08	0.36	4,940	0.16*	0.05	0.47	5,312
16. 2nd quartile education inequality between migrant group and nonmigrants	0.06	0.06	0.39	4,787	0.09*	0.04	0.36	5,278
17. 3rd quartile education inequality between migrant group and nonmigrants	0.37*	0.05	0.45	4,786	0.18*	0.04	0.44	5,282
18. 4th quartile education inequality between migrant group and nonmigrants (migrants least-educated)	0.27*	0.06	0.48	4,920	0.24*	0.04	0.43	5,282
19. Above median migrant population	0.46*	0.05	0.30	9,601	0.24*	0.03	0.38	11,016
20. Below median migrant population	0.03	0.02	0.10	10,639	0.09*	0.04	0.13	11,019
21. Only migrants who crossed census regions	0.37*	0.07	0.34	15,216	0.21*	0.04	0.33	16,563
22. Northeastern residence	0.22*	0.07	0.25	3,879	-0.03	0.05	0.39	3,884
23. Midwestern residence	0.57*	0.08	0.30	4,370	0.32*	0.03	0.28	5,177
24. Southern residence	0.39*	0.10	0.30	5,610	0.27*	0.05	0.31	7,341
25. Western residence	0.33*	0.09	0.31	6,381	0.23*	0.05	0.29	5,633
*Analysis at the county by state-of-birth level with controls for county fixed effects, age, age squared, female %, and log of group size*								
26. Baseline regression estimates at the county level	0.39*	0.05	0.38	15,936	0.18*	0.03	0.43	20,885
27. 1st quartile years in school among migrant groups	0.41*	0.11	0.50	3,992	0.20*	0.07	0.47	5,168
28. 2nd quartile years in school	0.19*	0.06	0.44	3,994	0.18*	0.04	0.46	5,168
29. 3rd quartile years in school	0.17*	0.08	0.45	3,967	0.13*	0.03	0.51	5,168
30. 4th quartile years in school	0.09	0.07	0.30	3,983	0.08*	0.04	0.50	5,167
31. 1st quartile education inequality between migrants and nonmigrants	0.16*	0.06	0.33	4,444	0.15*	0.04	0.48	5,185
32. 2nd quartile education inequality between migrants and nonmigrants	0.04	0.06	0.34	3,985	0.11*	0.04	0.48	5,185
33. 3rd quartile education inequality between migrants and nonmigrants	0.15*	0.06	0.48	3,799	0.14*	0.03	0.53	5,185
34. 4th quartile education inequality between migrants and nonmigrants	0.48*	0.08	0.46	3,534	0.23*	0.06	0.48	5,185
35. 1st quartile HH income among migrant groups	0.12	0.11	0.46	3,984	0.19*	0.06	0.42	5,222
36. 2nd quartile HH income	0.31*	0.07	0.47	3,984	0.24*	0.04	0.45	5,230
37. 3rd quartile HH income	0.34*	0.08	0.43	3,984	0.10*	0.04	0.48	5,212
38. 4th quartile HH income	0.19*	0.07	0.47	3,984	0.10*	0.03	0.51	5,221
39. 1st quartile HH income inequality between migrants and nonmigrants	0.09	0.10	0.33	4,389	0.12*	0.04	0.51	5,184
40. 2nd quartile HH income inequality between migrants and nonmigrants	0.21*	0.08	0.41	3,860	0.13*	0.04	0.47	5,186
41. 3rd quartile HH income inequality between migrants and nonmigrants	0.23*	0.06	0.46	3,802	0.19*	0.03	0.50	5,185
42. 4th quartile HH income inequality between migrants and nonmigrants	0.12	0.12	0.52	3,711	0.18*	0.05	0.49	5,185
43. Above the median migrant population	0.40*	0.05	0.38	7,863	0.17*	0.03	0.47	10,434
44. Below the median migrant population	0.05*	0.01	0.09	8,073	0.06*	0.03	0.17	10,451

Note: This table shows estimates of the effect of the log of 1933–42 state-of-birth White homicide rate on the log +1 of homicide rates in 1959–61 and 2000–17. The coefficients shown are for the state-of-birth homicide rate for Whites in 1933–42. Each set of coefficients and SE is from a separate regression. Except for the baseline estimate rows, SE are clustered by state of birth. *SI Appendix* shows the estimates for 1979–91 and for homicide count models. See note to previous table for more details. Subgroup Ns do not always add up to the total because of missing census data for small migrant groups. *P < 0.05.

See *SI Appendix*, Tables S14–S17, for the full models.

How much of this persistence arises from migrant groups congregating in large enclaves? Answering this question is beyond the scope of this paper, but we can conduct a preliminary analysis. Table 2 presents estimates for migrant groups above and below the median migrant group size (at the state level and at the county level). The estimates reveal little persistence among below-the-median size groups, but strong persistence in above-the-median size groups. In additional analyses, we find that persistence appears to increase with group size (not plateauing as it should if larger group sizes simply reduce noise, see *SI Appendix*, Fig. S9). However, the estimates are imprecise, and therefore at best suggestive. These signs that larger groups contribute to persistence, though, are consistent with other findings, which show a positive relationship between enclave size and rates of main language acquisition (29–32).

Finally, Table 2 shows that persistence generally holds up across US regions. It implies that people from safe states remain disproportionately safe even when they migrate to unsafe regions like the South. For example, in 1959–61, the homicide rate for Southern-born migrants living in the South was 5.9 per 100,000 (about the same as that experienced by nonmigrants in the South). By contrast, Northeast-born migrants in the South were much safer with a homicide rate of 2.8. They were not quite as safe as they would have been had they stayed in the Northeast, which had a homicide rate of 1.6, but they were still much safer than Southerners in the South. This pattern holds for 1979–91 and 2000–17. This cannot be attributed to age, sex, or their locations of residence since Table 2 conditions on these and, as already noted, persistence exists among better educated, higher income migrant groups and the elderly.

Another explanation for this persistence is gun ownership. Those from historically violent states may own guns at higher rates and guns can turn disputes into homicides. The death certificate data include information on the instrument, enabling us to isolate gun-related violence. We find considerable persistence for both gun homicides and for nongun homicides (*SI Appendix*, Fig. S10), suggesting that variation in the availability of guns cannot account for our results.

Although we explore the culture of honor explanation in more detail in the next section, one aspect of the results warrants brief comment here—namely, that we find persistence for women. Cultures of honor are often understood as intensely male, so persistence among females may seem at odds with this account. However, real or perceived infidelity by wives or girlfriends is frequently viewed as a serious insult to male partners, prompting violent retaliation as a means of restoring male reputations as not someone to be trifled with (33–35). Women may also help sustain these norms by internalizing reputational logics and discouraging outside intervention. At the same time, while we detect some persistence among female migrants, the elevated risk of violence is primarily found among male migrants (*SI Appendix*, Figs. S3 and S4).

Another important aspect of the results is that the relationship between historical homicide rates and migrant group homicides weakens over time, as reflected in the smaller estimates for later cohorts in Tables 1 and 2. This attenuation likely results from a combination of factors. First, the nationwide decline in homicide and improvements in policing since the early 1990s may have lowered overall violence and narrowed intergroup differences. Second, later migrant cohorts may have experienced greater geographic and social integration, facilitating the assimilation of behavioral norms. Third, selective return migration may have gradually reduced the distinctiveness of those who remained outside historically violent states.

In the analyses above, we examine persistence based on 1933–42 White homicide rates because these are the first 10 y for which homicide rates are available from the death registry for all states, but our findings also do not depend on this decision. In fact, they tend to become stronger if we use later years (*SI Appendix*, Figs. S11 and S12).

The analysis above compares migrants from one state to migrants from another state who end up in the same state or county. We think comparing migrants to other migrants is the stronger design, but we find similar results when we compare migrants to locals. Indeed, in states that disproportionately received migrants from less-safe states (*SI Appendix*, Fig. S13, for a visualization), we find that these migrants experience disproportionately high rates of homicide. For example, 1,385,640 Kentuckians had migrated to Ohio and Indiana by 1959–61 and they died violently at almost 3 times the rate of locals (5.3 vs. 1.8 per 100,000).

To help us understand why historical homicide rates are persistent, we also explored robustness to other historical variables, including historical state income per capita, unemployment, agricultural share, and non-White share, in migrant-only regressions (*SI Appendix*, Tables S10 and S11). Per capita income and agricultural share, though not unemployment, initially predict migrant homicide rates in 1959–61, but their predictive power diminishes by 1979–91 and 2000–17. Although the predictiveness of non-White migrant share also declines over time in these models, it remains significantly predictive through 2000–17. Migrants from states with high non-White migrant shares disproportionately originate from the Deep South—the country’s historically most extractive region, characterized by weak state institutions and distinctive conditions that could leave migrants more vulnerable to violence. Importantly, these historical variables are inherently interrelated and vary substantially in measurement quality, influencing how strongly regression analyses will favor one predictor over another and limiting our ability to draw strong inferences. Overall, this analysis highlights the surprising ease of predicting migrants’ vulnerability to violent victimization based on their states of origin, which we see as the paper’s main contribution. However, it also underscores the difficulty of isolating the mechanism behind this persistence. Thus, while we offer an Occam-style interpretation—that historical homicide rates themselves are intuitively most related to later homicide rates—we readily acknowledge the uncertainty.

Finally, we find similar patterns with police-involved homicides, which are partially observable in death certificate data—estimated to capture 50 to 60% of such incidents (36, 37). Individuals from historically violent states experience higher rates of police violence—even after migrating to new states (*SI Appendix*, Fig. S14).

### Survey.

If the persistence of violence among migrants from unsafe states stems from adherence to norms and behaviors rooted in a culture of honor, we would expect them to have a fundamentally different approach to public safety and criminal justice than those migrating from safer states. To study this prediction, we conducted a national survey of non-Hispanic White migrants and nonmigrants, oversampling migrants so that they composed about half of the sample. We then examine the relationship between the historical homicide rate in the state where these migrants grew up and their beliefs, experiences, attitudes, and values related to public safety. To reduce measurement error, we measured many of these with multiple items (*SI Appendix*, sections S6–S9 for details, preregistration, question wording, and construct reliability; Cronbach’s alphas were generally 0.7 or higher). Specifically, we expect that individuals from historically high-homicide states will exhibit the following patterns:

First, we expect them to have grown up witnessing more violence (three-item scale) and to hold a heightened perception of personal risk, believing that being mugged, violently attacked, or having their home invaded is more likely (three-item scale). This heightened sense of vulnerability is tied to a broader worldview that sees the world as fundamentally dangerous and unpredictable (two-item scale).

Second, they are likely to display less trust in institutions. This includes having lower confidence in local governments (two items) and viewing the police as less effective in ensuring safety (three-item scale). Instead, they are more inclined to rely on family rather than law and courts when someone in their family is victimized (two-item scale). Additionally, they should have lower trust in other people (two items).

Third, we expect a stronger inclination toward protective behaviors and adherence to an honor-based ideology. They are more likely to own guns for self-defense (two items) and to embrace a belief system where a “real man” never backs down from a fight, is willing to use physical aggression when provoked, and “doesn’t take any crap from anybody” [Honor Ideology in Manhood, (38), three-item scale]. This honor ideology extends to how they view themselves, as they are more likely to describe themselves as hotheaded or having uncontrolled tempers—characteristics that may serve as protective reputations in environments where violence and theft are common [(39), three-item scale].

Finally, in scenarios involving direct threats or provocation—such as having a drink poured on their head in a bar, being scratched repeatedly on a school bus, or being shoved at a movie theater—these individuals are expected to respond more aggressively than others (three-item scale). They are also more likely to perceive that their friends and typical individuals from their community (matched to the gender of the person in the scenario) would respond aggressively in similar situations (three items each). Walking away from such confrontations, in their view, would not only make them appear weak but also diminish their sense of being a “real man” (two items each).

**Fig. 2.**
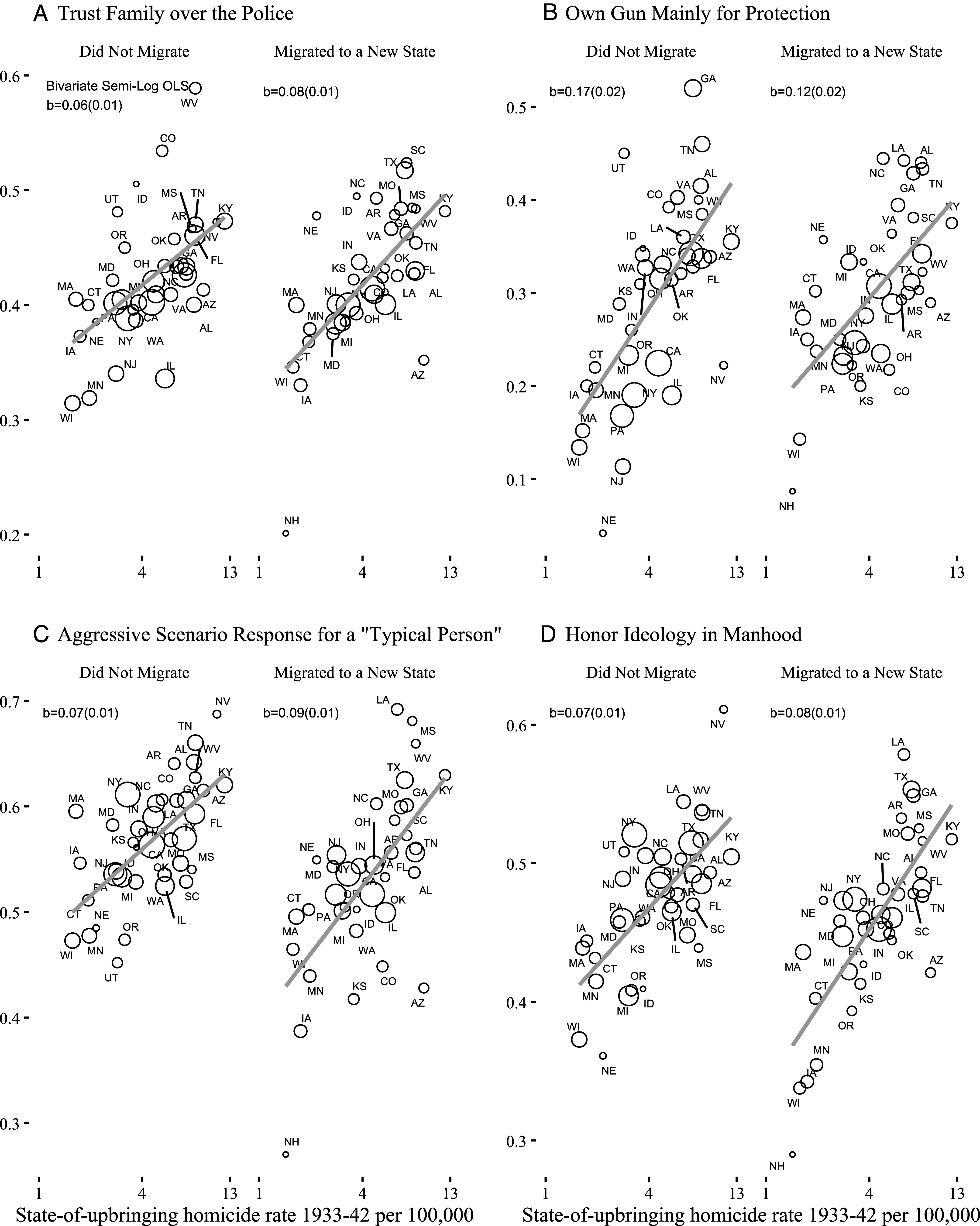
Selected survey measures (*A*–*D*) by historical state-of-upbringing homicide victimization rate for Whites by migration status. Linear best-fit lines are weighted and circles are sized by the number of respondents. For the figure only, we exclude states with 20 respondents or fewer. All respondents are non-Hispanic White. Note that y-axis ranges vary for each figure. All dependent variables are scaled from 0 to 1.

We find support for these predictions: The historical homicide rate from where respondents grew up consistently predicts responses to the scales in the expected direction for migrants and nonmigrants. Fig. 2 shows state-level scatterplots for a selection (for more outcomes, see *SI Appendix*, Fig. S15). Table 3 presents our individual-level regression models controlling for gender, education, 5-y age-group fixed effects and state of residence fixed effects for migrants. If we group together related estimates with precision weighted averages, we find that all but one are statistically significant at conventional levels. One might think that these differences in views of criminal justice simply reflect partisanship, but controlling for party identification leaves these relationships unchanged.

**Table 3.** Survey findings on the persistence of violent victimization among White, Non-Hispanic internal US migrants and nonmigrants

	Effect of historical homicide rate				
	Non-Mig.		Migrants		Persistence
	Coef.	SE	Coef.	SE	%
Culture of honor DVs					
*Do high hist. hom. state respondents see violence as a constant presence?*					
1. Witness violence growing up (three-item scale)	0.048*	0.020	0.040*	0.020	84
2. Assault risk (three-item scale)	0.065*	0.023	0.094*	0.027	144
3. Belief in a dangerous world (two-item scale)	0.072*	0.014	0.103*	0.021	143
(precision weighted average)	0.064*	0.010	0.076*	0.013	118
*Do they distrust institutions?*					
4. Distrust local government where they grew up (one-item scale)	0.027	0.023	0.035	0.023	
5. Distrust local government where they live now (one-item scale)	0.029	0.022	−0.010	0.024	
6. Distrust police effectiveness and responsiveness where they live now (three-item scale)	0.060*	0.019	0.019	0.018	31
7. Trust family over the police (two-item scale)	0.070*	0.019	0.066*	0.028	95
(precision weighted average)	0.050*	0.010	0.024*	0.011	48
*Do they distrust other people?*					
8. Distrust other people where they grew up (one-item scale)	0.057*	0.019	0.094*	0.028	163
9. Distrust other people where they live now (one-item scale)	0.033	0.029	0.031	0.027	92
(precision weighted average)	0.050*	0.016	0.062*	0.019	123
*Do they own a gun for defense?*					
10. Own gun mainly for protection	0.274*	0.040	0.114*	0.037	42
11. Own gun partly for protection	0.043	0.038	0.007	0.034	17
12. Own gun not for protection	−0.082*	0.017	0.004	0.020	−4
*Do they especially value manliness in men?*					
13. Honor ideology in manhood (three-item scale)	0.092*	0.024	0.109*	0.025	31
*Do they see themselves as fiery and quick tempered?*					
14. Hotheadedness (three-item scale)	0.047*	0.014	0.015	0.020	31
*Do they respond more forcefully to insults and provocations in three scenarios?*					
15. Aggressive self-response in the three threatening scenarios (three-item scale)	0.100*	0.031	0.052	0.029	52
16. Aggressive response by friends where they grew up (three-item scale)	0.110*	0.024	0.091*	0.028	83
17. Aggressive response by typical male/female where they grew up (three-item scale)	0.088*	0.024	0.105*	0.032	120
(precision weighted average)	0.099*	0.015	0.081*	0.017	82
18. Aggressive response average in Kevin scenario (three-item scale)	0.107*	0.025	0.071*	0.026	67
19. Aggressive response average in Emma scenario (three-item scale)	0.090*	0.028	0.084*	0.031	93
20. Aggressive response average in Doug scenario (three-item scale)	0.075*	0.033	0.098*	0.030	130
*Do they see backing down as costly?*					
21. If Kevin and Doug walked away, they would look weak (two-item scale)	0.053*	0.024	0.032	0.030	61
22. If Kevin and Doug walked away, they would not feel like men (two-item scale)	0.053*	0.022	0.047	0.028	88
(precision weighted average)	0.053*	0.016	0.040*	0.020	76
Other potential mechanism DVs					
*Do they disregard the law?*					
23. Legal cynicism (three-item scale)	−0.029	0.019	0.028	0.021	
*Do they report lower living standards?*					
24. Living standard (two-item scale)	−0.001	0.020	−0.02	0.025	
*Did they experience one possible form of social disorganization?*					
25. Raised by both parents	−0.079*	0.029	−0.122*	0.033	154

Note: Each row shows a separate regression where the survey measure (DV) is regressed on the log of the historical homicide rate in the state where respondents grew up, an indicator for whether the respondent is an internal US migrant, the interaction of these two variables, and the number of years in school, with state-of-residence fixed effects for migrants and gender and 5-y age group fixed effects for all respondents. We calculate the migrant coefficient with the main effect and the interaction. Analysis is conducted at the individual level. All variables are rescaled to vary from 0 to 1.

SE are clustered by the state where the respondent grew up. We calculate the persistence percentage as migrant coefficient over nonmigrant coefficient multiplied by 100, only showing those with migrant coefficients above 0.03. *P < 0.05.

See *SI Appendix*, Table S18, for the full models.

The effect sizes are not large, but we think it is notable that, almost a century later, we can still detect reverberations of historical homicide rates in online surveys.

The culture of honor appears to be more prevalent in rural areas than in urban areas (40). Indeed, we find that the associations between our survey measures and historical homicide rates more than double among rural respondents. In *SI Appendix*, Table S19, we show the estimates for each item for rural respondents, and in *SI Appendix*, Fig. S16, we show a precision weighted average for rural respondents (before and after a preregistration update).

Our survey focused on the culture of honor, as it aligned with our observational findings. We did include, however, a few measures to assess economic, legal cynicism, and social disorganization mechanisms, but found mixed support for these (see bottom of Table 3). Future studies should further explore these mechanisms.

## Discussion

Focusing on White interstate migrants who move between safer and less safe states, we find that migrants from historically unsafe states carry the shadow of homicide victimization with them to their new states. We replicate this finding across three distinct periods in US homicide history, observing that approximately 50% of White migrants’ homicide risk from their birth states persists in their new states of residence in 1959–61, around 40% persists in 1979–91, and about 30% persists in 2000–17. Our results are not inconsistent with prior studies showing no direct link between migration and crime or delinquency (41–43). These studies emphasize that migrants are not inherently more or less prone to violence simply by virtue of moving. Our findings align with this view: we do not claim that migration itself increases violent behavior. Rather, we show that among migrants, those from historically violent places carry with them elevated risks—suggesting persistence of norms or environmental influences, not a general effect of migration.

Why does this persistence occur? This remains a challenging question that we cannot resolve, especially given the complexity of historical variables that are inherently interrelated and vary in measurement quality. Nevertheless, research suggests enduring cultural norms shaped by long-term exposure to violence may contribute. In particular, cultures of honor—where individuals emphasize personal reputation for toughness and readiness to respond to threats—might help explain why migrants from historically violent regions remain at higher risk even after relocating. These cultural norms could develop in response to weak or mistrusted institutions or arise for other reasons connected to historical patterns of conflict and self-reliance. Supporting this interpretation of our persistence findings, our large-scale survey of interstate migrants shows traits and behaviors associated with a culture of honor—such as gun ownership for protection, aggressive responses to personal slights, and mistrust of criminal justice institutions—persist even after citizens relocate. However, due to the inherent difficulty of determining mechanisms, any interpretation should be made cautiously.

## Materials and Methods

This section provides an overview of the materials and methods used. Please consult *SI Appendix* for more details. We measure the historical homicide rate for Whites at the earliest point possible. After a several-decade effort, the US Census successfully collected death certificates for nearly all US deaths starting in 1933. We digitized these early state-level counts of homicides from the Census and use the simple average of the first decade of available data, 1933–42, to measure the historical state homicide rate for Whites (44, 45). 1937–41 were already digitized (46). We must rely on state-of-birth homicide rates as our key independent variable, not county or city of birth rates, because we use death certificates to track homicide persistence and they only contain birth states. In part because of the Census’s focus on data quality, early death certificate data appear to accurately measure homicides (47, 48). We observe these early state homicide rates for all states and the District of Columbia, except for Alaska and Hawaii as these were not states at the time.

We relate the historical homicide rate in migrants’ states of birth to several outcomes. The first is migrants’ risk of homicide victimization in the state and county they move to, excluding police homicides. We can track the homicide rate of internal US migrants in 1959–61, because death certificates included state of birth in this period (49). For each homicide victim, we therefore observe state and county of residence, state of birth, race, age, sex, and marital status. The 5% samples from the decennial US Census (50) also record state of birth, allowing us to calculate homicide victimization rates for different migrant groups. The data available in the death certificates allow us to calculate the homicide rates for migrant groups by age group, e.g., 25- to 29-y-olds born in Kentucky and living in Ohio. State of birth is first available in the death registry in 1959–61 and again in 1979 onward (see *SI Appendix*, section S4 for details). In the Census, we also observe education, family income, etc., for each of the migrant groups. We use regression models with logarithmic transformations to study the relationship between historical and present-day homicide rates, as they effectively capture proportional relationships and allow for the interpretation of elasticities; however, count models and other robustness checks reveal similar findings (*SI Appendix*, Tables S21–S26). We cluster our standard errors by state of birth, because this is the level at which our “treatment,” i.e., being born in a safe or unsafe state, operates.

Ideally, we would measure perpetration rates, not just victimization rates, but we lack reliable data on perpetration. However, given the high proportion of homicides that result from interpersonal conflicts, perpetration and victimization rates are usually highly correlated (51–53).

We conduct our analysis of migrant homicide rates in three distinct periods: 1959–61, 1979–91, and 2000–17. As noted above, the earliest of these periods corresponds with the first 3 y in which state of birth is available in the death registry. These data are again available beginning in 1979, so we examine 1979–91 to capture a particularly violent period in the United States. Finally, we examine 2000–17 to investigate whether patterns of violent victimization persist further into history and into another lower-violence period (non-Hispanic Whites). In 1959–61, our data include 181.9 million nonmigrant person-years, 90.9 million migrant person-years, 11,269 nonmigrant homicides, and 7,807 migrant homicides. For the other two periods, these numbers are 962.1 M, 510.4 M, 63,733, and 33,895, and 1,308.4 M, 741.2 M, 44,807, and 26,893, respectively.

Although death certificates do not contain socioeconomic information, the census allows us to measure education and income at the migrant group level. For each group defined by state of birth, destination, age, and sex (e.g., 25- to 29-y-old men born in Kentucky and living in Ohio), we calculate average educational attainment and family income from the census. We then use these averages to classify groups into quartiles of education and income. In these analyses, the dependent variable is unchanged: it is always the homicide mortality rate of each migrant group, calculated from death certificates relative to census population counts. What differs is the set of groups we analyze. For example, when estimating persistence among the top quartile of educational attainment, we restrict attention to the 25 percent of migrant groups with the highest average schooling. In the same way, we can examine persistence among groups with lower education, higher or lower incomes, or relative standing compared to nonmigrants in the receiving area. Because these measures are based on group averages, they cannot reveal which individuals within each group drive the patterns, but they allow us to assess whether persistence is confined to disadvantaged subgroups or extends across the socioeconomic spectrum.

Our survey interviewed non-Hispanic Whites, with an oversample of internal US migrants. To develop the survey measures, we conducted several large pilot studies in 2022 and 2023. We then preregistered and administered the final version of the survey in September 2023 on Lucid Marketplace, sampling only non-Hispanic Whites and oversampling migrants. Before starting the survey, all respondents consented to take the survey and all took the survey voluntarily. All were compensated through the Lucid standard procedures (over which we have no control or knowledge). The survey was approved by UC Berkeley’s Committee for the Protection of Human Subjects (protocol number 2022-02-15068). To maximize our sample size, our analysis combines 3,416 pilot study respondents and 4,078 final version respondents for a total of 7,494, with 3,312 migrants and 4,182 nonmigrants. The results for the pilot and final samples are similar and the sample is broadly demographically representative of non-Hispanic White migrants and nonmigrants, though more female and lower income (*SI Appendix*, Table S27). *SI Appendix*, Tables S28 and S29 present descriptive statistics. Please see *SI Appendix* for survey questions and for the preregistered analysis, which deviates only slightly from the analysis here.

In our analyses of both survey and victimization outcomes, we use the 1933–42 homicide rates. It is important to note that—especially in the later periods of the victimization analyses and for our survey sample—nearly all individuals we analyzed were not yet born. For 1959–61, we explored whether generational models might fit the victimization data better than the persistence models presented here—for instance, linking migrants to the birth-state homicide rate in their youth rather than the historical birth-state rate—but preliminary analysis found that the historical birth-state homicide rate consistently provided a stronger prediction of migrants’ risk of violent death than does the birth-state rate from their youth. Nevertheless, further research on generational models is warranted.

## Supplementary Material

Appendix 01 (PDF)

## Data Availability

Data and code to replicate all results can be found on the project’s OSF page (https://osf.io/pr97f) (54) with the exception of the last period we examine with the death registry data, 2000–17. Although death certificates are public record, the CDC restricts access to recent death certificate data, requires a data user agreement, and prohibits most redistribution. We are allowed to make highly aggregated data available for replication, but full replication of the 2000–17 findings will require a CDC agreement and specialized data protective services from one’s institution. It is important to note that the findings in this third period simply replicate the findings in the two earlier periods we examined with death certificate data, and these earlier data are publicly available.
