# Supplementary material for: Migration and the persistence of violence
Source: Proc Natl Acad Sci U S A. 2025 Nov 24;122(48):e2500535122. doi: 10.1073/pnas.2500535122 (PMC12684885; doi:10.1073/pnas.2500535122)
Supplement: Supplementary file 1 — Appendix 01 (PDF) [file pnas.2500535122.sapp.pdf]

Supplementary Information

Contents

S1 Supplementary Information Tables 1
S2 Supporting Information Figures 32
S3 Data Availability Statement 49
S4 Death Registry and Census Data 49
S5 Correlate Benchmarks 50
S6 Survey 51
S7 Survey Analysis Preregistration 56
S8 Lessons from Pilot Surveys 57
S9 Survey Questions 59
S10 SI Citations 68

S1 Supplementary Information Tables

Table S1: Persistence of Homicide Victimization Rates among White Internal US Migrants Compared to Non-Migrants 1979-91

	1979-91				
	Non-Mig.		Migrants		Persistence
Model	Coef.	SE	Coef.	SE	%
Analysis at the birth-state level (n=49 states of birth)					
Bivariate regression estimates on data in Figure 1	0.86	0.10	0.44	0.05	51
*Analysis at the birth-state * residence-state * age-group level with controls for state-residence * age-group FE for migrants and age-group FEs for non-migrants*					
Baseline regression estimates at this level of disaggregation	0.85	0.11	0.34	0.05	40
Females	0.53	0.07	0.21	0.04	40
Males	1.01	0.14	0.42	0.06	42
Married females	0.52	0.07	0.19	0.04	36
Married males	1.09	0.13	0.46	0.05	43
Unmarried females	0.54	0.09	0.23	0.07	43
Unmarried males	0.95	0.14	0.41	0.08	43
Age less than 15	0.17	0.05	0.02	0.03	12
Age 15-29	0.79	0.11	0.30	0.05	39
Age 30-44	0.93	0.12	0.34	0.05	37
Age 45-59	0.82	0.11	0.38	0.04	46
Age 60-74	0.68	0.08	0.26	0.05	39
Age 75 and up	0.46	0.09	0.17	0.06	36
Analysis at the county by state-of-birth level controlling for age, age squared, male %, and log of group size age-group for county FEs for migrants					
Baseline regression estimates at the county level	1.02	0.20	0.35	0.06	34
Above the median county population	1.02	0.23	0.36	0.06	35
Below the median county population	0.97	0.13	0.24	0.05	25

Note: This table shows estimates of the effect of the log of 1933-42 state-of-birth white homicide rate on the log +1 of homicide rates in 1979-91. Each set of coefficients and standard errors is from a separate regression. In row 1, the Ns are 49. In rows 2-13, the Ns for migrants are 49 states of birth within each of 48 states of residence (including DC minus one) separately for nine five-year age groups (ages 15-59), so (49 times 8 times 9=) 21,168 groups, though we have some missing population data for 190 small migrant groups in small states. The N for non-migrants is 49*9=441. The Ns for the counties are 19,966. The models do not include state of residents fixed effects or county fixed effects for non-migrants because these are colinear with historical homicide rates.

Table S2: Persistence of Homicide Victimization Rates among White Internal US Migrants 1979-91

	1979-91			
Model	Coef.	SE	R2	N
Analysis at the birth-state level (n=49 states of birth)				
Bivariate regression estimates on data in Figure 1	1.28	0.08	0.66	49
*Analysis at the birth-state * residence-state * age-group level with controls for male %, log of group size, and state-residence * age-group FE*				
Baseline regression estimates at this level of disaggregation	0.34	0.04	0.43	21,842
Only migrants who crossed census regions	0.27	0.06	0.42	16,569
Northeastern residence	0.22	0.07	0.26	4,669
Midwestern residence	0.48	0.06	0.34	4,690
Southern residence	0.31	0.05	0.38	6,447
Western residence	0.30	0.08	0.36	6,036
Above median migrant population	0.36	0.05	0.43	10,917
Below median migrant population	0.11	0.03	0.17	10,925
1st quartile years in school among migrants ages 25-59	0.38	0.05	0.49	4,241
2nd quartile years in school	0.22	0.05	0.52	4,241
3rd quartile years in school	0.17	0.06	0.45	4,241
4th quartile years in school	0.12	0.05	0.43	4,240
1st quartile HH income among migrants	0.26	0.04	0.46	5,461
2nd quartile HH income	0.30	0.05	0.45	5,460
3rd quartile HH income	0.33	0.05	0.45	5,461
4th quartile HH income (highest)	0.25	0.06	0.44	5,460
1st quartile HH income inequality between migrant group and non-migrants (migrants relatively best-off)	0.20	0.07	0.46	5,232
2nd quartile HH income inequality between migrant group and non-migrants	0.21	0.05	0.48	5,245
3rd quartile HH income inequality between migrant group and non-migrants	0.19	0.06	0.48	5,228
4th quartile HH income inequality between migrant group and non-migrants (migrants relatively worst-off)	0.42	0.06	0.49	5,259
1st quartile education inequality between migrant group and non-migrants (migrants relatively most-educated)	0.18	0.05	0.49	5,247
2nd quartile education inequality between migrant group and non-migrants	0.23	0.05	0.52	5,211
3rd quartile education inequality between migrant group and non-migrants	0.27	0.07	0.53	5,226
4th quartile education inequality between migrant group and non-migrants (migrants relatively least-educated)	0.34	0.08	0.45	5,280
Analysis at the county by state-of-birth level with controls for county FEs, age, age squared, male %, and log of group size				
Baseline regression estimates at the county level	0.35	0.06	0.47	19,966
Above the median migrant population	0.35	0.06	0.46	9,964
Below the median migrant population	0.08	0.04	0.12	10,002
1st quartile years in school among migrants	0.35	0.08	0.64	4,992
2nd quartile years in school	0.29	0.04	0.50	4,991
3rd quartile years in school	0.16	0.06	0.48	5,024
4th quartile years in school	0.19	0.06	0.47	4,959
1st quartile education inequality between migrants and non-migrants	0.20	0.06	0.45	4,955
2nd quartile education inequality between migrants and non-migrants	0.13	0.05	0.50	4,955
3rd quartile education inequality between migrants and non-migrants	0.29	0.05	0.54	4,955
4th quartile education inequality between migrants and non-migrants	0.33	0.06	0.58	4,955
1st quartile HH income among migrants	0.22	0.05	0.43	4,992
2nd quartile HH income	0.26	0.05	0.46	4,991
3rd quartile HH income	0.30	0.05	0.54	4,992
4th quartile HH income	0.26	0.07	0.55	4,991
1st quartile HH income inequality between migrants and non-migrants	0.22	0.06	0.44	4,955
2nd quartile HH income inequality between migrants and non-migrants	0.28	0.05	0.50	4,955
3rd quartile HH income inequality between migrants and non-migrants	0.32	0.06	0.49	4,955
4th quartile HH income inequality between migrants and non-migrants	0.07	0.06	0.28	4,955

Note: This table shows estimates of the effect of the log of 1933-42 state-of-birth white homicide rate on the log +1 of homicide rates in 1979-91. Each set of coefficients and standard errors is from a separate regression. Except for the first row, SEs are clustered by state of birth.

Table S3: Average Annual Homicide Victimization Rate by State of Birth among Whites in 1933-42, 1959-61, 1979-91, and 2000-17.

	Homicide rate (per 100,000)						
	1933-42	1959-61		1979-91		2000-17	
	All	Non-migrants	Migrants	Non-migrants	Migrants	Non-migrants	Migrants
AL	8.02	6.66	6.52	9.09	9.73	6.18	4.69
AR	6.16	4.80	4.96	8.30	8.32	5.83	5.43
AZ	9.11	9.32	5.43	13.23	10.37	6.66	4.97
CA	4.55	3.80	4.07	11.64	7.94	4.22	4.68
CO	5.23	4.40	3.42	7.73	7.28	3.15	3.23
CT	1.94	1.08	2.33	3.94	4.80	1.60	2.92
DC	4.87	3.33	1.60	14.27	6.53	7.10	2.58
DE	6.79	2.41	3.78	4.88	4.65	2.94	2.30
FL	8.12	5.64	4.34	10.27	8.00	5.28	4.36
GA	7.23	7.22	7.07	9.31	9.14	4.08	5.10
IA	1.74	1.44	2.95	2.34	4.37	1.65	2.47
ID	3.70	1.84	3.56	3.25	5.53	1.99	3.01
IL	5.44	2.06	2.78	5.25	6.12	2.30	3.49
IN	3.82	2.09	3.51	4.73	6.99	3.35	3.43
KS	3.55	2.52	2.65	4.15	6.17	2.88	3.72
KY	12.10	8.07	5.65	9.04	7.76	4.83	4.96
LA	6.37	4.28	4.68	8.04	8.37	5.56	5.25
MA	1.64	1.48	2.06	3.56	4.81	1.53	2.89
MD	2.69	2.35	3.76	5.38	5.53	3.22	3.55
ME	1.62	1.88	2.44	3.16	4.97	2.08	3.07
MI	3.05	2.04	2.68	4.92	7.05	2.44	3.55
MN	1.97	1.34	2.26	2.11	4.71	1.45	2.28
MO	6.70	3.69	3.62	5.99	7.04	3.97	3.51
MS	7.80	4.79	5.44	8.83	8.92	5.99	5.94
MT	4.73	4.17	2.71	4.63	5.05	2.50	3.06
NC	4.82	5.66	6.00	8.26	9.45	5.08	4.31
ND	1.71	0.94	1.88	1.74	4.07	1.04	2.08
NE	2.16	1.48	3.05	2.50	5.01	1.95	2.56
NH	1.43	1.96	1.54	3.13	4.73	1.87	2.90
NJ	2.82	1.65	1.99	3.28	5.07	1.43	3.83
NM	7.90	11.92	7.45	18.89	11.39	7.08	3.93
NV	10.92	1.41	4.29	7.03	5.97	6.28	5.32
NY	3.29	1.66	2.13	6.25	5.48	2.96	2.69
OH	4.67	1.69	3.36	4.09	6.43	3.08	3.67

Table S3: Average Annual Homicide Victimization Rate by State of Birth among Whites in 1933-42, 1959-61, 1979-91, and 2000-17. *(continued)*

	Homicide rate (per 100,000)						
	1933-42	1959-61		1979-91		2000-17	
	All	Non-migrants	Migrants	Non-migrants	Migrants	Non-migrants	Migrants
OK	5.41	4.77	5.32	9.04	7.67	6.51	4.48
OR	3.17	2.12	2.28	5.12	6.21	3.24	3.74
PA	2.79	1.57	2.21	3.37	4.68	2.45	2.53
RI	1.46	0.89	1.34	4.75	4.50	2.17	2.53
SC	7.23	6.95	4.91	9.59	8.84	6.10	4.57
SD	1.48	1.55	2.85	1.92	3.88	1.08	2.01
TN	8.18	6.04	6.66	8.84	9.07	5.37	4.32
TX	7.05	7.13	6.40	14.28	11.10	5.17	4.60
UT	2.87	1.84	2.50	2.95	4.43	1.67	2.60
VA	5.88	5.84	5.19	7.52	7.01	3.56	4.02
VT	1.38	0.71	2.44	3.43	2.95	2.16	2.19
WA	3.68	2.90	3.81	4.55	6.05	3.24	3.95
WI	1.57	1.50	1.92	2.60	5.34	1.69	2.61
WV	8.17	4.99	4.83	8.18	7.13	5.38	4.25
WY	4.88	5.16	1.69	5.21	6.06	1.89	3.73

Note:

This table presents the values shown graphically in Figure 1.

Table S4: Descriptive statistics for 1959-61 at the state-of-birth, state-of-residence, and five-year age-group level. For non-migrants, each row of the data is a state-of-residence age group, e.g., 50-54 year-old Kentuckians. For migrants, each row of data is migrants from a particular state of birth in their current state of residence, e.g., 50-54 Kentuckians living in Illinois. With the 5% Census sample, we lack data smaller migrant groups, e.g., Montanans ages 20-24 living in Vermont. W. Mean stands for weighted mean. White respondents born in the US ages 15-59.

	Lives in Birth State						Migrated from Birth State					
	Mean	W. Mean	SD	Min	Max	N	Mean	W. Mean	SD	Min	Max	N
Homicide count	12.01	22.34	14.72	0.00	102.00	441	0.14	1.25	0.61	0.00	14.00	20240
Homicides per 100,000	3.64	3.20	3.29	0.00	18.87	441	2.85	3.69	23.36	0.00	1666.67	20240
Pop. in 100K	3.75	8.51	4.23	0.04	24.43	441	0.04	0.32	0.11	0.00	1.73	20240
Age	36.95	35.01	12.94	16.61	57.12	441	36.55	36.90	12.76	15.00	59.00	20240
Female	0.51	0.51	0.02	0.42	0.60	441	0.48	0.50	0.22	0.00	1.00	20240
Married	0.74	0.70	0.24	0.05	0.94	441	0.76	0.78	0.29	0.00	1.00	20240
Education in years	12.40	12.46	1.06	8.27	14.33	441	13.65	13.19	1.76	0.00	20.00	20240
Income in $1000s	5.73	6.08	1.10	2.82	9.33	441	7.08	6.70	2.78	−4.34	46.51	20240
Nonmig. minus mig. edu. in years						0	−1.23	−0.55	1.20	−7.79	3.78	19433
Nonmig. minus mig. income in $1000s						0	−1.10	−0.40	1.28	−15.79	3.37	19433
Migrants who crossed census regions	0.00	0.00	0.00	0.00	0.00	441	0.75	0.57	0.43	0.00	1.00	20240
Northeastern residence	0.20	0.31	0.40	0.00	1.00	441	0.19	0.18	0.39	0.00	1.00	20240
Midwestern residence	0.22	0.30	0.42	0.00	1.00	441	0.22	0.23	0.41	0.00	1.00	20240
Southern residence	0.29	0.29	0.45	0.00	1.00	441	0.28	0.25	0.45	0.00	1.00	20240
Western residence	0.29	0.10	0.45	0.00	1.00	441	0.32	0.34	0.46	0.00	1.00	20240

Table S5: Descriptive statistics for 1959-61 at the state-of-birth, county-of-residence level. For non-migrants, each row of the data is a county. For migrants, each row of data is migrants from a particular state of birth in a particular county. Given the low likelihood of a migrant from each state appearing in the 5% census sample in every county outside her birth state, we only observe a small subset of all potential county-birth state combinations. The higher number of maximum homicides in the county data occurs because we are aggregating all homicides across age groups in the county. W. Mean stands for weighted mean. White respondents born in the US ages 15-59.

	Lives in Birth State						Migrated from Birth State					
	Mean	W. Mean	SD	Min	Max	N	Mean	W. Mean	SD	Min	Max	N
Homicide count	7.33	25.11	17.99	0.00	171.00	730	0.27	6.08	2.71	0.00	224.00	21467
Homicides per 100,000	13.04	5.51	24.58	0.00	185.19	730	10.38	9.49	84.09	0.00	3333.33	21467
Pop. in 100K	1.33	8.49	3.09	0.00	45.87	730	0.03	0.57	0.12	0.00	5.16	21467
Age	32.93	34.64	4.00	15.00	54.00	730	35.33	36.96	7.33	15.00	59.00	21467
Education in years	11.95	12.64	1.40	0.00	18.50	730	13.21	12.96	2.28	0.00	20.00	21467
Income in $1000s	5.77	7.41	1.89	1.17	15.34	730	6.82	7.42	3.10	−0.43	51.47	21467
Nonmig. minus mig. edu. in years						0	−0.93	−0.12	2.02	−11.01	13.40	21202
Nonmig.-Mig. income in $1000s						0	−0.45	0.24	2.64	−43.68	7.60	21202
Migrants who crossed census regions	0.00	0.00	0.00	0.00	0.00	730	0.73	0.61	0.44	0.00	1.00	21467
Northeastern residence	0.24	0.39	0.43	0.00	1.00	730	0.24	0.21	0.43	0.00	1.00	21467
Midwestern residence	0.25	0.28	0.44	0.00	1.00	730	0.25	0.24	0.44	0.00	1.00	21467
Southern residence	0.33	0.22	0.47	0.00	1.00	730	0.29	0.18	0.45	0.00	1.00	21467
Western residence	0.17	0.11	0.38	0.00	1.00	730	0.21	0.37	0.41	0.00	1.00	21467

Table S6: Descriptive statistics for 1979-91 at the state-of-birth, state-of-residence, and five-year age-group level. For non-migrants, each row of the data is their state-of-residence age group. For migrants, each row of data is migrants from a particular state of birth in their current state of residence from one of the five-year age groups. With the 5% Census sample, we lack data on smaller migrant groups. W. Mean stands for weighted mean. White respondents born in the US ages 15-59.

	Lives in Birth State						Migrated from Birth State					
	Mean	W. Mean	SD	Min	Max	N	Mean	W. Mean	SD	Min	Max	N
Homicide count	144.52	361.21	243.19	0.00	2043.00	441	1.55	11.02	4.76	0.00	128.00	21842
Homicides per 100,000	6.15	6.62	4.45	0.00	34.13	441	4.95	6.64	21.56	0.00	1666.67	21842
Pop. in 100K	21.82	48.61	24.20	0.10	130.12	441	0.23	1.56	0.56	0.00	9.82	21842
Age	36.96	33.86	12.93	16.90	57.43	441	36.89	36.15	12.84	15.00	59.00	21842
Female	0.50	0.50	0.01	0.45	0.54	441	0.47	0.50	0.25	−1.00	0.99	21842
Married	0.70	0.64	0.29	0.00	0.95	441	0.73	0.72	0.32	0.00	1.00	21842
Education in years	13.18	13.20	1.01	10.93	16.61	441	14.20	14.10	1.48	6.00	22.00	21842
Income in $1000s	24.96	25.76	4.89	15.47	60.38	441	27.87	27.74	11.22	0.00	664.38	21842
Nonmig. minus mig. edu. in years						0	−1.09	−0.93	0.69	−5.13	1.10	20964
Nonmig. minus mig. income in $1000s						0	−2.05	−2.09	3.82	−33.86	22.49	20964
Migrants who crossed regions	0.00	0.00	0.00	0.00	0.00	441	0.76	0.58	0.43	0.00	1.00	21842
Northeastern residence	0.22	0.27	0.42	0.00	1.00	441	0.21	0.17	0.41	0.00	1.00	21842
Midwestern residence	0.22	0.29	0.42	0.00	1.00	441	0.21	0.18	0.41	0.00	1.00	21842
Southern residence	0.31	0.29	0.46	0.00	1.00	441	0.30	0.35	0.46	0.00	1.00	21842
Western residence	0.24	0.15	0.43	0.00	1.00	441	0.28	0.30	0.45	0.00	1.00	21842

Table S7: Descriptive statistics for 1979-91 at the state-of-birth, county-of-residence level. For non-migrants, each row of the data is a county. For migrants, each row of data is migrants from a particular state of birth in a particular county. Given the low likelihood of a migrant from each state appearing in the 5% census sample in every county outside her birth state, we only observe a small subset of all potential county-birth state combinations. The higher number of maximum homicides in the county data occurs because we are aggregating all homicides across age groups in the county. W. Mean stands for weighted mean. White respondents born in the US ages 15-59.

	Lives in Birth State						Migrated from Birth State					
	Mean	W. Mean	SD	Min	Max	N	Mean	W. Mean	SD	Min	Max	N
Homicide count	98.25	352.17	276.84	0.00	3568.00	420	1.08	15.08	4.90	0.00	264.00	19966
Homicides per 100,000	7.38	8.10	7.75	0.00	116.27	420	5.50	7.30	30.48	0.00	2500.00	19966
Pop. in 100K	12.13	35.66	16.91	0.05	171.98	420	0.15	1.88	0.51	0.00	20.86	19966
Age	33.27	33.38	1.69	25.68	36.76	420	35.31	36.58	4.95	15.00	59.00	19966
Female	0.60	0.54	0.17	0.46	0.93	420	0.51	0.51	0.17	0.00	1.00	19966
Education in years	12.80	12.99	0.68	10.88	14.89	420	13.26	13.41	1.39	0.00	22.00	19966
Income in $1000s	51.46	37.72	46.90	17.56	139.00	420	29.59	32.49	14.85	−5.84	139.00	19966
Nonmig. minus mig. edu. in years						0	−0.45	−0.43	1.12	−9.29	13.55	19820
Nonmig.-mig. income in $1000s						0	21.45	−0.53	46.11	−117.99	144.84	19820
Migrants who crossed regions	0.00	0.00	0.00	0.00	0.00	420	0.76	0.64	0.43	0.00	1.00	19966
Northeastern residence	0.23	0.31	0.42	0.00	1.00	420	0.23	0.16	0.42	0.00	1.00	19966
Midwestern residence	0.25	0.29	0.43	0.00	1.00	420	0.24	0.17	0.43	0.00	1.00	19966
Southern residence	0.32	0.20	0.47	0.00	1.00	420	0.32	0.30	0.47	0.00	1.00	19966
Western residence	0.20	0.20	0.40	0.00	1.00	420	0.21	0.38	0.41	0.00	1.00	19966

Table S8: Descriptive statistics for 2000-17 at the state-of-birth, state-of-residence, and five-year age group level. For non-migrants, each row of the data is a state-of-residence age group, e.g., 50-54-year-old Kentuckians. For migrants, each row of data is migrants from a particular state of birth in their current state of residence, e.g., 50-54-year-old Kentuckians living in Illinois. With the 5% Census sample, we lack data on smaller migrant groups, e.g., Montanans ages 20-24 living in Vermont. W. Mean stands for weighted mean. White respondents born in the US ages 15-59.

	Lives in Birth State						Migrated from Birth State					
	Mean	W. Mean	SD	Min	Max	N	Mean	W. Mean	SD	Min	Max	N
Homicides	101.60	182.46	107.65	0.00	590.00	441	1.22	6.88	3.15	0.00	76.00	22035
Homicides per 100,000	3.68	3.42	2.40	0.00	18.47	441	3.53	3.62	9.76	0.00	357.14	22035
Pop. in 100K	29.67	57.12	28.57	0.22	134.83	441	0.34	2.02	0.75	0.00	22.36	22035
Age	36.98	37.61	12.94	16.65	57.02	441	37.04	40.61	12.87	15.00	59.00	22035
Female	0.50	0.50	0.01	0.40	0.54	441	0.51	0.51	0.09	0.00	1.00	22035
Married	0.61	0.61	0.31	0.00	0.91	441	0.63	0.69	0.32	0.00	1.00	22035
Education in years	13.28	13.23	1.08	10.33	16.58	441	14.05	14.18	1.39	5.50	22.00	22035
Income in $1000s	27.25	27.26	13.72	0.02	84.81	441	31.76	35.01	19.48	0.00	472.53	22022
Nonmig. minus mig. edu. in year						0	-1.02	-1.00	0.56	-3.27	0.63	21154
Nonmig. minus mig. income in $1000						0	-7.51	-8.49	6.90	-49.40	8.39	21154
Migrants who crossed census regions	0.00	0.00	0.00	0.00	0.00	441	0.75	0.56	0.43	0.00	1.00	22035
Northeastern residence	0.18	0.23	0.39	0.00	1.00	441	0.18	0.14	0.38	0.00	1.00	22035
Midwestern residence	0.24	0.33	0.43	0.00	1.00	441	0.23	0.19	0.42	0.00	1.00	22035
Southern residence	0.35	0.30	0.48	0.00	1.00	441	0.33	0.42	0.47	0.00	1.00	22035
Western residence	0.22	0.15	0.42	0.00	1.00	441	0.26	0.25	0.44	0.00	1.00	22035

Table S9: Descriptive statistics for 2000-17 at the state-of-birth, county-of-residence level. For non-migrants, each row of the data is a county. For migrants, each row of data is migrants from a particular state of birth in a particular county. Given the low likelihood of a migrant from each state appearing in the 5% Census sample in every county outside her birth state, we observe a small subset of all potential county-birth state combinations. The higher number of maximum homicides in the county occurs because we are aggregating all homicides across age groups in the county. W. Mean stands for weighted mean. White respondents born in the US ages 15-59.

	Lives in Birth State						Migrated from Birth State					
	Mean	W. Mean	SD	Min	Max	N	Mean	W. Mean	SD	Min	Max	N
Homicides	73.99	163.41	118.03	1.00	1387.00	454	1.00	10.53	4.09	0.00	241.00	20591
Homicides per 100,000	3.61	2.75	3.55	0.32	28.32	454	3.86	2.92	16.36	0.00	1000.00	20591
Pop. in 100K	26.94	65.95	32.45	0.93	280.92	454	0.34	4.00	1.12	0.00	36.72	20591
Age	36.58	36.88	4.48	18.91	46.51	454	45.04	47.86	7.59	15.34	59.00	20591
Female	0.51	0.51	0.01	0.46	0.54	454	0.51	0.52	0.11	0.00	1.00	20591
Education in years	11.03	11.23	0.75	8.08	13.83	454	13.38	13.65	1.40	0.00	22.00	20591
Income in $1000s	17.98	19.92	5.74	4.27	55.77	454	29.27	31.97	15.44	-49.69	442.31	20591
Nonmig. minus mig. edu. in year						0	-2.34	-2.89	1.42	-11.24	10.93	20447
Nonmig. minus mig. income in $1000						0	-11.14	-14.10	14.36	-430.00	65.01	20447
Migrants who crossed census regions	0.00	0.00	0.00	0.00	0.00	454	0.74	0.59	0.44	0.00	1.00	20591
Northeastern residence	0.18	0.28	0.39	0.00	1.00	454	0.19	0.14	0.39	0.00	1.00	20591
Midwestern residence	0.24	0.26	0.43	0.00	1.00	454	0.24	0.14	0.43	0.00	1.00	20591
Southern residence	0.41	0.25	0.49	0.00	1.00	454	0.41	0.41	0.49	0.00	1.00	20591
Western residence	0.17	0.22	0.37	0.00	1.00	454	0.16	0.31	0.36	0.00	1.00	20591

Table S10: Alternative Historical Variables and Homicide Persistence: State Income, Employment, and Agricultural Employment.

	Dependent Variable: White Migrant Homicide Rate (per 100,000)								
	**Income Control**			**Employed Percent Control**			**Agricultural Share Control**		
	**1960s**	**1980s**	**2000s**	**1960s**	**1980s**	**2000s**	**1960s**	**1980s**	**2000s**
	1	2	3	4	5	6	7	8	9
Hist. homicide rate	0.457***	0.375***	0.356***	0.592***	0.397***	0.356***	0.475***	0.369***	0.362***
	0.054	0.045	0.049	0.067	0.042	0.045	0.056	0.045	0.050
Hist. income per capita	−0.547***	−0.105	−0.060						
	0.085	0.065	0.071						
Employed share 1930				−0.334	−0.121	−0.263+			
				0.238	0.149	0.155			
Agric. share 1930							1.152***	0.283+	0.072
							0.200	0.156	0.178
Intercept	3.933***	1.988***	1.141*	0.607**	1.387***	0.944***	0.206*	1.270***	0.738***
	0.560	0.433	0.471	0.201	0.128	0.135	0.083	0.063	0.069
N	49	49	49	49	49	49	49	49	49
R-squared	0.806	0.682	0.594	0.647	0.669	0.612	0.786	0.687	0.589

Notes: The dependent variable measures white internal US migrants' homicide rates in their new states in the three periods listed in the column headings. All explanatory variables are for migrants' states of birth. The historical homicide rate is the 1933-1942 white homicide rate used throughout this article from death certificate data. Hist. income per capita is the average per capita income, 1933-1942 (Bureau of Economic Analysis, Table SAINC1). Employed share 1930 is the share of white non-Hispanic individuals aged 16 to 70 who are employed in the 1930 full census file (Ruggles, 2025). Agric. share 1930 is the share of those employed working in agriculture calculated also from the 1930 full census file. This regression table explores how much white migrants' future homicide rates can be explained by their earlier homicide, income, employment and agricultural sector rates. Each column shows a separate least squares regression model. Standard errors below coefficients. Models weighted by state of birth migrant population. Homicide rates and income are logged. 1960s is 1959-1961, 1980s is 1979-1991, and 2000s is 2000-2017.

Table S11: Alternative Historical Variables and Homicide Persistence: Non-White Migrant Share

	**Dependent Variable: Homicide Rate (per 100,000)**		
	**Non-white Migrant Share Control**		
	**1960s**	**1980s**	**2000s**
	1960s	1980s	2000s
Hist. homicide rate	0.461***	0.328***	0.302***
	0.071	0.046	0.053
Non-white migrant share	1.042***	0.511**	0.424*
	0.280	0.175	0.189
Intercept	0.459***	1.349***	0.794***
	0.094	0.061	0.068
N	49	49	49
R-squared	0.717	0.717	0.628

Notes: The dependent variable measures white internal US migrants' homicide rates in their new states in the three periods listed in the column headings. The historical homicide rate is the state-of-birth 1933-1942 white homicide rate used throughout this article from death certificate data. The non-white migrant share is calculated for each state of birth using the 1930 full census file (Ruggles, 2025). For example, South Carolina has the largest value on this variable, 0.63, meaning that 63

Table S12: Full Regression Models for Table 1 1959-61. M indicates migrants. NM indicates non-migrants. Each column corresponds to a regression in a row in Table 1. The dependent variable is the log of homicide rates per 100,000 in 1959-61.

	1M	1NM	2M	2NM	3M	3NM	4M	4NM	5M	5NM	6M	6NM	7M	7NM	8M	8NM
(Intercept)	0.50	−0.24														
	(0.11)	(0.15)														
Birth-state hom. rate 1933-42 (log)	0.60	0.96	0.47	0.94	0.29	0.42	0.53	1.23	0.30	0.41	0.50	1.32	0.10	0.34	0.36	1.09
	(0.06)	(0.09)	(0.04)	(0.09)	(0.05)	(0.08)	(0.05)	(0.11)	(0.04)	(0.09)	(0.05)	(0.10)	(0.06)	(0.09)	(0.08)	(0.15)
Female																
Population (log)																
Age																
Age squared																
Num.Obs.	49	49	20240	441	18691	441	19012	441	17714	441	17329	441	12196	441	12839	441
R2	0.645	0.722	0.234	0.590	0.185	0.237	0.230	0.596	0.156	0.161	0.181	0.597	0.185	0.177	0.209	0.502

	9M	9NM	10M	10NM	11M	11NM	12M	12NM	13M	13NM	14M	14NM	15M	15NM	16M	16NM	17M	17NM
(Intercept)														−7.72		−0.43		−59.10
														(17.64)		(26.96)		(17.99)
Birth-state hom. rate 1933-42 (log)	0.01	0.08	0.49	0.86	0.57	1.08	0.35	0.91	0.27	0.68	0.04	0.62	0.39	0.80	0.43	0.91	0.19	0.56
	(0.03)	(0.05)	(0.05)	(0.10)	(0.05)	(0.10)	(0.06)	(0.09)	(0.06)	(0.07)	(0.06)	(0.15)	(0.05)	(0.17)	(0.06)	(0.21)	(0.05)	(0.14)
Female													−0.19	7.49	−0.04	6.98	−0.17	8.23
													(0.09)	(3.24)	(0.20)	(6.50)	(0.04)	(3.40)
Population (log)													0.23	−0.02	0.24	−0.07	0.18	−0.13
													(0.02)	(0.07)	(0.02)	(0.10)	(0.02)	(0.13)
Age													0.05	−0.12	0.14	−0.50	0.02	2.93
													(0.04)	(0.86)	(0.08)	(1.10)	(0.02)	(0.97)
Age squared													0.00	0.00	0.00	0.01	0.00	−0.04
													(0.00)	(0.01)	(0.00)	(0.02)	(0.00)	(0.01)
Num.Obs.	6861	147	9239	196	9087	196	8390	196	7303	196	3135	98	15936	383	7215	155	8721	228
R2	0.088	0.548	0.275	0.582	0.218	0.628	0.183	0.569	0.158	0.335	0.110	0.211	0.379	0.280	0.377	0.316	0.230	0.254

Table S13: Full Regression Models for Table 1 2000-17. M indicates migrants. NM indicates non-migrants. Each column corresponds to a regression in a row in Table 1. The dependent variable is the log of homicide rates per 100,000 in 2000-17.

	1M	1NM	2M	2NM	3M	3NM	4M	4NM	5M	5NM	6M	6NM	7M	7NM	8M	8NM
(Intercept)	0.92	0.30														
	(0.08)	(0.11)														
Birth-state hom. rate 1933-42 (log)	0.37	0.69	0.21	0.67	0.14	0.46	0.25	0.80	0.08	0.38	0.21	0.68	0.19	0.51	0.26	0.83
	(0.04)	(0.06)	(0.04)	(0.06)	(0.04)	(0.05)	(0.05)	(0.07)	(0.03)	(0.04)	(0.04)	(0.08)	(0.06)	(0.08)	(0.07)	(0.07)
Female																
Population (log)																
Age																
Age squared																
Num.Obs.	49	49	22035	441	21975	441	21967	441	20638	440	20093	440	20996	441	21094	441
R2	0.588	0.720	0.293	0.770	0.172	0.708	0.267	0.740	0.121	0.601	0.178	0.554	0.194	0.824	0.295	0.846

	9M	9NM	10M	10NM	11M	11NM	12M	12NM	13M	13NM	14M	14NM	15M	15NM	16M	16NM	17M	17NM
(Intercept)														-10.67		-23.43		-2.52
														(7.14)		(10.60)		(3.04)
Birth-state hom. rate 1933-42 (log)	0.12	0.25	0.21	0.62	0.27	0.73	0.17	0.66	0.15	0.58	0.12	0.48	0.18	0.61	0.17	0.55	0.20	0.51
	(0.03)	(0.04)	(0.05)	(0.06)	(0.05)	(0.07)	(0.04)	(0.06)	(0.03)	(0.05)	(0.03)	(0.06)	(0.03)	(0.09)	(0.03)	(0.12)	(0.04)	(0.14)
Female													0.43	8.20	0.63	15.37	0.09	6.11
													(0.13)	(5.26)	(0.22)	(7.52)	(0.14)	(2.53)
Population (log)													0.14	-0.08	0.11	0.02	0.23	-0.51
													(0.01)	(0.05)	(0.01)	(0.05)	(0.01)	(0.06)
Age													0.03	-0.03	0.01	0.02	0.07	-0.01
													(0.01)	(0.06)	(0.02)	(0.06)	(0.02)	(0.10)
Age squared													0.00	0.00	0.00	0.00	0.00	0.00
													(0.00)	(0.00)	(0.00)	(0.00)	(0.00)	(0.00)
Num.Obs.	7240	147	7342	147	7345	147	7348	147	7326	147	4856	98	20885	454	10551	227	10334	227
R2	0.175	0.683	0.265	0.795	0.283	0.658	0.297	0.723	0.174	0.685	0.143	0.574	0.435	0.223	0.466	0.210	0.389	0.417

Table S14: Full Regression Models for Table 2, Rows 1-25, 1959-61. Each column corresponds to a regression in a row in Table 2. The dependent variable is the log of homicide rates per 100,000 in 1959-61.

	1	2	3	4	5	6	7	8	9	10	11	12	13	14	15	16	17	18	19	20	21	22	23	24	25
(Intercept)	0.50																								
	(0.11)																								
Birth-state hom. rate 1933-42 (log)	0.60	0.43	0.35	0.14	0.22	0.09	0.24	0.39	0.38	0.15	0.07	0.22	0.20	0.42	0.07	0.06	0.37	0.27	0.46	0.03	0.37	0.22	0.57	0.39	0.33
	(0.06)	(0.05)	(0.17)	(0.07)	(0.06)	(0.04)	(0.10)	(0.07)	(0.07)	(0.06)	(0.05)	(0.06)	(0.06)	(0.07)	(0.08)	(0.06)	(0.05)	(0.06)	(0.05)	(0.02)	(0.07)	(0.07)	(0.08)	(0.10)	(0.09)
Female		−0.06	1.45	0.29	0.22	−0.14	0.08	−0.38	0.30	0.08	−0.09	−0.21	0.02	0.17	−0.26	0.22	−0.16	0.47	−0.17	0.01	−0.02	−0.06	0.26	−0.09	−0.01
		(0.15)	(0.68)	(0.30)	(0.19)	(0.15)	(0.24)	(0.22)	(0.28)	(0.18)	(0.10)	(0.14)	(0.20)	(0.32)	(0.23)	(0.19)	(0.29)	(0.27)	(0.26)	(0.03)	(0.16)	(0.21)	(0.30)	(0.23)	(0.23)
Population (log)		0.26	0.29	0.28	0.23	0.15	0.25	0.32	0.23	0.20	0.12	0.24	0.19	0.29	0.24	0.24	0.28	0.28	0.29	0.05	0.25	0.18	0.20	0.37	0.30
		(0.02)	(0.06)	(0.02)	(0.02)	(0.02)	(0.03)	(0.02)	(0.02)	(0.01)	(0.01)	(0.02)	(0.02)	(0.03)	(0.03)	(0.01)	(0.02)	(0.03)	(0.02)	(0.01)	(0.02)	(0.02)	(0.02)	(0.03)	(0.03)
Num.Obs.	49	20240	1460	3951	5122	5065	2581	5233	6228	6198	5022	4872	4817	4722	4940	4787	4786	4920	9601	10639	15216	3879	4370	5610	6381
R2	0.645	0.311	0.503	0.388	0.321	0.323	0.417	0.436	0.364	0.329	0.204	0.392	0.391	0.356	0.356	0.387	0.448	0.479	0.303	0.100	0.340	0.248	0.300	0.298	0.314

Table S15: Full Regression Models for Table 2, Rows 26-44, 1959-61. Each column corresponds to a regression in a row in Table 2. The dependent variable is the log of homicide rates per 100,000 in 1959-61.

	26	27	28	29	30	31	32	33	34	35	36	37	38	39	40	41	42	43	46
Birth-state hom. rate 1933-42 (log)	0.39	0.41	0.19	0.17	0.09	0.16	0.04	0.15	0.48	0.12	0.31	0.34	0.19	0.09	0.21	0.23	0.12	0.40	0.05
	(0.05)	(0.11)	(0.06)	(0.08)	(0.07)	(0.06)	(0.06)	(0.06)	(0.08)	(0.11)	(0.07)	(0.08)	(0.07)	(0.10)	(0.08)	(0.06)	(0.12)	(0.05)	(0.01)
Female	−0.19	−0.39	−0.08	−0.20	−0.11	−0.14	0.01	−0.17	−0.48	−0.08	−0.13	−0.41	−0.09	−0.12	−0.19	−0.51	−0.17	0.13	0.00
	(0.09)	(0.10)	(0.17)	(0.14)	(0.05)	(0.07)	(0.13)	(0.17)	(0.12)	(0.07)	(0.15)	(0.18)	(0.09)	(0.07)	(0.15)	(0.15)	(0.11)	(0.29)	(0.02)
Population (log)	0.23	0.26	0.20	0.21	0.14	0.15	0.16	0.22	0.26	0.22	0.26	0.24	0.17	0.19	0.21	0.27	0.34	0.25	0.03
	(0.02)	(0.02)	(0.02)	(0.03)	(0.03)	(0.03)	(0.02)	(0.02)	(0.02)	(0.04)	(0.02)	(0.03)	(0.02)	(0.02)	(0.02)	(0.02)	(0.04)	(0.02)	(0.02)
Age	0.05	0.05	0.09	0.00	−0.05	0.03	0.00	0.01	0.07	0.00	0.03	0.17	−0.04	−0.05	0.06	0.01	−0.04	0.23	0.01
	(0.04)	(0.06)	(0.08)	(0.06)	(0.03)	(0.03)	(0.06)	(0.06)	(0.06)	(0.03)	(0.06)	(0.11)	(0.04)	(0.03)	(0.07)	(0.05)	(0.04)	(0.08)	(0.01)
Age squared	0.00	0.00	0.00	0.00	0.00	0.00	0.00	0.00	0.00	0.00	0.00	0.00	0.00	0.00	0.00	0.00	0.00	0.00	0.00
	(0.00)	(0.00)	(0.00)	(0.00)	(0.00)	(0.00)	(0.00)	(0.00)	(0.00)	(0.00)	(0.00)	(0.00)	(0.00)	(0.00)	(0.00)	(0.00)	(0.00)	(0.00)	(0.00)
Num.Obs.	15936	3992	3994	3967	3983	4444	3985	3799	3534	3984	3984	3984	3984	4389	3860	3802	3711	7863	8073
R2	0.379	0.503	0.442	0.447	0.302	0.334	0.345	0.478	0.462	0.455	0.468	0.431	0.466	0.333	0.409	0.463	0.521	0.378	0.092

Table S16: Full Regression Models for Table 2, Rows 1-25, 2000-17. Each column corresponds to a regression in a row in Table 2. The dependent variable is the log of homicide rates per 100,000 in 2000-17.

	1	2	3	4	5	6	7	8	9	10	11	12	13	14	15	16	17	18	19	20	21	22	23	24	25
(Intercept)	0.92																								
	-0.08																								
Birth-state hom. rate 1933-42 (log)	0.37	0.23	0.2	0.21	0.23	0.04	0.23	0.24	0.24	0.11	0.16	0.16	0.16	0.28	0.16	0.09	0.18	0.24	0.24	0.09	0.21	-0.03	0.32	0.27	0.23
	(0.04)	(0.03)	(0.05)	(0.04)	(0.04)	(0.04)	(0.04)	(0.05)	(0.03)	(0.04)	(0.05)	(0.03)	(0.04)	(0.04)	(0.05)	(0.04)	(0.04)	(0.04)	(0.03)	(0.04)	(0.04)	(0.05)	(0.03)	(0.05)	(0.05)
Female		-0.22	-0.35	-0.15	-0.12	0.37	-0.08	0.05	-0.12	-0.07	0.1	-0.16	-0.09	0.22	-0.02	-0.09	-0.55	0.25	-0.36	-0.01	-0.13	0.29	-0.07	-0.21	-0.67
		-0.16	-0.32	-0.35	-0.39	-0.26	-0.24	-0.24	-0.31	-0.28	-0.3	-0.29	-0.26	-0.29	-0.35	-0.29	-0.24	-0.28	-0.28	-0.12	-0.16	-0.2	-0.33	-0.25	-0.33
Population (log)		0.19	0.19	0.16	0.2	0.13	0.24	0.25	0.15	0.13	0.14	0.18	0.22	0.22	0.14	0.2	0.22	0.21	0.16	0.21	0.18	0.17	0.18	0.18	0.2
		-0.02	-0.02	-0.02	-0.02	-0.01	-0.01	-0.01	-0.02	-0.02	-0.01	-0.01	-0.01	-0.02	-0.02	-0.01	-0.02	-0.02	-0.02	-0.02	-0.02	-0.02	-0.01	-0.02	-0.02
Num.Obs.	49	22035	4286	4286	4285	4285	5506	5505	5506	5505	5312	5280	5280	5282	5312	5278	5282	5282	11016	11019	16563	3884	5177	7341	5633
R2	0.588	0.367	0.426	0.405	0.346	0.336	0.369	0.386	0.392	0.348	0.454	0.411	0.429	0.436	0.467	0.36	0.444	0.432	0.375	0.13	0.327	0.392	0.279	0.309	0.289

Table S17: Full Regression Models for Table 2, Rows 26-44, 2000-17. Each column corresponds to a regression in a row in Table 2. The dependent variable is the log of homicide rates per 100,000 in 2000-17.

	26	27	28	29	30	31	32	33	34	35	36	37	38	39	40	41	42	43	44
Birth-state hom. rate 1933-42 (log)	0.18	0.2	0.18	0.13	0.08	0.15	0.11	0.14	0.23	0.19	0.24	0.1	0.1	0.12	0.13	0.19	0.18	0.17	0.06
	(0.03)	(0.07)	(0.04)	(0.03)	(0.04)	(0.04)	(0.04)	(0.03)	(0.06)	(0.06)	(0.04)	(0.04)	(0.03)	(0.04)	(0.04)	(0.03)	(0.05)	(0.03)	(0.03)
Female	0.43	0.12	0.39	0.4	0.35	-0.28	0.48	0.4	-0.04	-0.08	0.53	0.16	0.6	-0.22	0.52	0.44	0.03	0.68	-0.03
	(0.13)	(0.23)	(0.27)	(0.33)	(0.28)	(0.35)	(0.26)	(0.27)	(0.19)	(0.2)	(0.25)	(0.26)	(0.32)	(0.28)	(0.3)	(0.23)	(0.19)	(0.22)	(0.09)
Population (log)	0.14	0.18	0.15	0.11	0.13	0.1	0.13	0.19	0.19	0.22	0.17	0.11	0.1	0.09	0.13	0.21	0.22	0.12	0.24
	(0.01)	(0.01)	(0.01)	(0.01)	(0.01)	(0.01)	(0.01)	(0.01)	(0.01)	(0.01)	(0.01)	(0.01)	(0.01)	(0.01)	(0.01)	(0.01)	(0.01)	(0.01)	(0.02)
Age	0.03	-0.01	0.03	0.07	0.04	0.01	0.11	0.01	0.01	0.02	0.04	0.07	0.04	0.11	0.03	0.01	-0.01	0.02	0
	(0.01)	(0.02)	(0.03)	(0.05)	(0.05)	(0.05)	(0.04)	(0.03)	(0.02)	(0.02)	(0.03)	(0.04)	(0.05)	(0.05)	(0.03)	(0.02)	(0.02)	(0.02)	(0.02)
Age squared	0	0	0	0	0	0	0	0	0	0	0	0	0	0	0	0	0	0	0
	(0)	(0)	(0)	(0)	(0)	(0)	(0)	(0)	(0)	(0)	(0)	(0)	(0)	(0)	(0)	(0)	(0)	(0)	(0)
Num.Obs.	20885	5168	5168	5168	5167	5185	5185	5185	5185	5222	5230	5212	5221	5184	5186	5185	5185	10434	10451
R2	0.435	0.466	0.464	0.508	0.501	0.484	0.479	0.529	0.482	0.418	0.451	0.479	0.512	0.514	0.473	0.504	0.487	0.468	0.166

Table S18: Full Regression Models for Table 3. Each column corresponds to a regression in a row in Table 3.

	1	2	3	4	5	6	7	8	9	10	11	12	13	14	15	16	17	18	19	20	21	22	23	24	25
Female	−0.12	−0.01	0.02	−0.01	0.03	0.02	−0.06	0.01	0.05	0.01	−0.04	−0.02	−0.06	−0.04	−0.15	−0.12	−0.09	−0.14	−0.08	−0.13	−0.05	−0.02	−0.07	−0.03	−0.02
	(0.01)	(0.01)	(0.01)	(0.01)	(0.01)	(0.01)	(0.01)	(0.01)	(0.01)	(0.01)	(0.01)	(0.01)	(0.01)	(0.01)	(0.01)	(0.01)	(0.01)	(0.01)	(0.01)	(0.01)	(0.01)	(0.01)	(0.00)	(0.01)	(0.01)
Birth-state hom. rate 1933-42 (log)	0.05	0.07	0.07	0.03	0.03	0.06	0.07	0.06	0.03	0.27	0.04	−0.08	0.09	0.05	0.10	0.11	0.09	0.11	0.09	0.08	0.05	0.05	−0.03	0.00	−0.08
	(0.02)	(0.02)	(0.01)	(0.02)	(0.02)	(0.02)	(0.02)	(0.02)	(0.03)	(0.04)	(0.04)	(0.02)	(0.02)	(0.01)	(0.03)	(0.02)	(0.02)	(0.03)	(0.03)	(0.03)	(0.02)	(0.02)	(0.02)	(0.02)	(0.03)
Years of edu.	−0.01	−0.01	−0.02	−0.02	−0.02	−0.01	−0.02	−0.02	−0.02	−0.01	0.00	0.00	−0.01	0.00	−0.01	−0.01	−0.01	−0.01	−0.01	−0.01	−0.01	−0.01	0.00	0.02	0.03
	(0.00)	(0.00)	(0.00)	(0.00)	(0.00)	(0.00)	(0.00)	(0.00)	(0.00)	(0.00)	(0.00)	(0.00)	(0.00)	(0.00)	(0.00)	(0.00)	(0.00)	(0.00)	(0.00)	(0.00)	(0.00)	(0.00)	(0.00)	(0.00)	(0.00)
Birth-state hom. times Migrant	−0.01	0.03	0.03	0.01	−0.04	−0.04	0.00	0.04	0.00	−0.16	−0.04	0.09	0.02	−0.03	−0.05	−0.02	0.02	−0.04	−0.01	0.02	−0.02	−0.01	0.06	−0.02	−0.04
	(0.03)	(0.03)	(0.02)	(0.03)	(0.03)	(0.03)	(0.03)	(0.03)	(0.03)	(0.05)	(0.03)	(0.02)	(0.02)	(0.02)	(0.04)	(0.03)	(0.04)	(0.03)	(0.04)	(0.04)	(0.03)	(0.03)	(0.02)	(0.03)	(0.05)
Num.Obs.	6185	4898	4898	7426	7426	7428	7425	7306	4800	7424	7424	7424	7427	7428	6785	6785	6785	6785	6155	6155	5533	5527	7427	7428	7428
R2	0.162	0.034	0.058	0.116	0.066	0.065	0.095	0.092	0.075	0.059	0.018	0.024	0.074	0.125	0.133	0.111	0.105	0.146	0.075	0.110	0.034	0.040	0.132	0.070	0.070

Table S19: Rural Survey Findings on the Persistence of Violent Victimization among White, Non-Hispanic Internal US Migrants and Non-Migrants. Analysis at the individual level with gender and five-year age group fixed effects.

	Effect of Historical Homicide Rate				
	Non-Mig.		Migrants		Persistence
Model	Coef.	SE	Coef.	SE	%
Do high hist. hom. birth state respondents see more violence growing up?					
Witness violence growing up (three-item scale)	0.110*	0.029	0.108*	0.052	98
Do they see the world as more dangerous?					
Assault risk (three-item scale)	0.062	0.038	0.147*	0.047	237
Belief in a Dangerous World (two-item scale)	0.116*	0.042	0.209*	0.063	180
Do they disregard the law?					
Legal Cynicism (three-item scale)	-0.04	0.033	0.017	0.041	-43
Do they report lower living standards?					
Living standard (two-item scale)	-0.052	0.035	-0.101*	0.049	194
Do high hist. hom. birth state respondents distrust institutions?					
Distrust local goverment where they grew up (one-item scale)	0.054	0.041	0.091	0.057	169
Distrust local goverment where they live now (one-item scale)	0.069	0.044	0.108*	0.039	157
Distrust police effectiveness and responsiveness where they live now (three-item scale)	0.064*	0.028	0.025	0.041	39
(precision weighted average)	0.063*	0.021	0.072*	0.025	114
Do high hist. hom. birth state respondents show adaptation to weak institutions?					
Trust family over the police (two-item scale)	0.063	0.036	0.095	0.050	151
Distrust other people where they grew up (one-item scale)	0.109*	0.039	0.201*	0.051	184
Distrust other people where they live now (one-item scale)	0.113*	0.045	0.130*	0.056	115
(precision weighted average)	0.111*	0.030	0.169*	0.038	152
Own gun mainly for protection	0.282*	0.078	0.134	0.077	48
Own gun partly for protection	0.022	0.055	0.038	0.071	
Own gun not for protection	-0.155*	0.030	-0.022	0.045	14
Aggressive self-response in the three threatening scenarios (three-item scale)	0.226*	0.038	0.204*	0.030	90
Aggressive response by friends where they grew up (three-item scale)	0.213*	0.044	0.228*	0.034	107
Aggressive response by typical male/female where they grew up (three-item scale)	0.184*	0.042	0.251*	0.038	136
(precision weighted average)	0.209*	0.024	0.224*	0.019	107
Aggressive response average in Kevin scenario (three-item scale)	0.200*	0.040	0.193*	0.032	96
Aggressive response average in Emma scenario (three-item scale)	0.191*	0.044	0.262*	0.039	137
Aggressive response average in Doug scenario (three-item scale)	0.199*	0.037	0.211*	0.036	106
If Kevin and Doug walked away, they would look weak (two-item scale)	0.194*	0.060	0.041	0.060	21
If Kevin and Doug walked away, they would not feel like men (two-item scale)	0.161*	0.056	0.057	0.077	35
(precision weighted average)	0.176*	0.041	0.047	0.047	27
Honor ideology in manhood (three-item scale)	0.166*	0.035	0.156*	0.052	94
Hotheadedness (three-item scale)	0.064*	0.024	0.046	0.034	72
Raised by both parents	-0.199*	0.044	-0.163*	0.055	82

Note: Each set of coefficients and standard errors is from a separate regression. Standard errors are clustered by state of birth. * p<0.05.

Table S20: Post Updated Registration Rural Survey Findings on the Persistence of Violent Victimization among White, Non-Hispanic Internal US Migrants and Non-Migrants. Analysis at the individual level with gender and five-year age group fixed effects.

	Effect of Historical Homicide Rate				
	Non-Mig.		Migrants		Persistence
Model	Coef.	SE	Coef.	SE	%
Do high hist. hom. birth state respondents see more violence growing up?					
Witness violence growing up (three-item scale)	0.152*	0.061	0.02	0.108	13
Do they see the world as more dangerous?					
Assault risk (three-item scale)	0.033	0.059	0.127	0.082	386
Belief in a Dangerous World (two-item scale)	0.148*	0.060	0.091	0.105	61
Do they disregard the law?					
Legal Cynicism (three-item scale)	-0.075	0.085	0.04	0.115	-53
Do they report lower living standards?					
Living standard (two-item scale)	-0.076	0.058	-0.188*	0.093	247
Do high hist. hom. birth state respondents distrust institutions?					
Distrust local goverment where they grew up (one-item scale)	0.146	0.082	0.026	0.115	18
Distrust local goverment where they live now (one-item scale)	0.167*	0.080	0.102	0.098	61
Distrust police effectiveness and responsiveness where they live now (three-item scale)	0.126	0.067	0.065	0.121	51
(precision weighted average)	0.144*	0.043	0.068	0.063	47
Do high hist. hom. birth state respondents show adaptation to weak institutions?					
Trust family over the police (two-item scale)	-0.021	0.095	-0.016	0.080	
Distrust other people where they grew up (one-item scale)	0.141	0.072	0.076	0.093	54
Distrust other people where they live now (one-item scale)	0.14	0.078	0.092	0.110	66
(precision weighted average)	0.141*	0.053	0.083	0.071	59
Own gun mainly for protection	0.223	0.139	0	0.144	0
Own gun partly for protection	-0.044	0.161	0.176	0.135	-400
Own gun not for protection	-0.136	0.081	-0.102	0.089	75
Aggressive self-response in the three threatening scenarios (three-item scale)	0.194*	0.072	0.17	0.109	88
Aggressive response by friends where they grew up (three-item scale)	0.240*	0.075	0.197	0.104	82
Aggressive response by typical male/female where they grew up (three-item scale)	0.221*	0.067	0.174	0.105	79
(precision weighted average)	0.218*	0.041	0.181*	0.061	83
Aggressive response average in Kevin scenario (three-item scale)	0.183*	0.081	0.165	0.098	91
Aggressive response average in Emma scenario (three-item scale)	0.246*	0.080	0.206	0.110	84
Aggressive response average in Doug scenario (three-item scale)	0.222*	0.067	0.171	0.104	77
If Kevin and Doug walked away, they would look weak (two-item scale)	0.222*	0.095	0.045	0.112	20
If Kevin and Doug walked away, they would not feel like men (two-item scale)	0.218*	0.095	0.009	0.117	4
(precision weighted average)	0.220*	0.067	0.028	0.081	13
Honor ideology in manhood (three-item scale)	0.260*	0.061	0.215	0.115	83
Hotheadedness (three-item scale)	0.025	0.080	0.029	0.091	
Raised by both parents	-0.280*	0.101	-0.084	0.152	30

Note: Each set of coefficients and standard errors is from a separate regression. Standard errors are clustered by state of birth. * $p<0.05$.

Table S21: Fixed Effect Poisson Models of the Persistence of Homicide Victimization Rates among White Internal US Migrants Compared to Non-Migrants

	1959-61					2000-17				
	Non-Mig.		Migrants		Persistence	Non-Mig.		Migrants		Persistence
Model	Coef.	SE	Coef.	SE	%	Coef.	SE	Coef.	SE	%
Analysis at the birth-state level (n=49 states of birth)										
Bivariate regression estimates	1.31*	0.04	0.78*	0.04	60	0.69*	0.06	0.37*	0.04	54
*Analysis at the birth-state * residence-state * age-group level with controls for state-residence * age-group FE for migrants and age-group FE for non-migrants*										
Baseline regression estimates at this level of disaggregation	1.32*	0.13	0.69*	0.07	53	0.67*	0.06	0.21*	0.04	31
Females	0.70*	0.12	0.57*	0.09	82	0.46*	0.05	0.14*	0.04	30
Males	1.57*	0.14	0.75*	0.09	48	0.80*	0.07	0.25*	0.05	31
Married females	0.69*	0.15	0.64*	0.09	93	0.38*	0.04	0.08*	0.03	21
Married males	1.70*	0.15	0.76*	0.09	45	0.68*	0.08	0.21*	0.04	31
Unmarried females	0.70*	0.13	0.48*	0.17	68	0.51*	0.08	0.19*	0.06	37
Unmarried males	1.45*	0.17	0.79*	0.12	55	0.83*	0.07	0.26*	0.07	31
Age less than 15	0.21	0.13	-0.12	0.21	-57	0.25*	0.04	0.12*	0.03	48
Age 15-29	1.29*	0.15	0.76*	0.10	59	0.62*	0.06	0.21*	0.05	34
Age 30-44	1.38*	0.11	0.78*	0.09	56	0.73*	0.07	0.27*	0.05	37
Age 45-59	1.24*	0.11	0.52*	0.09	42	0.66*	0.06	0.17*	0.04	26
Age 60-74	0.99*	0.09	0.37*	0.11	37	0.58*	0.05	0.15*	0.03	26
Age 75 and up	1.04*	0.23	0.06	0.22	6	0.48*	0.06	0.12*	0.03	25
Analysis at the county by state-of-birth level with controls for age, age squared, male %, log of group size, and county FE for migrants										
Baseline regression estimates at the county level	1.30*	0.24	0.66*	0.06	51	0.61*	0.09	0.18*	0.03	30
Above the median county population	1.43*	0.29	0.64*	0.07	45	0.55*	0.12	0.17*	0.03	31
Below the median county population	0.97*	0.21	0.72*	0.17	74	0.51*	0.14	0.20*	0.04	39

Note: This table shows estimates of the effect of the log of 1933-42 state-of-birth white homicide rate on homicide counts in 1959-61 and 2000-17. Each set of coefficients and standard errors are from separate models of homicide counts. Except for the first row, SEs are clustered by state of birth. Homicide rates are logged. In row 1, the Ns are 49. In row 2, the Ns for migrants are 49 states of birth (including DC, but excluding AK and HI) within each of 51 states of residence (including DC) separately for nine five-year age groups (ages 15-59), so (49 times (51-1) times 9=) 22,050 groups, though we have missing population data for small migrant groups especially in small states—1,810 groups in 1959-61 and 15 in 2000-17. It is 51-1 in this calculation since each of the 49 states of birth can pair with 51-1 possible migrant groups, not 51, because those born in their state of residence can't be migrants in their own state. For counties in 1959-61, we use all data points for which the 5% census files in 1980 and 1990 contains respondents. In 1959-61, this yields an N of 383 non-migrant counties and 15,936 migrant groups in US counties. In 2000-17, these Ns are 454 and 21,866. The models do not include state of residents fixed effects or county fixed effects for non-migrants because these are colinear with the historical homicide rate. * $p<0.05$.

Table S22: Fixed Effect Negative Binomial Models of the Persistence of Homicide Victimization among White Internal US Migrants Compared to Non-Migrants

	1959-61					2000-17				
	Non-Mig.		Migrants		Persistence	Non-Mig.		Migrants		Persistence
Model	Coef.	SE	Coef.	SE	%	Coef.	SE	Coef.	SE	%
Analysis at the birth-state level (n=49 states of birth)										
Bivariate regression estimates	1.28*	0.09	0.76*	0.08	59	0.69*	0.06	0.37*	0.04	54
*Analysis at the birth-state * residence-state * age-group level with controls for the log of group size state-residence * age-group FE for migrants and age-group FE for non-migrants*										
Baseline regression estimates at this level of disaggregation	1.28*	0.08	0.69*	0.07	54	0.67*	0.06	0.21*	0.04	31
Females	0.68*	0.11	0.57*	0.09	84	0.46*	0.05	0.14*	0.04	30
Males	1.54*	0.09	0.75*	0.09	49	0.80*	0.07	0.25*	0.05	31
Married females	0.68*	0.14	0.64*	0.09	95	0.38*	0.04	0.08*	0.03	21
Married males	1.70*	0.10	0.76*	0.09	45	0.68*	0.08	0.21*	0.04	31
Unmarried females	0.70*	0.13	0.48*	0.17	68	0.51*	0.08	0.19*	0.06	37
Unmarried males	1.45*	0.12	0.79*	0.12	55	0.83*	0.07	0.26*	0.07	31
Age less than 15	0.19	0.14	-0.14	0.21	-75	0.25*	0.04	0.12*	0.03	48
Age 15-29	1.22*	0.10	0.76*	0.10	63	0.62*	0.06	0.21*	0.05	34
Age 30-44	1.37*	0.09	0.78*	0.09	57	0.73*	0.07	0.27*	0.05	37
Age 45-59	1.23*	0.09	0.52*	0.09	42	0.66*	0.06	0.17*	0.04	26
Age 60-74	1.00*	0.09	0.37*	0.11	37	0.58*	0.05	0.15*	0.03	26
Age 75 and up	1.04*	0.23	0.06	0.23	6	0.48*	0.06	0.12*	0.03	25
Analysis at the county by state-of-birth level with controls for age, age squared, male %, log of group size, and county FE for migrants										
Baseline regression estimates at the county level	1.20*	0.17	0.66		55	0.61*	0.09	0.18*	0.03	30
Above the median county population	1.36*	0.22	0.64		47	0.55*	0.12	0.17*	0.03	31
Below the median county population	0.96*	0.21	0.72		75	0.51*	0.14	0.20*	0.04	39

Note: This table shows estimates of the effect of the log of 1933-42 state-of-birth white homicide rate on homicide counts in 1959-61 and 2000-17. Except for the first row, SEs are clustered by state of birth. In 1959-61, the county models fail to converge for migrants. In row 1, the Ns are 49. In row 2, the Ns for migrants are 49 states of birth (including DC, but excluding AK and HI) within each of 51 states of residence (including DC) separately for nine five-year age groups (ages 15-59), so (49 times (51-1) times 9=) 22,050 groups, though we have missing population data for small migrant groups especially in small states—1,810 groups in 1959-61 and 15 in 2000-17. It is 51-1 in this calculation since each of the 49 states of birth can pair with 51-1 possible migrant groups, not 51, because those born in their state of residence can't be migrants in their own state. For counties in 1959-61, we use all data points for which the 5% census files in 1980 and 1990 contains respondents. In 1959-61, this yields an N of 383 non-migrant counties and 15,936 migrant groups in US counties. In 2000-17, these Ns are 454 and 21,866. The models do not include state of residents fixed effects or county fixed effects for non-migrants because these are colinear with the historical homicide rate. * p<0.05.

Table S23: Any Homicide Models—Persistence of Homicide Victimization Rates among White Internal US Migrants Compared to Non-Migrants 1959-61

	Non-Mig.		Migrants	
Model	Coef.	SE	Coef.	SE
State Level				
Baseline regression estimates at this level of disaggregation	0.044*	0.006	0.095*	0.045
Females	0.020*	0.003	0.168*	0.046
Males	0.043*	0.006	0.188*	0.040
Married females	0.017*	0.003	0.164*	0.043
Married males	0.036*	0.005	0.259*	0.038
Unmarried females	0.010*	0.003	0.209*	0.047
Unmarried males	0.029*	0.005	0.329*	0.041
Age less than 15	-0.002	0.003	-0.01	0.060
Age 15-29	0.038*	0.007	0.094*	0.046
Age 30-44	0.056*	0.008	0.113	0.062
Age 45-59	0.040*	0.007	0.097	0.057
Age 60-74	0.018*	0.004	0.259*	0.051
Age 75 and up	0.006	0.004	0.280*	0.072
County level				
Baseline regression estimates at the county level	0.032*	0.004	0.107*	0.044
Above the median county population	0.049*	0.008	0.047	0.038
Below the median county population	0.019*	0.004	0.134*	0.062

Note: Least squares regression of an indicator for greater than zero homicides in 1959-61 (DV) on the 1933-42 state-of-birth white homicide rate (log), with each set of coefficients and standard errors from a separate regression. State level analysis is at the birth-state * residence-state * age-group level with controls for state-residence * age-group FE for migrants and age-group FEs for non-migrants. County level analysis at the state-of-birth * county level controlling for age, age squared, male %, and log of group size age-group for county FEs for migrants. Standard errors are clustered by state of birth. Data weighted by population. See the note to Table 1 for additional details.

Table S24: Any Homicide Models—Persistence of Homicide Victimization Rates among White Internal US Migrants Compared to Non-Migrants 1979-91

	Non-Mig.		Migrants	
Model	Coef.	SE	Coef.	SE
State level				
Baseline regression estimates at this level of disaggregation	0	0.000	0.038	0.028
Females	0	0.002	0.063	0.037
Males	0.001	0.001	0.06	0.031
Married females	0.011	0.008	0.089*	0.039
Married males	0.007	0.005	0.131*	0.033
Unmarried females	0.004	0.004	0.053	0.042
Unmarried males	0	0.001	0.042	0.035
Age less than 15	0	0.000	0.021	0.024
Age 15-29	0	0.000	0.023	0.013
Age 30-44	0	0.000	0.033*	0.014
Age 45-59	-0.003	0.004	0.059*	0.013
Age 60-74	0.015	0.012	0.051*	0.015
Age 75 and up	-0.002	0.003	0.056*	0.028
County level				
Baseline regression estimates at the county level	0.036	0.034	0.049*	0.012
Above the median county population	0.046	0.045	0.046*	0.013
Below the median county population	0.025*	0.010	0.055*	0.017

Note: Least squares regression of an indicator for greater than zero homicides in 1979-91 (DV) on the 1933-42 state-of-birth white homicide rate (log), with each set of coefficients and standard errors from a separate regression. State level analysis is at the birth-state * residence-state * age-group level with controls for state-residence * age-group FE for migrants and age-group FEs for non-migrants. County level analysis at the state-of-birth * county level controlling for age, age squared, male %, and log of group size age-group for county FEs for migrants. Standard errors are clustered by state of birth. Weighted by population. See the note to the main 1979-91 SI Table for additional details.

Table S25: Log of Homicide Models Excluding Zeros—Persistence of Homicide Victimization Rates among White Internal US Migrants Compared to Non-Migrants 1959-61

	Non-Mig.		Migrants	
Model	Coef.	SE	Coef.	SE
State Level				
Baseline regression estimates at this level of disaggregation	1.163*	0.083	0.212*	0.028
Females	0.423*	0.085	0.084*	0.039
Males	1.276*	0.087	0.203*	0.035
Married females	0.378*	0.099	0.058	0.046
Married males	1.280*	0.096	0.167*	0.035
Unmarried females	0.181	0.094	0.016	0.046
Unmarried males	0.934*	0.109	0.094*	0.042
Age less than 15	0.207	0.142	-0.001	0.044
Age 15-29	1.088*	0.108	0.304*	0.048
Age 30-44	1.290*	0.100	0.269*	0.037
Age 45-59	1.135*	0.074	0.118*	0.045
Age 60-74	0.772*	0.115	-0.007	0.046
Age 75 and up	0.34	0.212	0.004	0.116
County level				
Baseline regression estimates at the county level	-22.28	11.982	0.185*	0.045
Above the median county population	-49.752*	16.759	0.201*	0.046
Below the median county population	-6.018	15.686	0.049	0.062

Note: Least squares regression of log(homicides) excluding zeros in 1959-61 (DV) on the 1933-42 state-of-birth white homicide rate (log), with each set of coefficients and standard errors from a separate regression. State level analysis is at the birth-state * residence-state * age-group level with controls for state-residence * age-group FE for migrants and age-group FEs for non-migrants. County level analysis at the state-of-birth * county level controlling for age, age squared, male %, and log of group size age-group for county FEs for migrants. Standard errors are clustered by state of birth. Data weighted by population. See the note to Table 1 for additional details.

Table S26: Log of Homicide Models Excluding Zeros—Persistence of Homicide Victimization Rates among White Internal US Migrants Compared to Non-Migrants 1979-91

	Non-Mig.		Migrants	
Model	Coef.	SE	Coef.	SE
State level				
Baseline regression estimates at this level of disaggregation	1.163*	0.083	0.212*	0.028
Females	0.423*	0.085	0.084*	0.039
Males	1.276*	0.087	0.203*	0.035
Married females	0.378*	0.099	0.058	0.046
Married males	1.280*	0.096	0.167*	0.035
Unmarried females	0.181	0.094	0.016	0.046
Unmarried males	0.934*	0.109	0.094*	0.042
Age less than 15	0.207	0.142	-0.001	0.044
Age 15-29	1.088*	0.108	0.304*	0.048
Age 30-44	1.290*	0.100	0.269*	0.037
Age 45-59	1.135*	0.074	0.118*	0.045
Age 60-74	0.772*	0.115	-0.007	0.046
Age 75 and up	0.34	0.212	0.004	0.116
County level				
Baseline regression estimates at the county level	-22.28	11.982	0.185*	0.045
Above the median county population	-49.752*	16.759	0.201*	0.046
Below the median county population	-6.018	15.686	0.049	0.062

Note: Least squares regression of log(homicides) excluding zeros in 1979-91 (DV) on the 1933-42 state-of-birth white homicide rate (log), with each set of coefficients and standard errors from a separate regression. State level analysis is at the birth-state * residence-state * age-group level with controls for state-residence * age-group FE for migrants and age-group FEs for non-migrants. County level analysis at the state-of-birth * county level controlling for age, age squared, male %, and log of group size age-group for county FEs for migrants. Standard errors are clustered by state of birth. Weighted by population. See the note to the main 1979-91 SI Table for additional details.

Table S27: Survey Demographics Benchmarked to Census' 2021 American Community Survey (five-year average)

	**Migrants**		**Non-Migrants**	
**Sample**	**Lucid**	**ACS 2021**	**Lucid**	**ACS 2021**
N	3,312	3,205,692	4,182	4,903,830
Gender				
Female	58	51	56	51
Age				
18-24	5	7	9	12
25-34	11	14	16	17
35-49	27	22	29	23
50-64	34	28	29	26
65+	23	29	18	23
Education				
No HS Degree	3	4	3	6
HS Graduate	22	30	26	40
Some College / 2-year Degree	34	24	33	25
Bachelor's Degree	25	25	23	19
Graduate Degree	15	17	14	10
Income				
<$20k	19	7	22	8
$20k-$39k	28	11	26	12
$40k-$59k	21	12	20	13
$60k-$79k	12	12	11	13
$80k+	20	57	22	54

Note: Except for the N row, cell entries provide the percentage of each sample present in each demographic category. We include respondents from the ACS 2021 who are 18 and over and who identified as white non-Hispanic. The Lucid survey defines migrants as people who say they grew up in a state other than the one they currently live in. In the ACS, we use whether they were born in a state other than the one they currently live in (our only option). We exclude in both samples respondents born outside the United States. The ACS sample is weighted.

Table S28: Survey Descriptive Statistics.

	Lives in Birth State					Migrated from Birth State				
	Mean	SD	Min	Max	N	Mean	SD	Min	Max	N
Witness violence growing up (3-item scale)	0.374	0.275	0.000	1.000	3311	0.343	0.279	0.000	1.000	2929
Assault risk (3-item scale)	0.305	0.258	0.000	1.000	2430	0.308	0.262	0.000	1.000	2515
Belief in a dangerous World (2-item scale)	0.616	0.244	0.000	1.000	2430	0.612	0.252	0.000	1.000	2515
Legal cynicism (3-item scale)	0.340	0.245	0.000	1.000	4181	0.300	0.230	0.000	1.000	3312
Living standard (2-item scale)	0.558	0.258	0.000	1.000	4182	0.548	0.267	0.000	1.000	3312
Distrust local government where they grew up (1 item)	0.443	0.286	0.000	1.000	4181	0.439	0.293	0.000	1.000	3311
Distrust local government where they live now (1 item)	0.509	0.282	0.000	1.000	4181	0.520	0.280	0.000	1.000	3311
Distrust police effectiveness and responsiveness where they live now (3-item scale)	0.420	0.250	0.000	1.000	4182	0.445	0.252	0.000	1.000	3312
Trust family over the police (2-item scale)	0.413	0.331	0.000	1.000	4180	0.418	0.313	0.000	1.000	3311
Distrust other people where they grew up (1 item)	0.433	0.299	0.000	1.000	4118	0.437	0.298	0.000	1.000	3254
Distrust other people where they live now (1 item)	0.496	0.297	0.000	1.000	2387	0.521	0.296	0.000	1.000	2460
Own gun mainly for protection	0.281	0.450	0.000	1.000	4180	0.290	0.454	0.000	1.000	3310
Own gun partly for protection	0.152	0.359	0.000	1.000	4180	0.142	0.349	0.000	1.000	3310
Own gun not for protection	0.056	0.229	0.000	1.000	4180	0.051	0.221	0.000	1.000	3310
Aggressive self-response in the three threatening scenarios (3-item scale)	0.417	0.315	0.000	1.000	3738	0.354	0.298	0.000	1.000	3108
Aggressive response by friends where they grew up (3-item scale)	0.527	0.306	0.000	1.000	3738	0.492	0.301	0.000	1.000	3108
Aggressive response by typical male/female where they grew up (3-item scale)	0.569	0.307	0.000	1.000	3738	0.532	0.310	0.000	1.000	3108
Aggressive response average in Kevin scenario (3-item scale)	0.470	0.311	0.000	1.000	3738	0.426	0.303	0.000	1.000	3108
Aggressive response average in Emma scenario (3-item scale)	0.581	0.322	0.000	1.000	3297	0.537	0.322	0.000	1.000	2915
Aggressive response average in Doug scenario (3-item scale)	0.479	0.309	0.000	1.000	3297	0.430	0.294	0.000	1.000	2915
If Kevin and Doug walked away, they would look weak (2-item scale)	0.499	0.312	0.000	1.000	2870	0.480	0.314	0.000	1.000	2715
If Kevin and Doug walked away, they would not feel like real men (2-item scale)	0.503	0.306	0.000	1.000	2867	0.475	0.312	0.000	1.000	2712
Honor ideology in manhood (3-item scale)	0.478	0.283	0.000	1.000	4182	0.463	0.283	0.000	1.000	3311
Hot headedness (3-item scale)	0.288	0.254	0.000	1.000	4182	0.261	0.248	0.000	1.000	3312
Raised by both parents (one item)	0.723	0.448	0.000	1.000	4182	0.714	0.452	0.000	1.000	3312

Table S29: Correlations between Survey Measures

Measure	1	2	3	4	5	6	7	8	9	10	11	12	13	14	15	16	17	18	19	20	21	22	23	24	25
1. Witness violence growing up (3-item scale)	—	—	—	—	—	—	—	—	—	—	—	—	—	—	—	—	—	—	—	—	—	—	—	—	—
2. Assault risk (3-item scale)	.38	—	—	—	—	—	—	—	—	—	—	—	—	—	—	—	—	—	—	—	—	—	—	—	—
3. Belief in a dangerous World (2-item scale)	.28	.35	—	—	—	—	—	—	—	—	—	—	—	—	—	—	—	—	—	—	—	—	—	—	—
4. Legal cynicism (3-item scale)	.30	.22	.10	—	—	—	—	—	—	—	—	—	—	—	—	—	—	—	—	—	—	—	—	—	—
5. Living standard (2-item scale)	-.10	-.13	-.16	-.07	—	—	—	—	—	—	—	—	—	—	—	—	—	—	—	—	—	—	—	—	—
6. Distrust local government where they grew up (1 item)	.27	.21	.32	.19	-.24	—	—	—	—	—	—	—	—	—	—	—	—	—	—	—	—	—	—	—	—
7. Distrust local government where they live now (1 item)	.13	.13	.24	.12	-.35	.51	—	—	—	—	—	—	—	—	—	—	—	—	—	—	—	—	—	—	—
8. Distrust police effectiveness and responsiveness where they live now (3-item scale)	.16	.17	.18	.15	-.33	.39	.48	—	—	—	—	—	—	—	—	—	—	—	—	—	—	—	—	—	—
9. Trust family over the police (2-item scale)	.30	.19	.19	.26	-.15	.34	.25	.40	—	—	—	—	—	—	—	—	—	—	—	—	—	—	—	—	—
10. Distrust other people where they grew up (1 item)	.25	.29	.47	.12	-.22	.46	.30	.31	.27	—	—	—	—	—	—	—	—	—	—	—	—	—	—	—	—
11. Distrust other people where they live now (1 item)	.12	.18	.32	.07	-.35	.30	.49	.41	.17	.57	—	—	—	—	—	—	—	—	—	—	—	—	—	—	—
12. Own gun mainly for protection	.15	.10	.14	.06	-.01	.11	.07	.06	.12	.10	.10	—	—	—	—	—	—	—	—	—	—	—	—	—	—
13. Own gun partly for protection	.03	-.02	.01	.00	-.01	.01	.02	.03	.03	-.01	-.02	-.26	—	—	—	—	—	—	—	—	—	—	—	—	—
14. Own gun not for protection	-.06	-.08	-.09	-.06	.04	-.04	-.04	-.02	-.04	-.06	-.07	-.15	-.10	—	—	—	—	—	—	—	—	—	—	—	—
15. Aggressive self-response in the three threatening scenarios (3-item scale)	.43	.27	.24	.34	-.04	.19	.09	.13	.31	.18	.12	.15	.04	-.07	—	—	—	—	—	—	—	—	—	—	—
16. Aggressive response by friends where they grew up (3-item scale)	.48	.33	.30	.29	-.08	.23	.12	.18	.33	.25	.14	.15	.05	-.04	.74	—	—	—	—	—	—	—	—	—	—
17. Aggressive response by typical male/female where they grew up (3-item scale)	.48	.35	.34	.26	-.10	.26	.14	.19	.31	.28	.16	.14	.04	-.05	.66	.86	—	—	—	—	—	—	—	—	—
18. Aggressive response average in Kevin scenario (3-item scale)	.49	.33	.29	.33	-.08	.25	.13	.17	.34	.23	.14	.15	.05	-.05	.80	.86	.83	—	—	—	—	—	—	—	—
19. Aggressive response average in Emma scenario (3-item scale)	.41	.27	.29	.24	-.08	.22	.12	.16	.27	.24	.15	.15	.04	-.07	.77	.83	.82	.67	—	—	—	—	—	—	—
20. Aggressive response average in Doug scenario (3-item scale)	.45	.32	.28	.30	-.06	.20	.09	.14	.29	.21	.12	.13	.04	-.05	.79	.86	.82	.73	.67	—	—	—	—	—	—
21. If Kevin and Doug walked away, they would look weak (2-item scale)	.29	.21	.18	.21	-.04	.15	.08	.11	.21	.13	.06	.10	.02	-.06	.41	.43	.44	.46	.37	.42	—	—	—	—	—
22. If Kevin and Doug walked away, they would not feel like real men (2-item scale)	.28	.20	.19	.23	-.05	.16	.10	.11	.20	.13	.06	.09	.02	-.06	.38	.39	.41	.42	.34	.39	.74	—	—	—	—
23. Honor ideology in manhood (3-item scale)	.43	.29	.31	.28	-.04	.19	.10	.14	.32	.24	.13	.15	.02	-.06	.50	.55	.56	.55	.49	.52	.44	.43	—	—	—
24. Hot headedness (3-item scale)	.35	.25	.18	.35	-.13	.20	.13	.17	.27	.20	.13	.09	.02	-.05	.41	.33	.31	.37	.29	.34	.24	.26	.33	—	—
25. Raised by both parents (one item)	-.20	-.11	-.13	-.08	.12	-.20	-.14	-.14	-.15	-.18	-.14	-.06	.03	.03	-.11	-.14	-.15	-.14	-.13	-.12	-.08	-.07	-.13	-.13	—

Table S30: Survey Findings on the Persistence of Violent Victimization among White, Non-Hispanic Internal US Migrants and Non-Migrants. This table shows the pre-registered version of Table 3. Originally, we presented the table below as Table 3, but based on a reviewer request, we changed the specification of Table 3. We also changed the layout of Table 3 for clarity. This table maintains the layout and specification of our pre-registered table, as do the tables below. Please see SI Sections S6 and S7 for details.

	Effect of Historical Homicide Rate				
	Non-Mig.		Migrants		Persistence
Model	Coef.	SE	Coef.	SE	%
Do high hist. hom. birth state respondents see more violence growing up?					
1. Witness violence growing up (three-item scale)	0.054*	0.019	0.036	0.021	67
Do they see the world as more dangerous?					
2. Assault risk (three-item scale)	0.065*	0.022	0.086*	0.025	132
3. Belief in a dangerous world (two-item scale)	0.071*	0.014	0.117*	0.020	165
Do they disregard the law?					
4. Legal cynicism (three-item scale)	-0.028	0.019	0.012	0.021	
Do they report lower living standards?					
5. Living standard (two-item scale)	-0.009	0.021	-0.027	0.026	
Do high hist. hom. birth state respondents distrust institutions?					
6. Distrust local goverment where they grew up (one-item scale)	0.037	0.025	0.051*	0.022	139
7. Distrust local goverment where they live now (one-item scale)	0.038	0.024	0.012	0.023	33
8. Distrust police effectiveness and responsiveness where they live now (three-item scale)	0.065*	0.019	0.037*	0.017	58
(precision weighted average)	0.050*	0.013	0.035*	0.012	70
Do high hist. hom. birth state respondents show adaptation to weak institutions?					
9. Trust family over the police (two-item scale)	0.078*	0.020	0.083*	0.027	106
10. Distrust other people where they grew up (one-item scale)	0.064*	0.020	0.105*	0.028	163
11. Distrust other people where they live now (one-item scale)	0.034	0.030	0.041	0.024	120
(precision weighted average)	0.054*	0.017	0.068*	0.018	125
12. Own gun mainly for protection	0.281*	0.040	0.162*	0.034	58
13. Own gun partly for protection	0.045	0.038	0.032	0.037	70
14. Own gun not for protection	-0.083*	0.017	-0.007	0.022	9
15. Aggressive self-response in the three threatening scenarios (three-item scale)	0.105*	0.030	0.058*	0.028	56
16. Aggressive response by friends where they grew up (three-item scale)	0.115*	0.023	0.091*	0.029	79
17. Aggressive response by typical male/female where they grew up (three-item scale)	0.092*	0.023	0.108*	0.032	117
(precision weighted average)	0.104*	0.014	0.084*	0.017	81
18. Aggressive response average in Kevin scenario (three-item scale)	0.113*	0.024	0.079*	0.027	71
19. Aggressive response average in Emma scenario (three-item scale)	0.095*	0.027	0.084*	0.033	89
20. Aggressive response average in Doug scenario (three-item scale)	0.078*	0.032	0.093*	0.031	118
21. If Kevin and Doug walked away, they would look weak (two-item scale)	0.052*	0.024	0.023	0.028	44
22. If Kevin and Doug walked away, they would not feel like men (two-item scale)	0.053*	0.022	0.041	0.026	77
(precision weighted average)	0.053*	0.016	0.033	0.019	62
23. Honor ideology in manhood (three-item scale)	0.097*	0.022	0.108*	0.027	111
24. Hotheadedness (three-item scale)	0.049*	0.014	0.025	0.019	51
25. Raised by both parents	-0.094*	0.030	-0.138*	0.036	147

Note: Each row shows a separate regression where the survey measure (DV) is regressed on the log of the historical homicide rate in the state where respondents grew up, an indicator for whether the respondent is an internal US migrant, and the interaction of these two variables, with gender and five-year age group fixed effects. We calculate the migrant coefficient with the main effect and the interaction. Analysis is conducted at the individual level. All variables are rescaled to vary from 0 to 1. Standard errors are clustered by the state where the respondent grew up. * p<0.05.

Table S31: Region Fixed Effects Survey Findings on the Persistence of Violent Victimization among White, Non-Hispanic Internal US Migrants within States of Residence. Analysis at the individual level with gender, five-year age group, and state-of-residence FEs. Robust standard errors clustered by state of birth. This is a pre-registered table and maintains the layout of our pre-registration rather than the layout of Table 3.

	Effect of Historical Homicide Rate		
Model	Coef.	SE	N
Do high hist. hom. birth state respondents see more violence growing up?			
Witness violence growing up (three-item scale)	0.042*	0.021	2,913
Do they see the world as more dangerous?			
Assault risk (three-item scale)	0.092*	0.027	2,502
Belief in a dangerous world (two-item scale)	0.108*	0.021	2,502
Do they disregard the law?			
Legal cynicism (three-item scale)	0.028	0.021	3,293
Do they report lower living standards?			
Living standard (two-item scale)	-0.025	0.024	3,293
Do high hist. hom. birth state respondents distrust institutions?			
Distrust local goverment where they grew up (one-item scale)	0.03	0.023	3,292
Distrust local goverment where they live now (one-item scale)	-0.012	0.024	3,292
Distrust police effectiveness and responsiveness where they live now (three-item scale)	0.017	0.017	3,293
(precision weighted average)	0.013	0.012	
Do high hist. hom. birth state respondents show adaptation to weak institutions?			
Trust family over the police (two-item scale)	0.067*	0.029	3,292
Distrust other people where they grew up (one-item scale)	0.101*	0.028	3,235
Distrust other people where they live now (one-item scale)	0.031	0.029	2,447
(precision weighted average)	0.066*	0.020	
Own gun mainly for protection	0.117*	0.036	3,291
Own gun partly for protection	0.004	0.035	3,291
Own gun not for protection	0.003	0.020	3,291
Aggressive self-response in the three threatening scenarios (three-item scale)	0.058	0.030	3,091
Aggressive response by friends where they grew up (three-item scale)	0.096*	0.029	3,091
Aggressive response by typical male/female where they grew up (three-item scale)	0.107*	0.034	3,091
(precision weighted average)	0.086*	0.018	
Aggressive response average in Kevin scenario (three-item scale)	0.077*	0.028	3,091
Aggressive response average in Emma scenario (three-item scale)	0.089*	0.033	2,898
Aggressive response average in Doug scenario (three-item scale)	0.099*	0.032	2,898
If Kevin and Doug walked away, they would look weak (two-item scale)	0.034	0.032	2,700
If Kevin and Doug walked away, they would not feel like men (two-item scale)	0.052	0.030	2,697
(precision weighted average)	0.044*	0.022	
Honor ideology in manhood (three-item scale)	0.113*	0.025	3,292
Raised by both parents	-0.127*	0.035	3,293

Note: Each set of coefficients and standard errors is from a separate regression. Standard errors are clustered by state of birth. * $p<0.05$.

Table S32: Pilot Survey Findings on the Persistence of Violent Victimization among White, Non-Hispanic Internal US Migrants and Non-Migrants. We collected these pilot samples before registration. Analysis at the individual level with gender and five-year age group fixed effects. This is a pre-registered table and maintains the layout of our pre-registration rather than the layout of Table 3.

	Effect of Historical Homicide Rate				
	Non-Mig.		Migrants		Persistence
Model	Coef.	SE	Coef.	SE	%
Do high hist. hom. birth state respondents see more violence growing up?					
Witness violence growing up (three-item scale)	0.076*	0.025	0.090*	0.043	119
Do they see the world as more dangerous?					
Assault risk (three-item scale)	0.086	0.050	0.121*	0.057	141
Belief in a Dangerous World (two-item scale)	0.011	0.044	0.187*	0.054	
Do they disregard the law?					
Legal Cynicism (three-item scale)	-0.033	0.022	0.007	0.029	-20
Do they report lower living standards?					
Living standard (two-item scale)	0.006	0.031	-0.026	0.038	
Do high hist. hom. birth state respondents distrust institutions?					
Distrust local goverment where they grew up (one-item scale)	0.059	0.035	0.027	0.036	45
Distrust local goverment where they live now (one-item scale)	0.036	0.037	0.008	0.034	22
Distrust police effectiveness and responsiveness where they live now (three-item scale)	0.048	0.028	0.013	0.031	27
(precision weighted average)	0.048*	0.019	0.015	0.019	32
Do high hist. hom. birth state respondents show adaptation to weak institutions?					
Trust family over the police (two-item scale)	0.083*	0.029	0.075	0.048	90
Distrust other people where they grew up (one-item scale)	0.05	0.029	0.088*	0.039	174
Distrust other people where they live now (one-item scale)	-0.062	0.052	-0.022	0.063	36
(precision weighted average)	0.024	0.025	0.057	0.033	
Own gun mainly for protection	0.272*	0.044	0.148*	0.055	55
Own gun partly for protection	0.046	0.046	0.046	0.054	98
Own gun not for protection	-0.093*	0.020	-0.011	0.028	12
Aggressive self-response in the three threatening scenarios (three-item scale)	0.131*	0.034	0.088*	0.037	67
Aggressive response by friends where they grew up (three-item scale)	0.131*	0.034	0.142*	0.051	109
Aggressive response by typical male/female where they grew up (three-item scale)	0.099*	0.032	0.155*	0.050	158
(precision weighted average)	0.119*	0.019	0.120*	0.026	100
Aggressive response average in Kevin scenario (three-item scale)	0.120*	0.030	0.140*	0.045	117
Aggressive response average in Emma scenario (three-item scale)	0.104*	0.038	0.120*	0.057	115
Aggressive response average in Doug scenario (three-item scale)	0.095*	0.048	0.154*	0.047	163
If Kevin and Doug walked away, they would look weak (two-item scale)	0.117*	0.037	0.059	0.054	50
If Kevin and Doug walked away, they would not feel like men (two-item scale)	0.054	0.039	0.075	0.056	138
(precision weighted average)	0.087*	0.027	0.067	0.039	76
Honor ideology in manhood (three-item scale)	0.095*	0.023	0.108*	0.049	113
Hotheadedness (three-item scale)	0.065*	0.022	0.033	0.032	51
Raised by both parents	-0.128*	0.040	-0.177*	0.054	139

Note: Each set of coefficients and standard errors is from a separate regression. Standard errors are clustered by state of birth. * p<0.05.

Table S33: Region Fixed Effects Pilot Sample Survey Findings on the Persistence of Violent Victimization among White, Non-Hispanic Internal US Migrants within States of Residence. We collected these pilot samples before registration. Analysis at the individual level with gender, five-year age group, and state-of-residence FEs. Robust standard errors clustered by state of birth. This is a pre-registered table and maintains the layout of our pre-registration rather than the layout of Table 3.

	Effect of Historical Homicide Rate		
Model	Coef.	SE	N
Do high hist. hom. birth state respondents see more violence growing up?			
Witness violence growing up (three-item scale)	0.099*	0.043	780
Do they see the world as more dangerous?			
Assault risk (three-item scale)	0.114*	0.054	369
Belief in a dangerous world (two-item scale)	0.193*	0.054	369
Do they disregard the law?			
Legal cynicism (three-item scale)	0.017	0.030	1,160
Do they report lower living standards?			
Living standard (two-item scale)	-0.03	0.039	1,160
Do high hist. hom. birth state respondents distrust institutions?			
Distrust local goverment where they grew up (one-item scale)	0.027	0.034	1,160
Distrust local goverment where they live now (one-item scale)	-0.003	0.037	1,160
Distrust police effectiveness and responsiveness where they live now (three-item scale)	0.002	0.033	1,160
(precision weighted average)	0.009	0.020	
Do high hist. hom. birth state respondents show adaptation to weak institutions?			
Trust family over the police (two-item scale)	0.051	0.057	1,160
Distrust other people where they grew up (one-item scale)	0.102*	0.045	1,140
Distrust other people where they live now (one-item scale)	-0.045	0.076	365
(precision weighted average)	0.064	0.039	
Own gun mainly for protection	0.083	0.071	1,159
Own gun partly for protection	0.013	0.054	1,159
Own gun not for protection	-0.015	0.028	1,159
Aggressive self-response in the three threatening scenarios (three-item scale)	0.083*	0.039	958
Aggressive response by friends where they grew up (three-item scale)	0.159*	0.052	958
Aggressive response by typical male/female where they grew up (three-item scale)	0.163*	0.053	958
(precision weighted average)	0.124*	0.027	
Aggressive response average in Kevin scenario (three-item scale)	0.140*	0.051	958
Aggressive response average in Emma scenario (three-item scale)	0.139*	0.055	765
Aggressive response average in Doug scenario (three-item scale)	0.169*	0.053	765
If Kevin and Doug walked away, they would look weak (two-item scale)	0.071	0.062	571
If Kevin and Doug walked away, they would not feel like men (two-item scale)	0.130*	0.060	569
(precision weighted average)	0.101*	0.043	
Honor ideology in manhood (three-item scale)	0.123*	0.042	1,159
Hot headedness (three-item scale)	0.035	0.033	1,160

Note: Each set of coefficients and standard errors is from a separate regression. Standard errors are clustered by state of birth. * p<0.05.

Table S34: Final Sample Survey Findings on the Persistence of Violent Victimization among White, Non-Hispanic Internal US Migrants and Non-Migrants. Pilot respondents excluded. Analysis at the individual level with for gender and five-year age group fixed effects. This is a pre-registered table and maintains the layout of our pre-registration rather than the layout of Table 3.

	Effect of Historical Homicide Rate				
	Non-Mig.		Migrants		Persistence
Model	Coef.	SE	Coef.	SE	%
Do high hist. hom. birth state respondents see more violence growing up?					
Witness violence growing up (three-item scale)	0.043	0.023	0.019	0.020	43
Do they see the world as more dangerous?					
Assault risk (three-item scale)	0.058*	0.023	0.081*	0.026	140
Belief in a Dangerous World (two-item scale)	0.088*	0.016	0.102*	0.022	116
Do they disregard the law?					
Legal Cynicism (three-item scale)	-0.018	0.023	0.019	0.025	
Do they report lower living standards?					
Living standard (two-item scale)	-0.026	0.022	-0.019	0.025	
Do high hist. hom. birth state respondents distrust institutions?					
Distrust local goverment where they grew up (one-item scale)	0.006	0.030	0.057*	0.025	
Distrust local goverment where they live now (one-item scale)	0.042	0.033	0.008	0.023	20
Distrust police effectiveness and responsiveness where they live now (three-item scale)	0.081*	0.026	0.046*	0.020	56
(precision weighted average)	0.047*	0.017	0.037*	0.013	79
Do high hist. hom. birth state respondents show adaptation to weak institutions?					
Trust family over the police (two-item scale)	0.059*	0.028	0.079*	0.024	134
Distrust other people where they grew up (one-item scale)	0.074*	0.030	0.104*	0.033	140
Distrust other people where they live now (one-item scale)	0.059	0.033	0.048	0.027	82
(precision weighted average)	0.067*	0.022	0.071*	0.021	105
Own gun mainly for protection	0.292*	0.052	0.164*	0.051	56
Own gun partly for protection	0.042	0.046	0.018	0.039	45
Own gun not for protection	-0.070*	0.025	-0.002	0.028	3
Aggressive self-response in the three threatening scenarios (three-item scale)	0.082*	0.033	0.048	0.030	59
Aggressive response by friends where they grew up (three-item scale)	0.101*	0.026	0.066*	0.032	65
Aggressive response by typical male/female where they grew up (three-item scale)	0.089*	0.027	0.084*	0.033	95
(precision weighted average)	0.092*	0.016	0.065*	0.018	71
Aggressive response average in Kevin scenario (three-item scale)	0.106*	0.030	0.05	0.027	47
Aggressive response average in Emma scenario (three-item scale)	0.094*	0.032	0.074*	0.036	79
Aggressive response average in Doug scenario (three-item scale)	0.072*	0.029	0.074*	0.030	103
If Kevin and Doug walked away, they would look weak (two-item scale)	0.021	0.032	0.012	0.029	
If Kevin and Doug walked away, they would not feel like men (two-item scale)	0.054*	0.025	0.033	0.025	61
(precision weighted average)	0.041*	0.020	0.024	0.019	58
Honor ideology in manhood (three-item scale)	0.099*	0.035	0.105*	0.024	107
Hotheadedness (three-item scale)	0.03	0.022	0.02	0.020	66
Raised by both parents	-0.058	0.039	-0.112*	0.048	193

Note: Each set of coefficients and standard errors is from a separate regression. Standard errors are clustered by state of birth. * p<0.05.

Table S35: Final Sample Region Fixed Effects Survey Findings on the Persistence of Violent Victimization among White, Non-Hispanic Internal US Migrants within States of Residence. We collected these pilot samples before registration. Analysis at the individual level with gender, five-year age group, and state-of-residence FEs. Robust standard errors clustered by state of birth. This is a pre-registered table and maintains the layout of our pre-registration rather than the layout of Table 3.

	Effect of Historical Homicide Rate		
Model	Coef.	SE	N
Do high hist. hom. birth state respondents see more violence growing up?			
Witness violence growing up (three-item scale)	0.021	0.020	2,133
Do they see the world as more dangerous?			
Assault risk (three-item scale)	0.081*	0.026	2,133
Belief in a dangerous world (two-item scale)	0.102*	0.022	2,133
Do they disregard the law?			
Legal cynicism (three-item scale)	0.033	0.023	2,133
Do they report lower living standards?			
Living standard (two-item scale)	-0.016	0.024	2,133
Do high hist. hom. birth state respondents distrust institutions?			
Distrust local goverment where they grew up (one-item scale)	0.038	0.028	2,132
Distrust local goverment where they live now (one-item scale)	-0.01	0.023	2,132
Distrust police effectiveness and responsiveness where they live now (three-item scale)	0.033	0.021	2,133
(precision weighted average)	0.019	0.014	
Do high hist. hom. birth state respondents show adaptation to weak institutions?			
Trust family over the police (two-item scale)	0.075*	0.026	2,132
Distrust other people where they grew up (one-item scale)	0.104*	0.034	2,095
Distrust other people where they live now (one-item scale)	0.042	0.031	2,082
(precision weighted average)	0.070*	0.023	
Own gun mainly for protection	0.133*	0.049	2,132
Own gun partly for protection	-0.014	0.040	2,132
Own gun not for protection	0.012	0.026	2,132
Aggressive self-response in the three threatening scenarios (three-item scale)	0.043	0.031	2,133
Aggressive response by friends where they grew up (three-item scale)	0.071*	0.034	2,133
Aggressive response by typical male/female where they grew up (three-item scale)	0.083*	0.036	2,133
(precision weighted average)	0.064*	0.020	
Aggressive response average in Kevin scenario (three-item scale)	0.046	0.030	2,133
Aggressive response average in Emma scenario (three-item scale)	0.075*	0.037	2,133
Aggressive response average in Doug scenario (three-item scale)	0.076*	0.033	2,133
If Kevin and Doug walked away, they would look weak (two-item scale)	0.024	0.035	2,129
If Kevin and Doug walked away, they would not feel like men (two-item scale)	0.037	0.032	2,128
(precision weighted average)	0.031	0.024	
Honor ideology in manhood (three-item scale)	0.110*	0.024	2,133
Hot headedness (three-item scale)	0.016	0.020	2,133

Note: Each set of coefficients and standard errors is from a separate regression. Standard errors are clustered by state of birth. * p<0.05.

Table S36: Post Updated Registration Survey Findings on the Persistence of Violent Victimization among White, Non-Hispanic Internal US Migrants and Non-Migrants. Analysis at the individual level with gender and five-year age group fixed effects. This is a pre-registered table and maintains the layout of our pre-registration rather than the layout of Table 3.

	Effect of Historical Homicide Rate				
	Non-Mig.		Migrants		Persistence
Model	Coef.	SE	Coef.	SE	%
Do high hist. hom. birth state respondents see more violence growing up?					
Witness violence growing up (three-item scale)	0.059	0.040	0.03	0.035	50
Do they see the world as more dangerous?					
Assault risk (three-item scale)	0.081*	0.035	0.072*	0.034	89
Belief in a Dangerous World (two-item scale)	0.108*	0.024	0.093*	0.035	86
Do they disregard the law?					
Legal Cynicism (three-item scale)	-0.061*	0.031	-0.023	0.034	38
Do they report lower living standards?					
Living standard (two-item scale)	-0.055	0.036	-0.075*	0.035	137
Do high hist. hom. birth state respondents distrust institutions?					
Distrust local goverment where they grew up (one-item scale)	-0.026	0.037	0.034	0.040	
Distrust local goverment where they live now (one-item scale)	0.037	0.036	0.048	0.034	132
Distrust police effectiveness and responsiveness where they live now (three-item scale)	0.098*	0.029	0.051	0.040	51
(precision weighted average)	0.047*	0.019	0.045*	0.022	96
Do high hist. hom. birth state respondents show adaptation to weak institutions?					
Trust family over the police (two-item scale)	0.034	0.036	0.061	0.038	179
Distrust other people where they grew up (one-item scale)	0.037	0.043	0.083*	0.041	223
Distrust other people where they live now (one-item scale)	0.058	0.036	0.110*	0.037	191
(precision weighted average)	0.049	0.028	0.098*	0.028	198
Own gun mainly for protection	0.252*	0.052	0.170*	0.081	67
Own gun partly for protection	0.064	0.075	0.08	0.047	125
Own gun not for protection	-0.073*	0.030	-0.032	0.036	43
Aggressive self-response in the three threatening scenarios (three-item scale)	0.089*	0.043	0.042	0.046	47
Aggressive response by friends where they grew up (three-item scale)	0.131*	0.041	0.067	0.043	51
Aggressive response by typical male/female where they grew up (three-item scale)	0.105*	0.038	0.065	0.037	62
(precision weighted average)	0.108*	0.024	0.059*	0.024	55
Aggressive response average in Kevin scenario (three-item scale)	0.118*	0.050	0.032	0.047	27
Aggressive response average in Emma scenario (three-item scale)	0.107*	0.041	0.087	0.045	81
Aggressive response average in Doug scenario (three-item scale)	0.098*	0.040	0.055	0.039	56
If Kevin and Doug walked away, they would look weak (two-item scale)	0.077	0.046	0.033	0.040	43
If Kevin and Doug walked away, they would not feel like men (two-item scale)	0.059	0.042	0.05	0.044	85
(precision weighted average)	0.067*	0.031	0.041	0.029	61
Honor ideology in manhood (three-item scale)	0.112*	0.040	0.144*	0.038	129
Hotheadedness (three-item scale)	0.025	0.027	-0.003	0.033	
Raised by both parents	-0.114	0.065	-0.128*	0.063	112

Note: Each set of coefficients and standard errors is from a separate regression. Standard errors are clustered by state of birth. * p<0.05.

S2 Supporting Information Figures

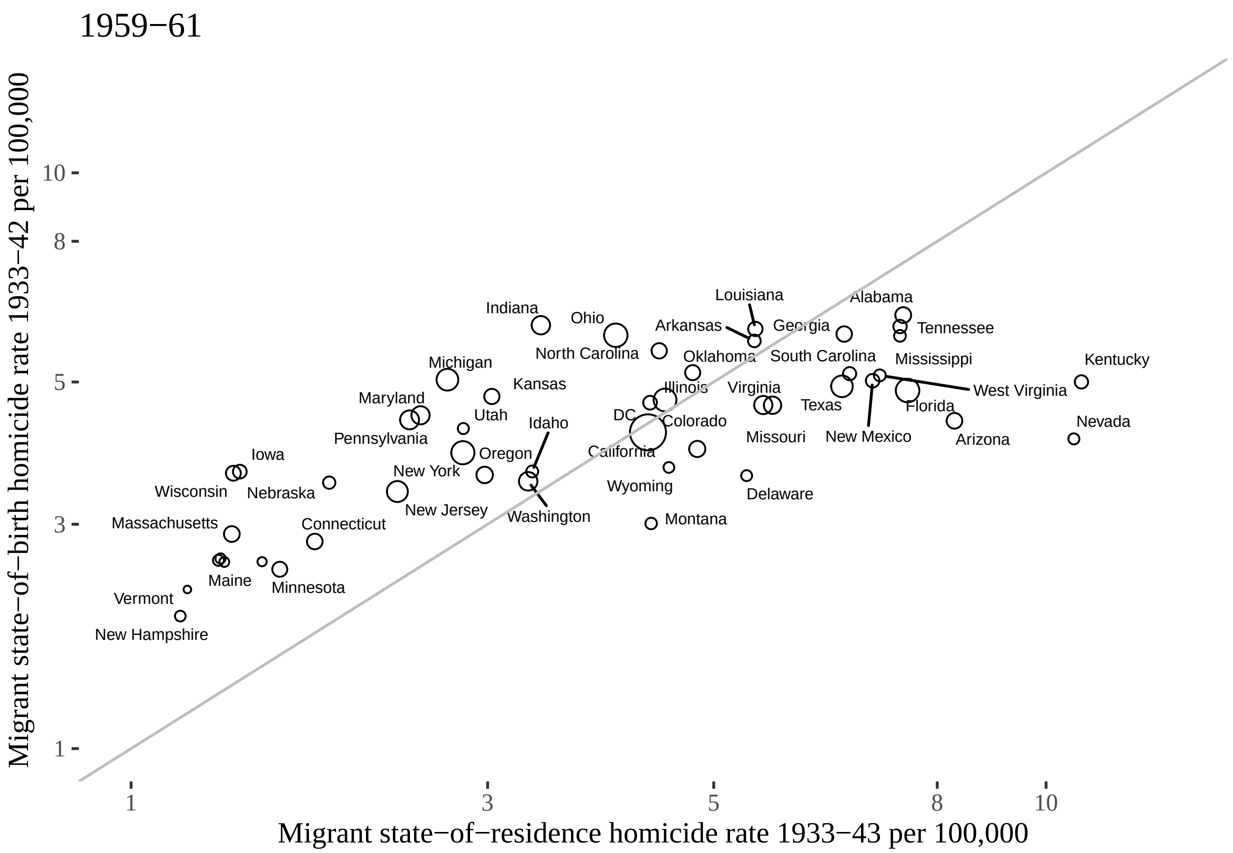

Figure S1: This figure examines the degree to which historically violent states for whites attracted white migrants from historically violent states. Each circle shows the average historical homicide rate for migrants (weighted by population). Each circle is sized by the white migrant population. The figure shows less selection than one might expect, with more violent states disproportionately receiving migrants from less violent states, and less violent states disproportionately receiving migrants from more violent states. The gray line shows the 45 degree line.

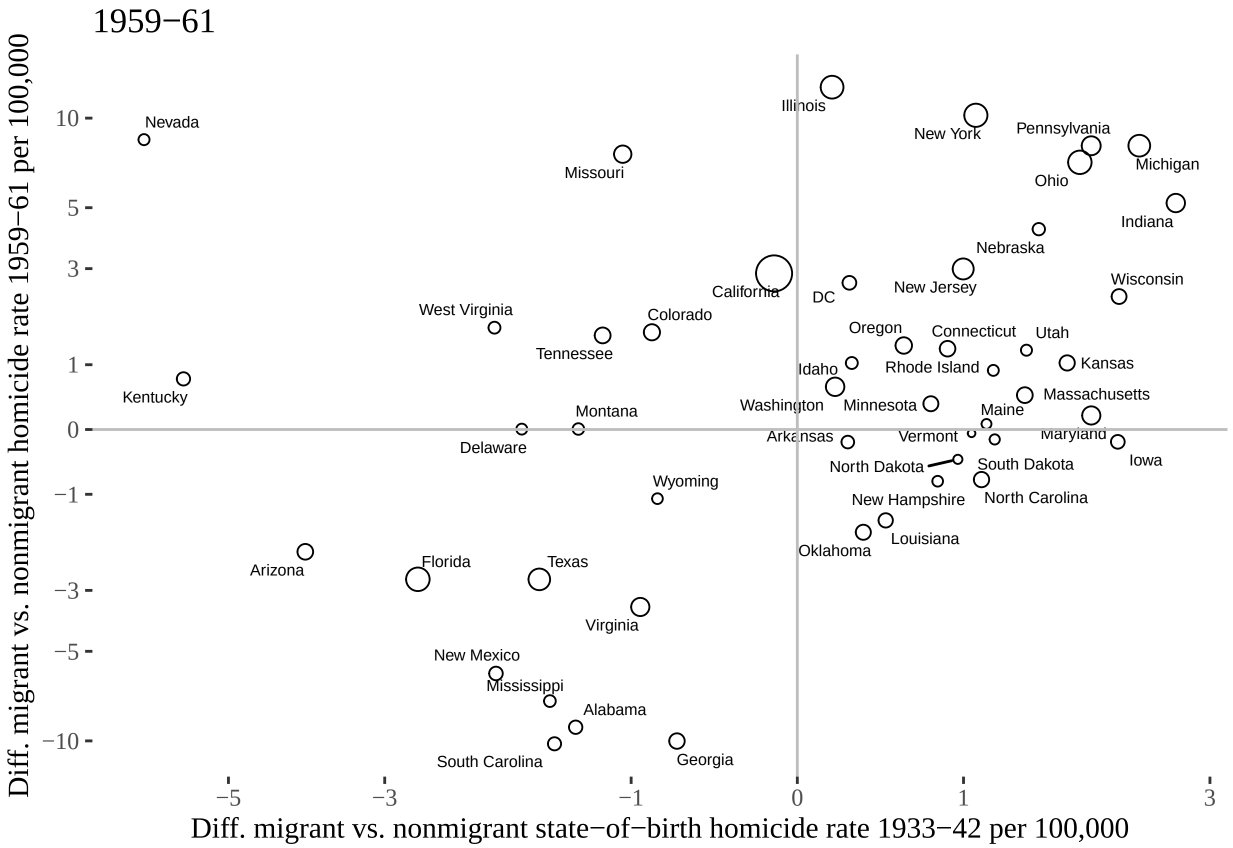

Figure S2: This figure shows which states received white migrants from relatively more historically violent states than non-migrants' historical violence rates (x-axis) and whether these migrants were relatively more at risk from violence in their new state than non-migrants (y-axis). It does so by taking the difference between the historical homicide rates of migrants and nonmigrants in each state of residence as the x axis, and this same difference for 1959-61 homicide rates as the y axis. States in the upper right quadrant received migrants from historically more violent states and those migrants were more at risk of violence in the states than locals. Indiana, for instance, had migrants with an average historical homicide rate three points higer than Indiana's historical homicide rate, the largest of any state. It also had migrants more at risk than nonmigrants. Nevada is an outlier because it had a trivial nonmigrant population in this period. Another outlier, Kentucky, received migrants from historically less violent states (relatively speaking), but those migrants died at higher than expected rates. Circles sized by the white migrant population.

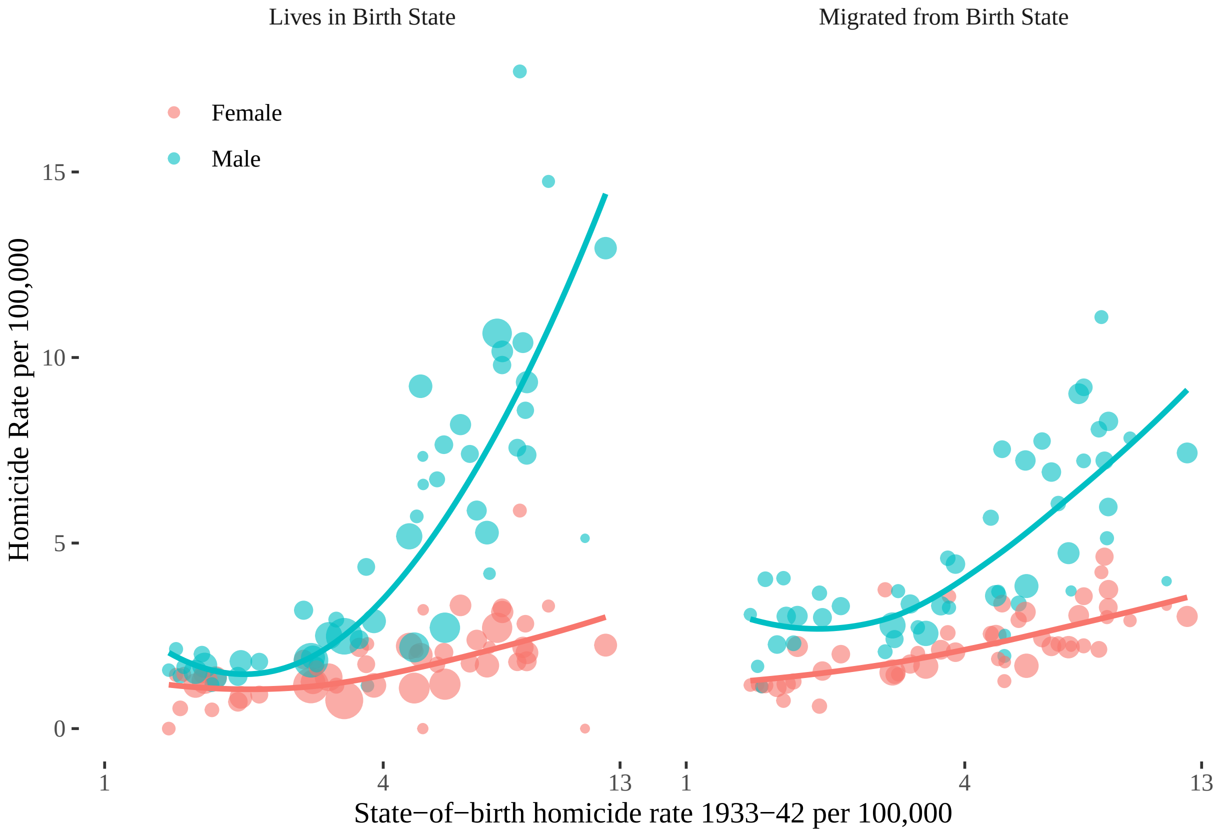

Figure S3: 1959-61 homicide rates by gender for white migrants and non-migrants. Each circle shows a state of birth. For migrants, each shows the homicide rate averaged across wherever migrants from a state ended up. Circles sized by the white non-migrant or migrant population. Loess smoother. The smaller number of person years and homicides yields noisier estimates when disaggregating by sex. Note the unlogged y-axis.

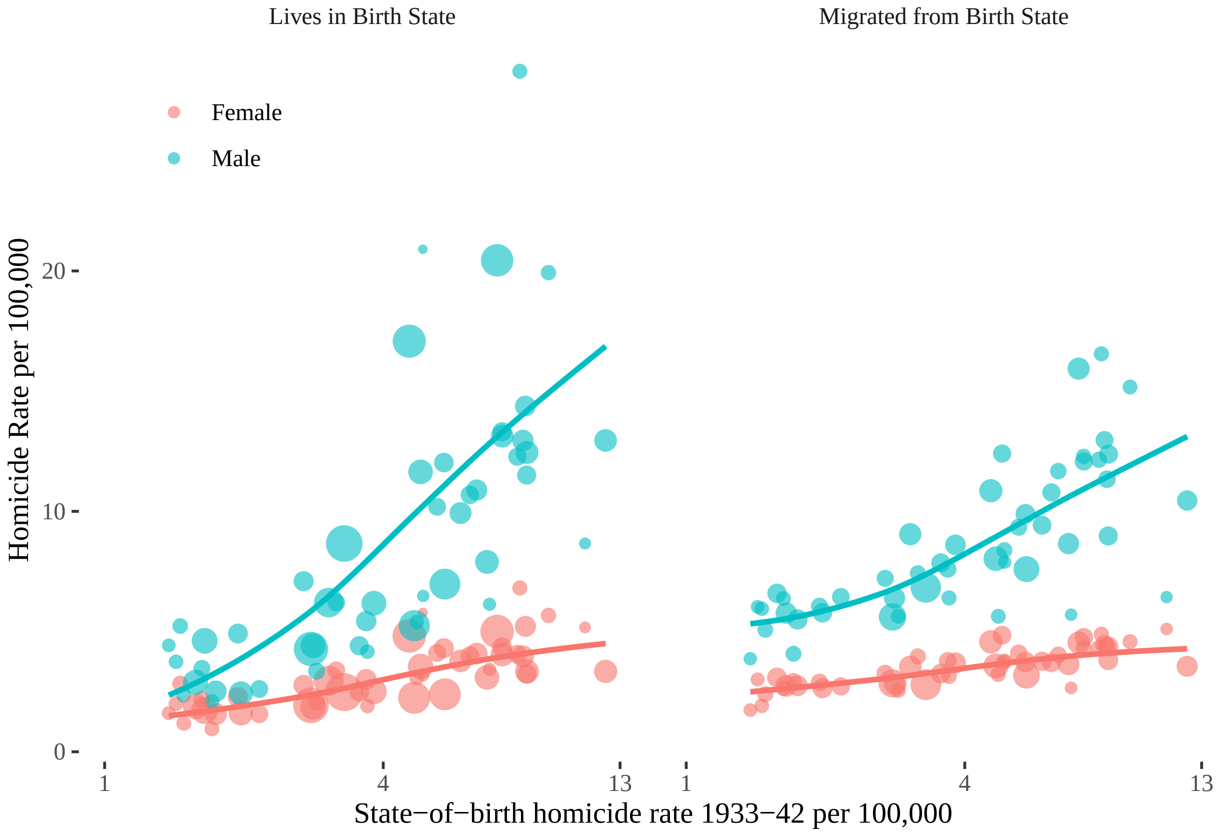

Figure S4: 1979-91 homicide rates by gender for migrants and non-migrants. Each circle shows a state of birth. For migrants, each shows the homicide rate averaged across wherever migrants from a state ended up. Circles sized by the white non-migrant or migrant population. Loess smoother. Note the unlogged y-axis.

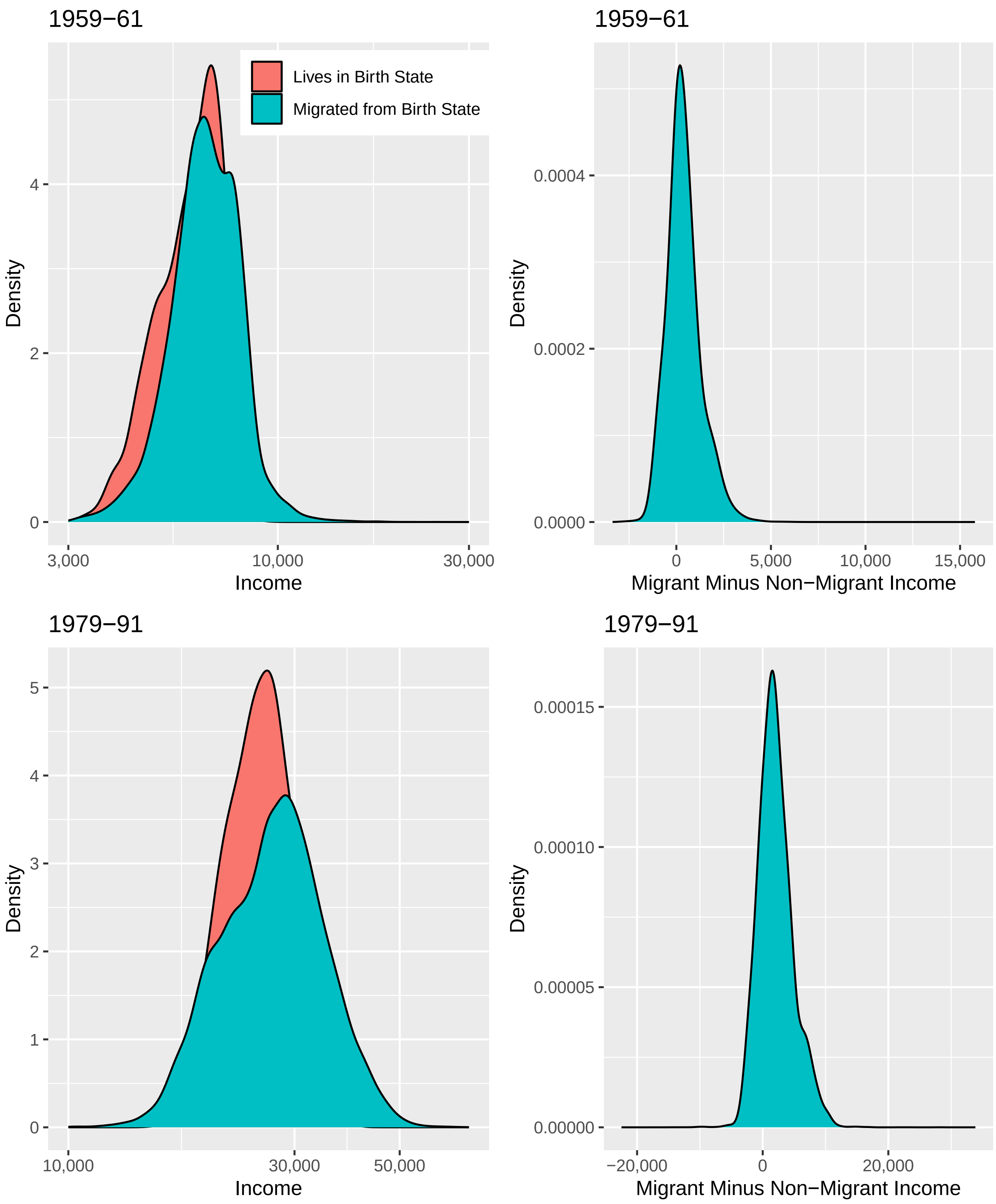

Figure S5: This figure plots the distribution of migrant and non-migrant incomes and the difference between those incomes. It shows that migrants were often better off than their new neighbors. For non-migrants, each row of the data is a state-of-residence age group, e.g., 50-54 year-old Kentuckians. For migrants, each row of data is migrants from a particular state of birth in their current state of residence for an age group, e.g., 50-54 Kentuckians living in Illinois. The distributions are weighted by the population size of each respective group.

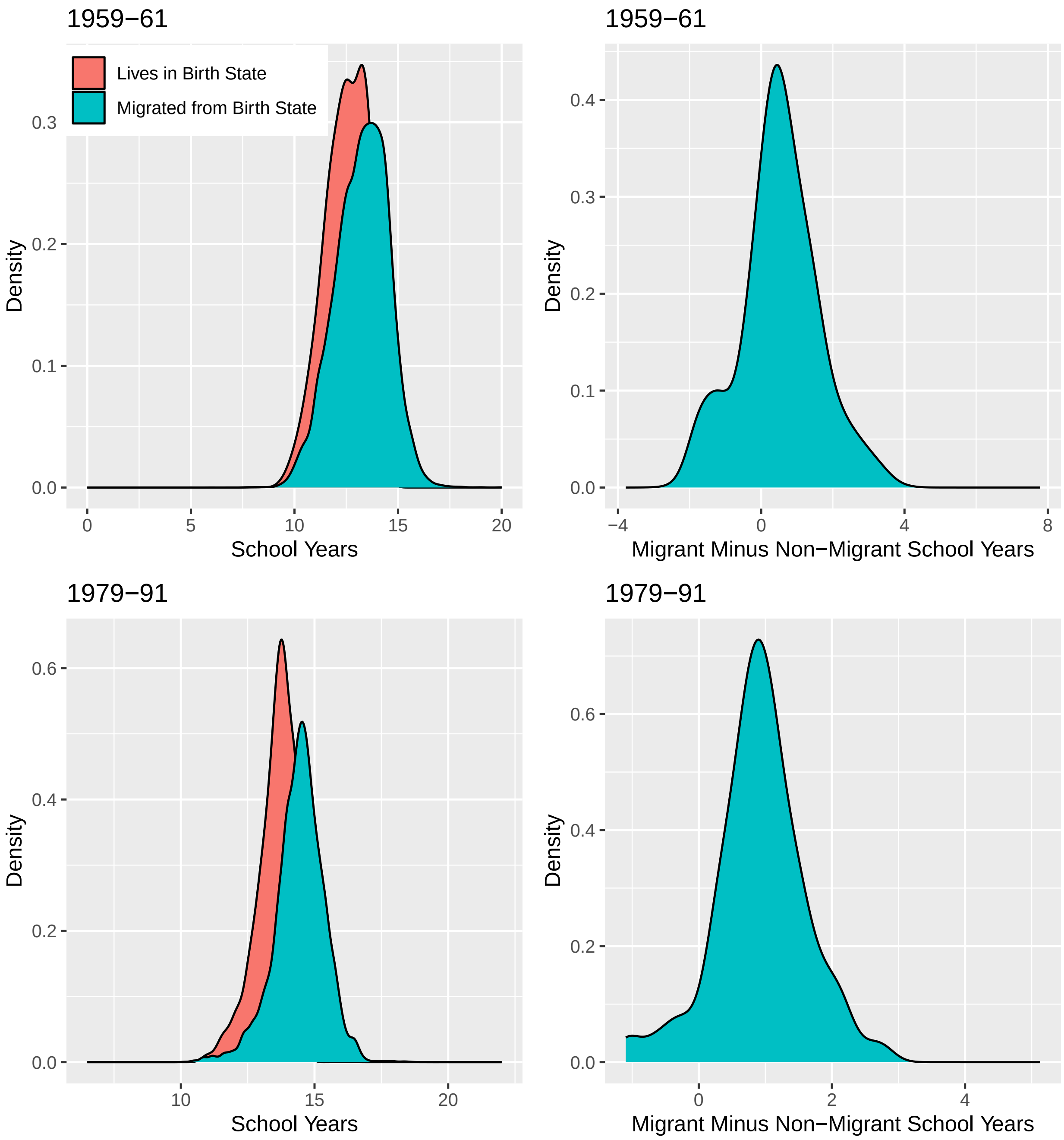

Figure S6: This figure plots the distribution of migrant and non-migrant years in school and the difference between them in years in school for migrant groups 25 and over. It shows that migrants were often better educated than their new neighbors. For non-migrants, each row of the data is a state-of-residence age group, e.g., 50-54 year-old Kentuckians. For migrants, each row of data is migrants from a particular state of birth in their current state of residence for an age group, e.g., 50-54 Kentuckians living in Illinois. The distributions are weighted by the population size of each respective group.

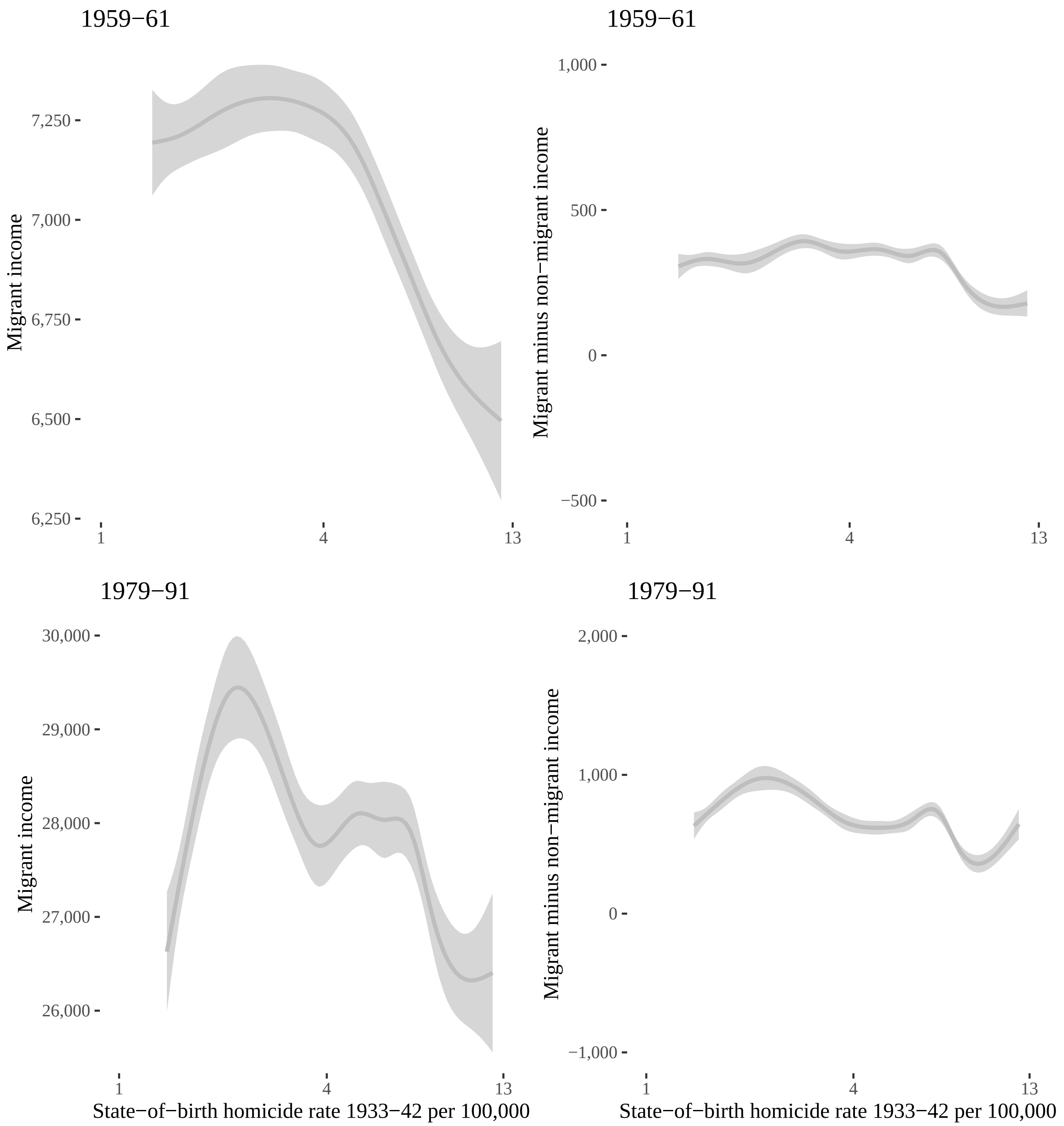

Figure S7: This figure plots the relationship between historical homicide rates and migrant incomes (left) and the difference between their incomes and those of their new neighbors (right). Although migrants are generally better off than non-migrants, the left panels in this figure show that migrants from the most historically violent states are somewhat worse off financially. However, compared to the residents where they migrate to, the right panels show that these migrants are not worse off. So we would not expect lower incomes relative to incomes where they've migrated to, to contribute to persistence of violence. Each row of data is migrants from a particular state of birth in their current state of residence for an age group, e.g., 50-54 Kentuckians living in Illinois. Loess smoother. The data are weighted by the population size of each respective group.

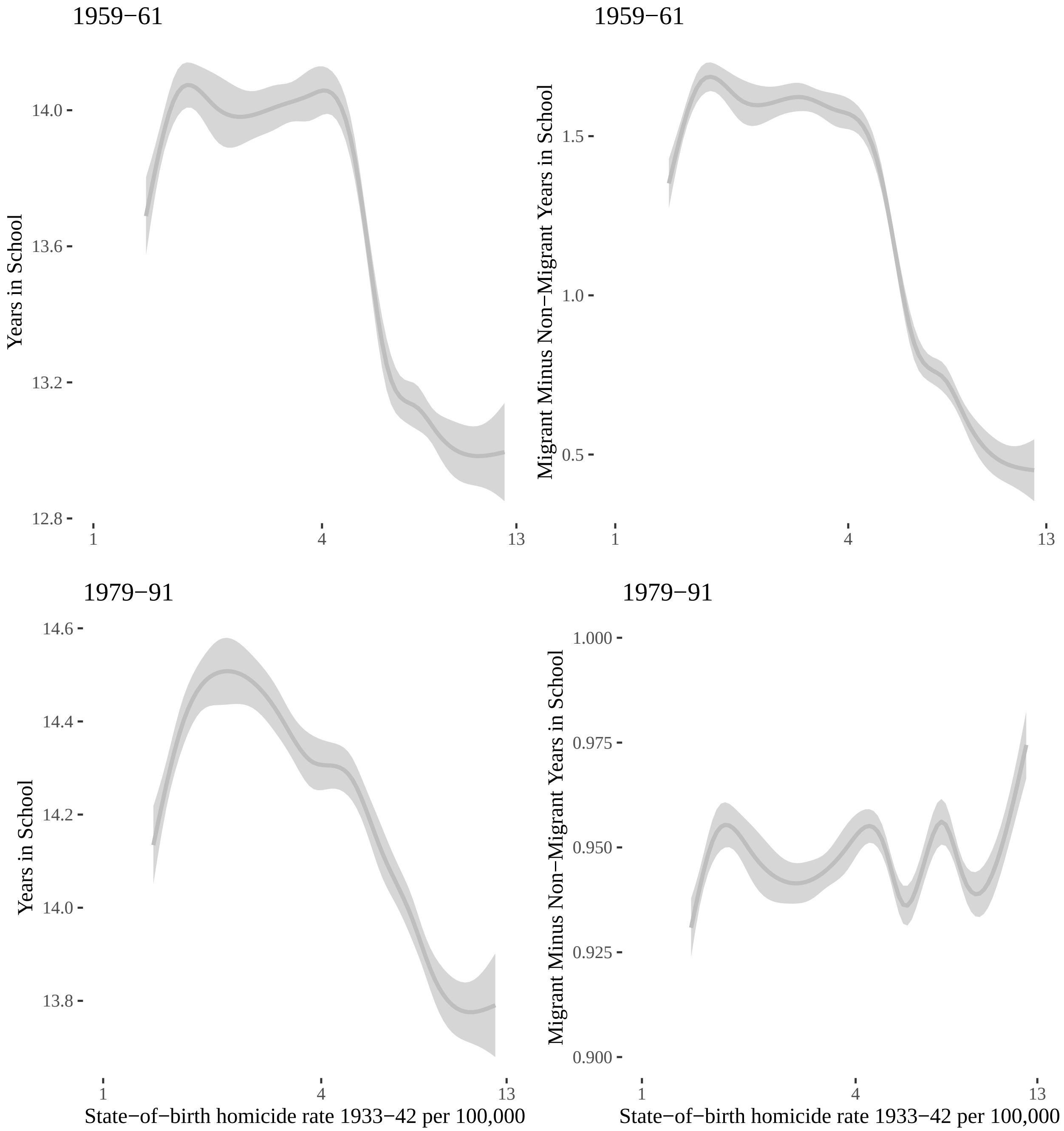

Figure S8: This figure plots the relationship between historical homicide rates and migrant years in scool (left) and the difference between their years in school and those of their new neighbors (right). Although migrants are generally better educated than non-migrants, the left panels in this figure show that migrants from the most historically violent states are somewhat less educated. However, compared to the residents where they migrate to, the right panels show that these migrants are only trivially less educated. So we would not expect lower education relative to those living where they have migrated to to contribute to persistence of violence. Each row of data is migrants from a particular state of birth in their current state of residence for an age group, e.g., 50-54 Kentuckians living in Illinois. Loess smoother. The data are weighted by the population size of each respective group.

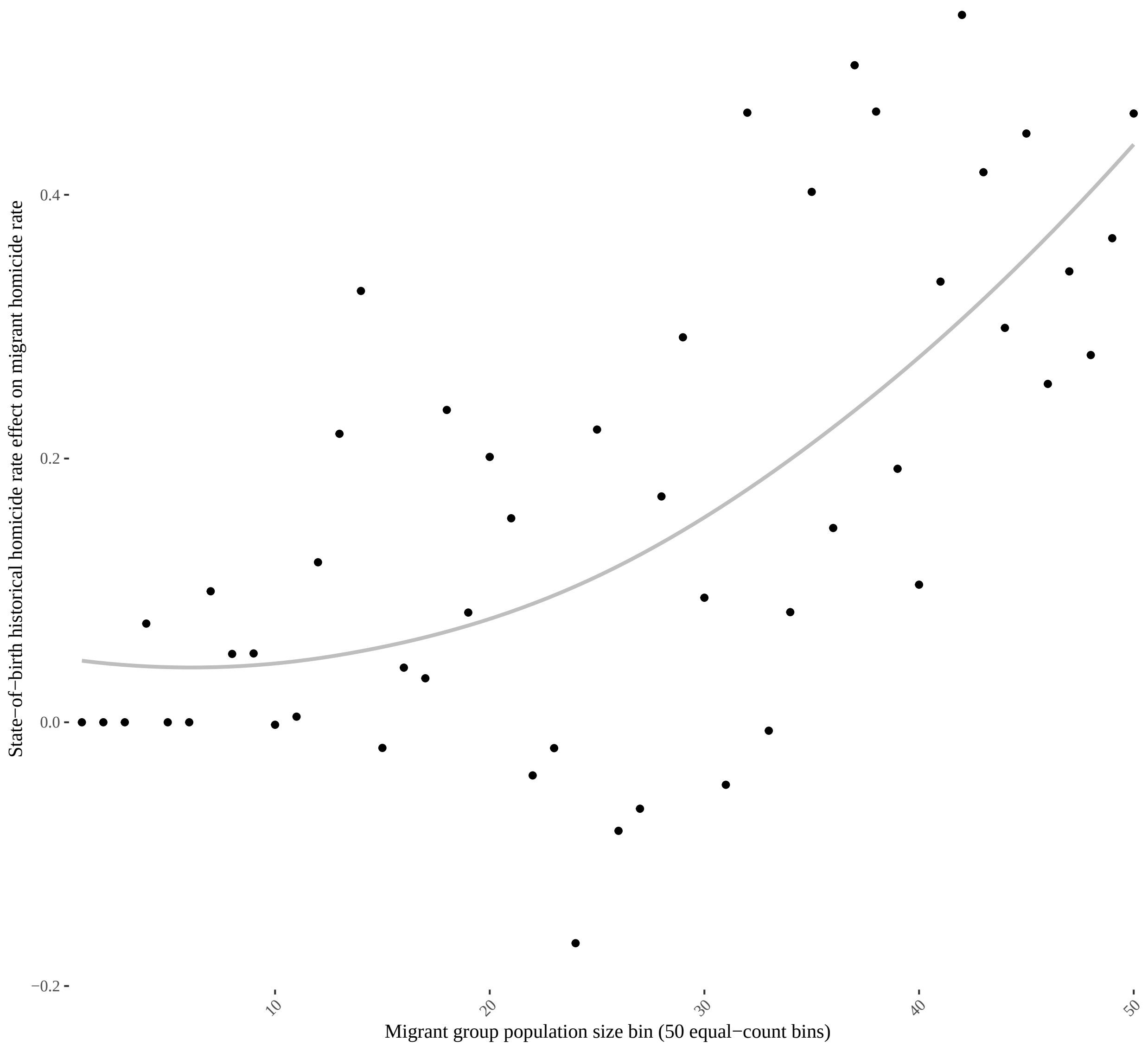

Figure S9: Persistence estimates for 50 migrant group population bins, 1979-91. Each point shows an estimate for migrant groups of a particular population size range (bin) from a regression at the state-of-birth-county level with county fixed effects weighted by migrant populations. Each point/bin contains about 200 migrant groups, e.g., Kentucky-born migrants living in San Francisco. We present the 1979-91 figure because we have lots of homicides (dangerous time) and a large population during this period. The increase shows no sign of plateauing, which seems more consistent with migrant population size facilitating persistence. If migrant population size mattered only because larger groups give us more precise estimates of homicide rates, we would expect the increase in effect size to level off as group size grows—since each additional observation contributes less to reducing measurement error. Unfortunately, we lack the power to tell for sure if it's non-plateuing. We find similar patterns when we omit county fixed effects and when we use other numbers of bins. We observe a more consistently positive effect above about the 30th bin, which corresponds to migrant populations of about 4,000. The first migrant population bin has migrant group sizes of around 20. The 49th and 50th are 75,010-127,010 and 127,010-2,085,850, respectively.

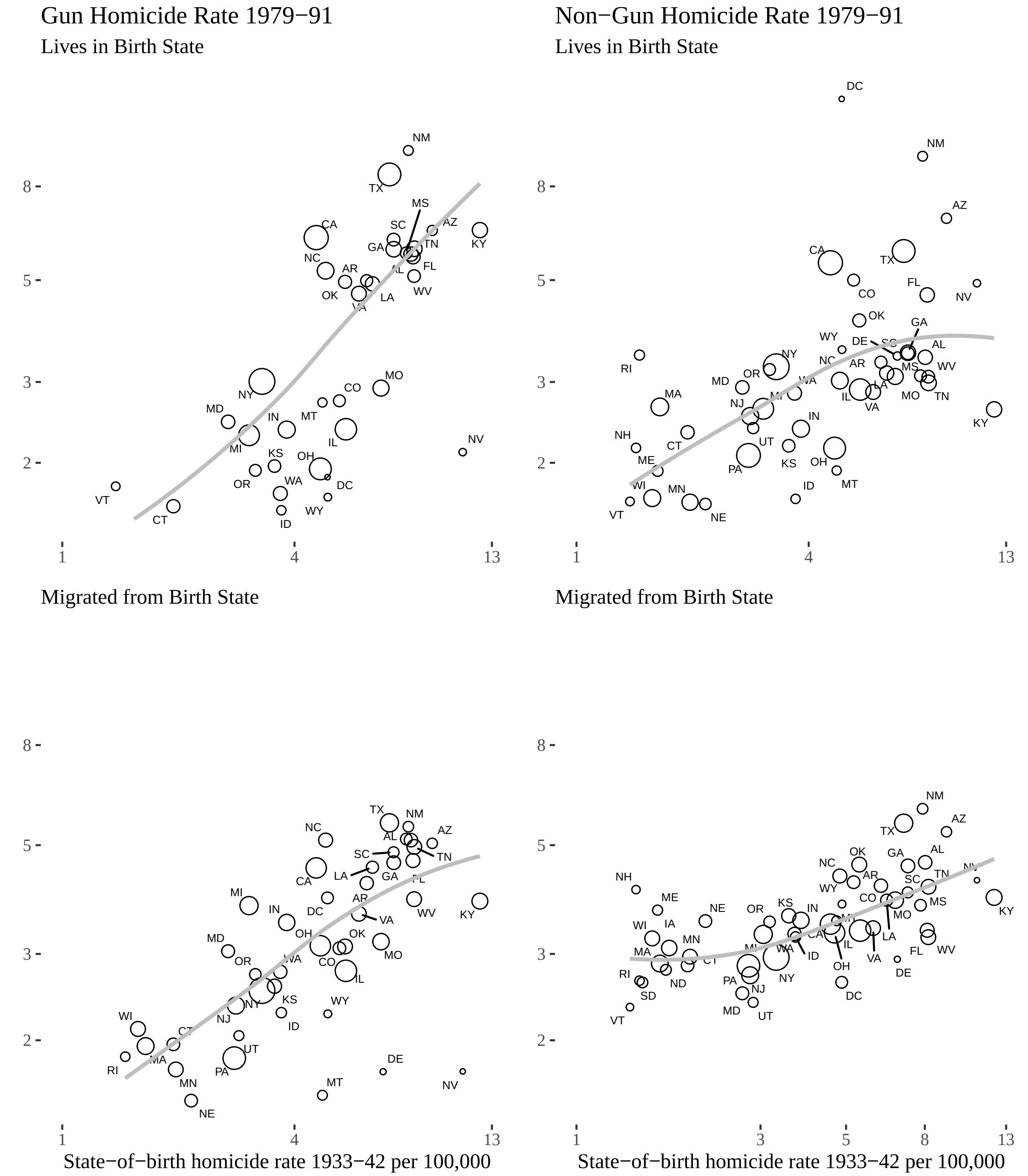

Figure S10: US Gun Homicide (left) and Non-Gun Homicide (right) Victimization Rate 1979-91 by Historical State of Birth Homicide Victimization Rate for Whites Ages 15-60 by Migration Status. For migrants, each point shows the homicide rate averaged across wherever those migrants ended up. Loess lines are weighted and circles are sized by white population. We calculated these for 1979-91 because it provides the largest number of homicides. In log-log regressions, the non-gun coefficients for the historical homicide rate are 0.44 for non-migrants and 0.24 for migrants, for a persistence coefficient just above 0.5, similar to the persistence coefficients presented in Table 1.

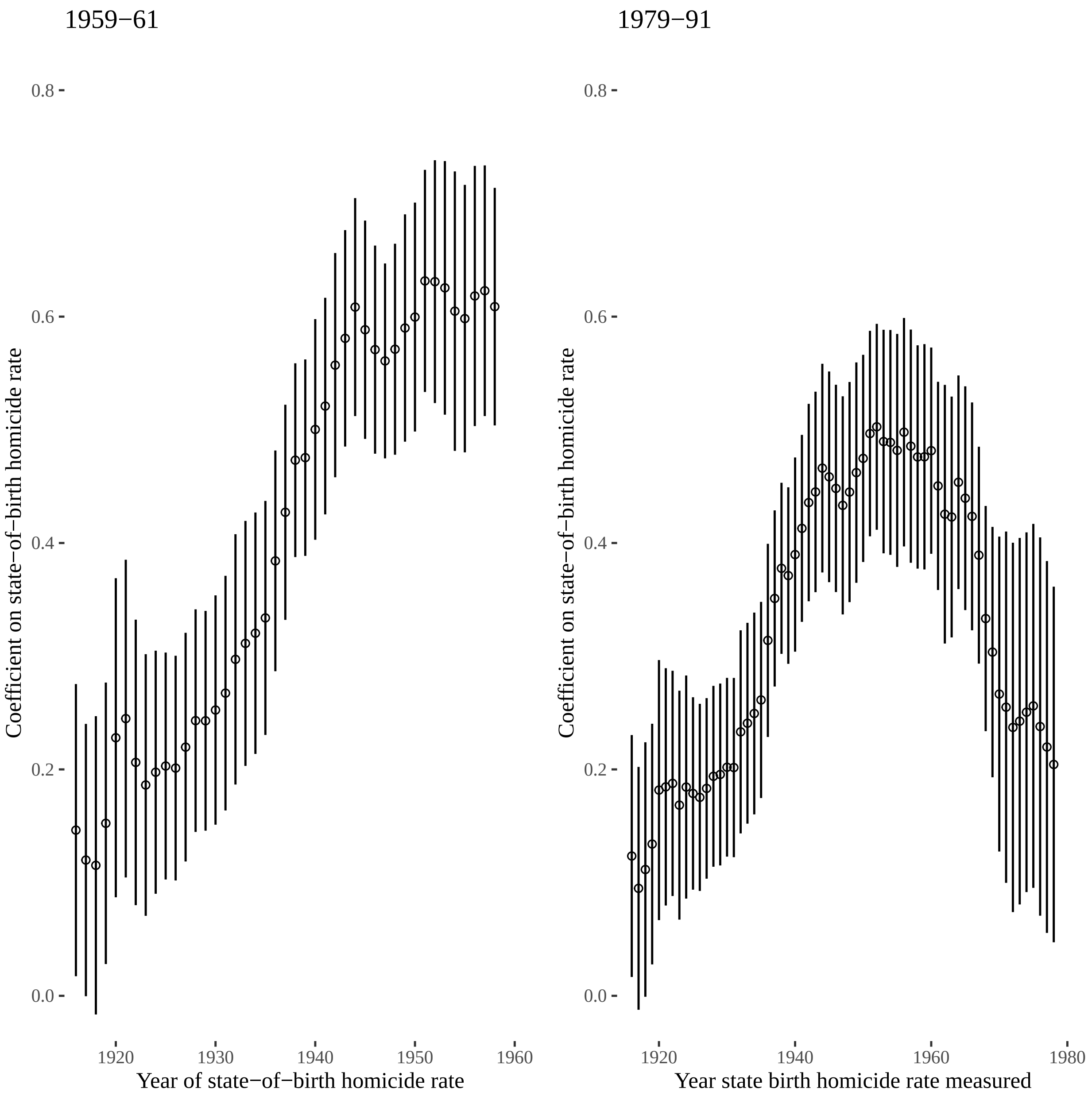

Figure S11: This figure shows how our estimates of persistence in homicide rates for migrants change with the years we use to measure the historical homicide rate in states of birth. In the main results in the paper, we measure the historical homicide rate with the 1933-42 average. In this figure, we replace that measure with the three-year moving average. Each point shows the estimate of persistence for homicides for each of these three year averages (before 1933, we only have homicide rates for a subset of states so the estimates aren't comparable pre-1933). For 1959-61, the left panel shows that measuring the homicide rate closer to 1959-61 improves predictions of migrants' homicide rates. For 1979-91, the right panel shows a rise and decline, which is partially driven by New York and especially New York City becoming more dangerous for whites in the 1970s, while white migrants from New York state remained quite safe. The figure on the next page shows the 1979-91 estimates without New York State. The estimates here use our main specification from Table 2 with state times age group fixed effects. 95% confidence intervals shown calculated from robust standard errors clustered by state of birth.

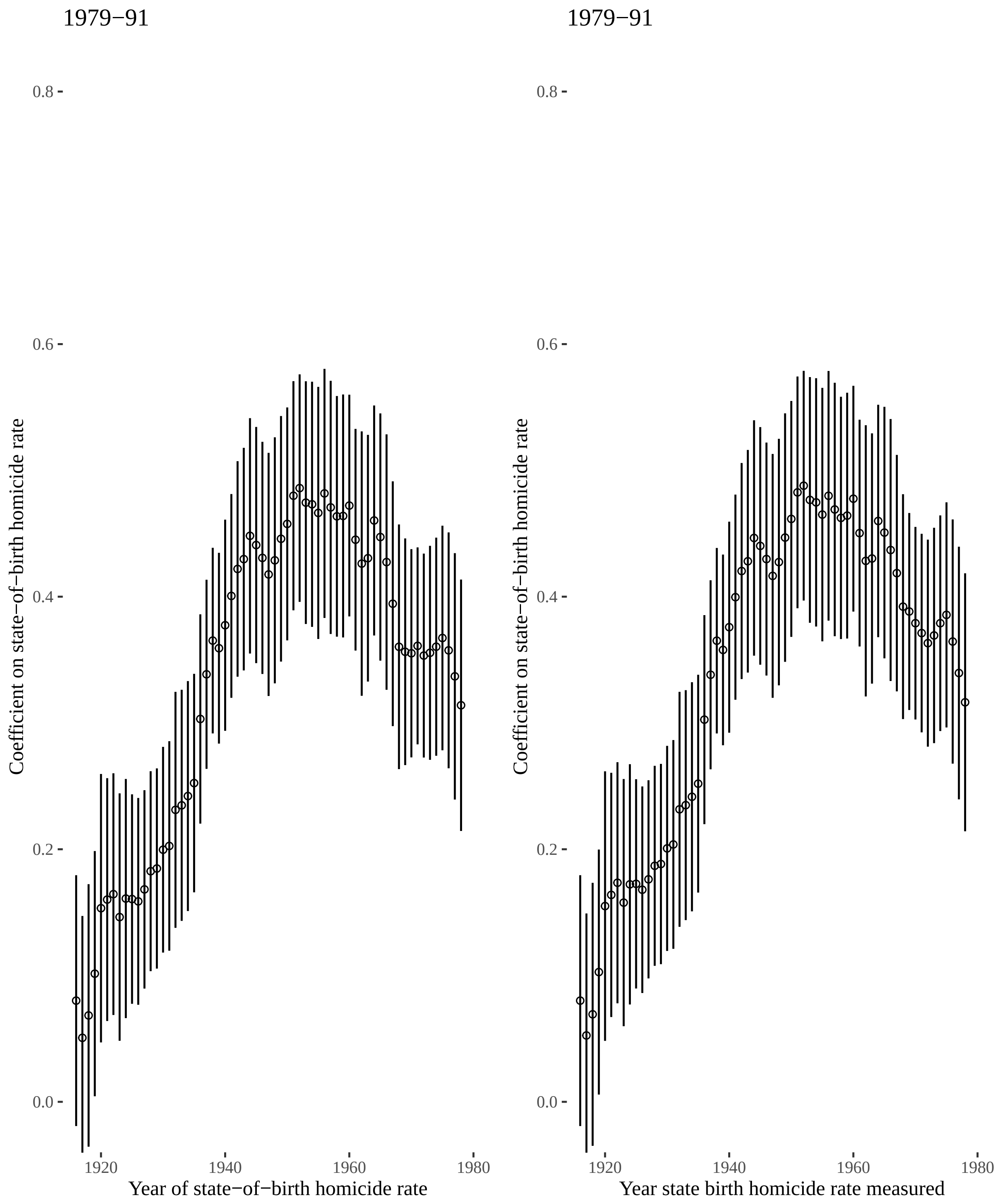

Figure S12: This figure builds on the previous figure by showing how the persistence estimates vary for 1979-91 excluding migrants born in New York state (left) and migrants born in New York or DC migrants (right). Please see the note to the previous figure for details. One explanation for why later state-of-birth homicides are more predictive is simply improvement in measurement. Although death certificate data is generally considered high quality, it may not have captured all homicides, especially in weakly-governed violent states, until the 1950s.

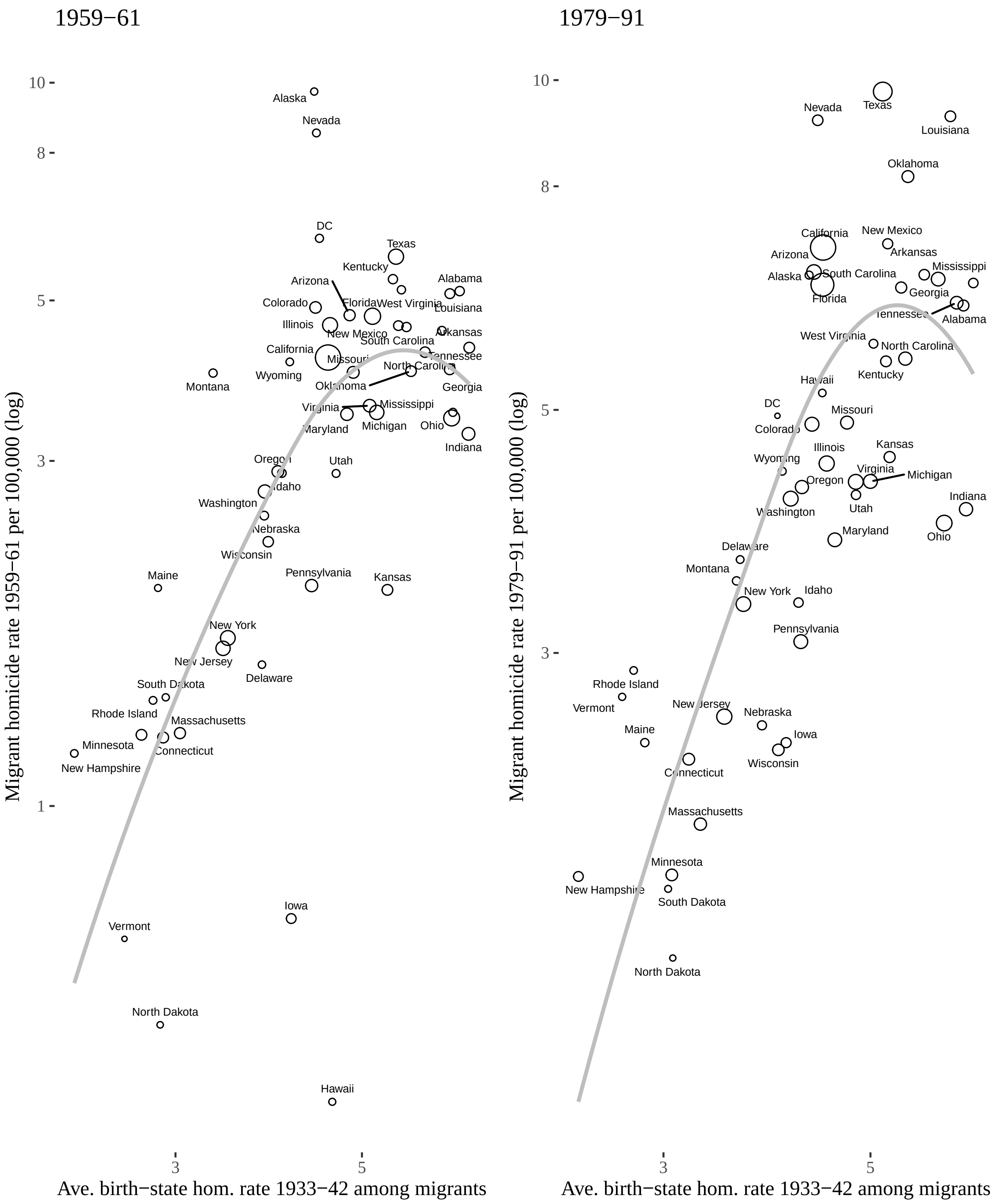

Figure S13: Average victimization rates in each state of residence in 1959-61 and 1979-91 by the average historical homicide rate of migrants each state received. The figure shows that Ohio and Indiana received migrants disproportionately from the most violent states and had some of the highest rates of homicide among those migrants. In the 1980s, however, those states appear to have lower rates of victimization for their migrants. Loess lines are weighted and circles are sized by white population.

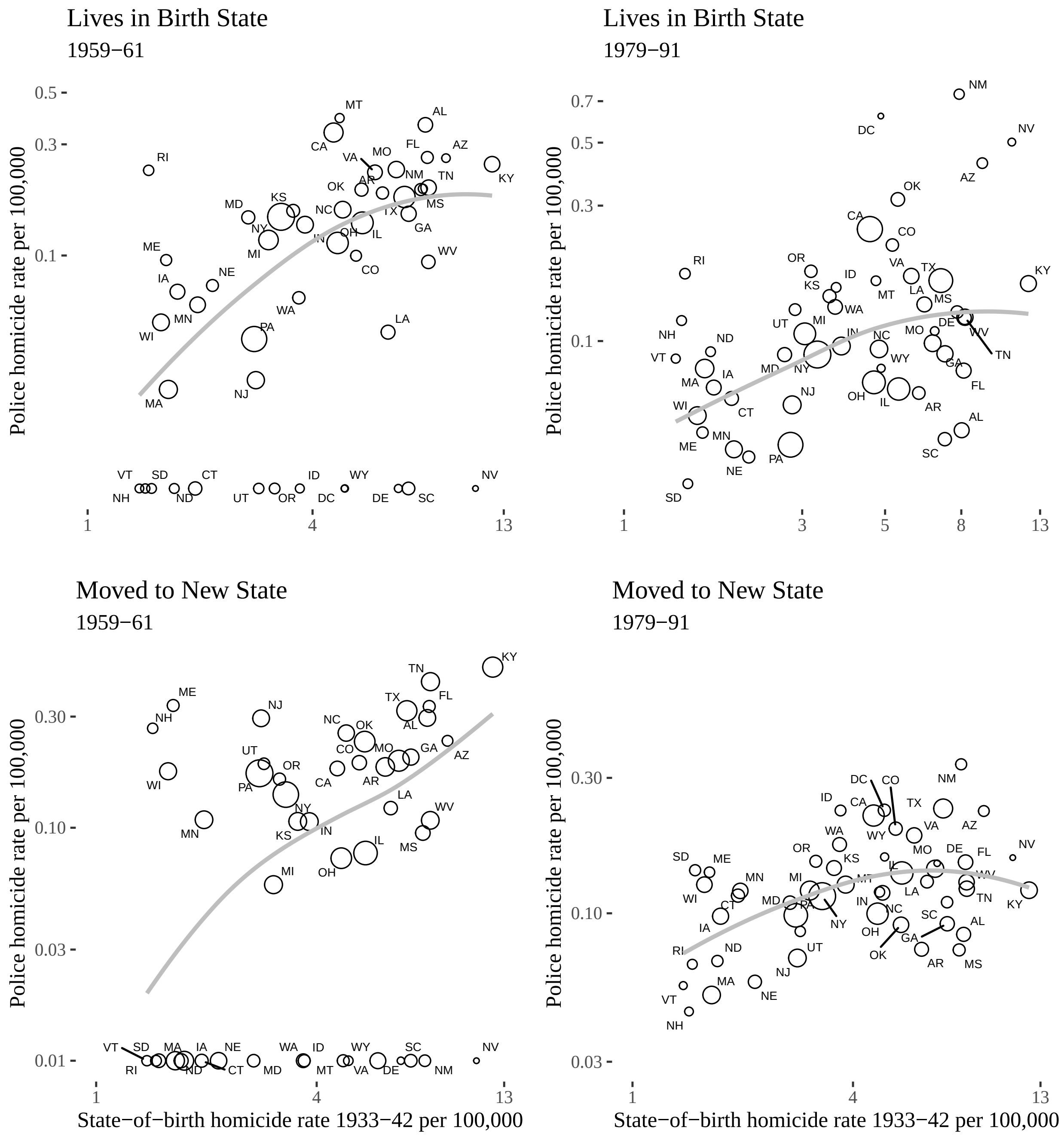

Figure S14: Police Homicide Victimization Rate 1959-61 and 1979-91 by Historical State-of-Birth Homicide Victimization Rate. Loess lines are weighted and circles are sized by white population. We add 0.01 to the police homicide rate in 1959-61 before logging because several states have no police homicides. The data capture 202 non-migrants and 117 migrant police homicides in 1959-61, and 1,085 non-migrant and 659 migrant police homicides in 1979-91. The bivariate OLS estimate for migrants in 1959-61 years is 1.78 (CI 95%: 0.33– 3.23), implying that a one-percent increase in the historical homicide rate corresponds with a 1.78-percent increase in the police homicide rate. Police violence against migrant groups from unsafe places may backfire, fostering distrust in local law enforcement institutions (Goldsmith 2005).

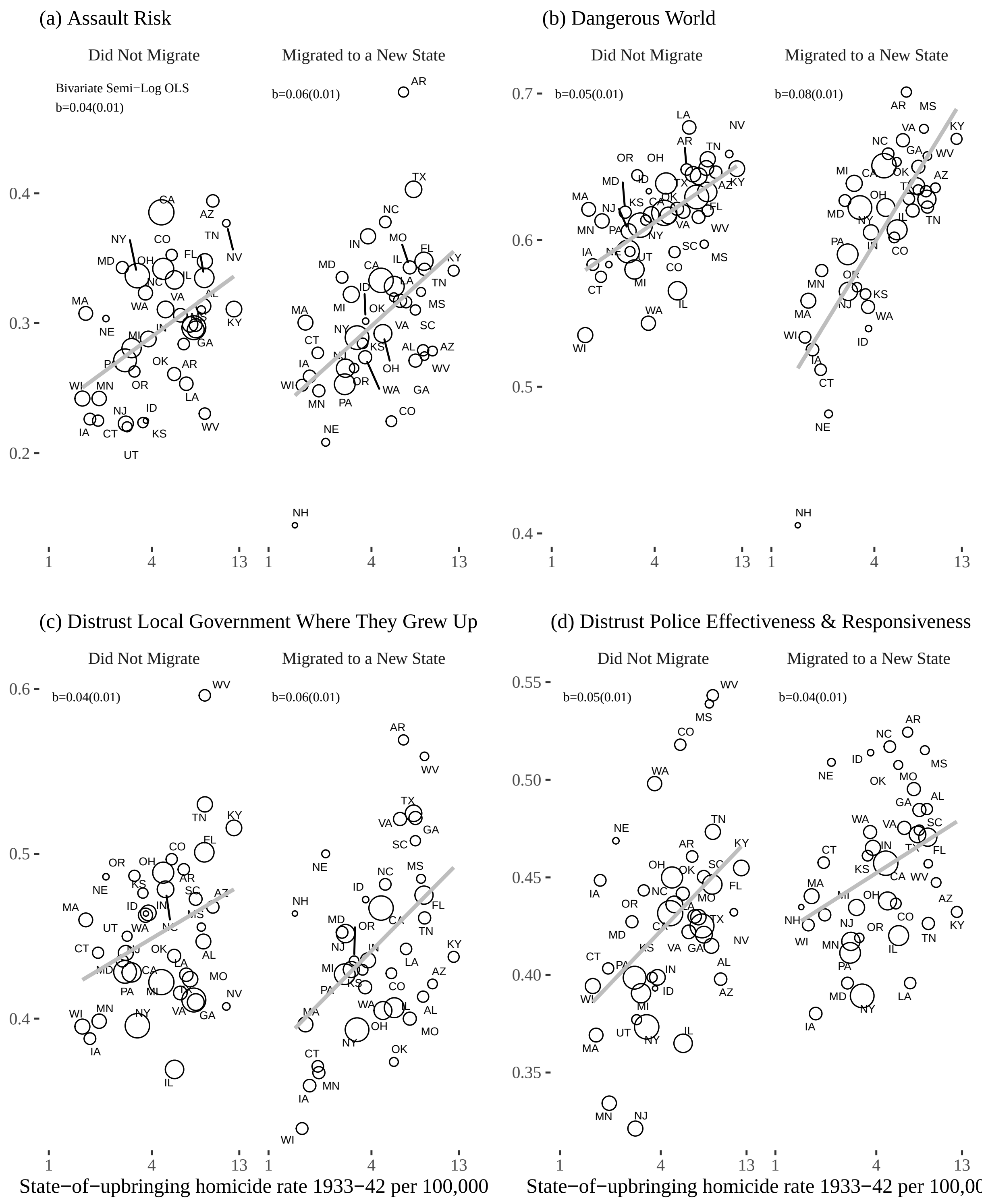

Figure S15: Selected Survey Measures by Historical State-of-Upbringing Homicide Victimization Rate for Whites by Migration Status. Linear best fit lines are weighted and circles are sized by the number of respondents. For the figure only, we exclude states with 20 respondents or fewer. Circles with fewer than twenty respondents are not shown. All respondents are non-Hispanic white. Note that Y-axis ranges vary for each figure. All dependent variables are scaled from 0 to 1.

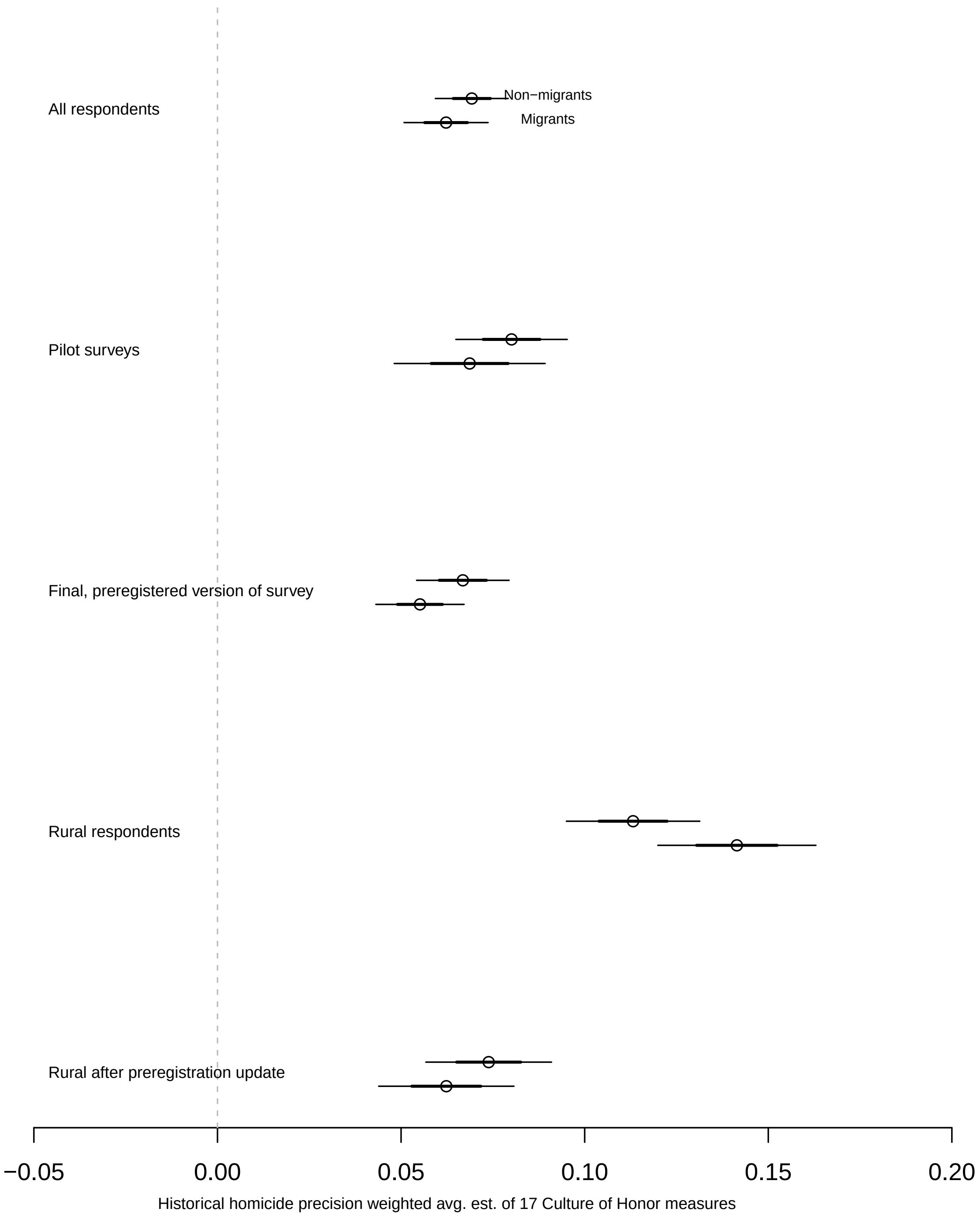

Figure S16: This figure shows the precision weighted average estimate for the effect of 17 Culture of Honor related measures for various samples. It starts by showing all respondents, then it shows only the estimates based on respondents in the pilot surveys, in the final preregistered version of the survey, in a sample of rural respondents, and in the sample of rural respondents after we updated our preregistration on September 11, 2023 (partway through the final version of the survey).

S3 Data Availability Statement

Data and code to replicate all results will be posted to the project's OSF page on publication with the exception of the last period we examine with the death registry data, 2000-17. For these years, the CDC restricts access to death certificate data, requires a data user agreement, and prohibits most redistribution. We are allowed to make highly aggregated data available for replication, but full replication of the 2000-17 findings will require a CDC agreement and specialized data protective services from one's institution. It's important to note that the findings in this last period simply replicate the findings in the two earlier periods we examined with death certificate data, and these earlier data are publicly available.

S4 Death Registry and Census Data

1959-61 death registry data are from NBER.[1] They include this note about the 1959-1967 data:

> The chief of the NCHS mortality branch has said that while the 1959-1967 files are generally good quality, they have not been rigorously verified. "Counts by selected causes and demographic groups seem to match up with VSUS, but because in some cases these files had to be reconstructed and pieced together from different sources-some were damaged or lost-we cannot at this time be certain as to their accuracy."

Any errors are unlikely to produce the main findings in the paper, especially since these findings also show up long after 1967. We use the 5% 1960 Census IPUMS file to provide the population counts and other census variables for these three years. The Census and mortality data have very low rates of missing data. State of residence, race, and sex are complete for all homicides, though this likely implies some amount of guessing on the part of coroners and funeral home directors. State of birth is missing in 1% of homicides, age in 0.25%, and marital status in 3%. The census data appear complete.

1979-91 Death registry data are from the Center for Disease Control.[2] The data contain few missing values: none for state of residence, under 2% for state of birth, and sex is complete, while race is missing in 1% of homicides, age in 0.05%, and marital status in 0.86%. We use the 5% 1980 and 1990 Census IPUMS files to provide the population counts and other census variables. In 1980 and 1990, variables are complete except for total family income with 2% missing (though for education census coding is ambiguous between no schooling and n/a). We aggregate and linearly interpolated census data from 1979 to 1991, with 1990 income deflated to 1980 beforehand. The aggregation and interpolation is to the state-of-birth, age group, sex, and state-of-residence level and to the state-of-birth-county level. Before interpolation, we impute missing aggregated education, income, and marital status for 1980 and 1990 in about 10% of state-of-birth-age-group-sex-state-of-residence data points and about 40% of

[1] Mortality Data - Vital Statistics NCHS Multiple Cause of Death Data https://www.nber.org/research/data/mortality-data-vital-statistics-nchs-multiple-cause-death-data

[2] https://www.cdc.gov/nchs/data_access/cmf.htm

state-of-birth-county-of-residence data points using other demographics, but these aggregates receive little weight in analyses because of very small populations. We never impute homicides or state of births.

2000-17 Death registry data are from the Center for Disease Control (CDC). The CDC restricts access to death certificate data, requires a data user agreement for access, and prohibits most data redistribution. We are allowed to make highly aggregated data available for replication, but full replication will require an extensive process with the CDC and specialized data protective services from one's institution. Data on homicide victims' sex and race are complete, although 0.7% of observations are missing data on Hispanic ethnicity. Age is missing from 0.1%, state of residence from 0.5%, state of birth from 11%, and marital status from 1.5% of observations. We match these observations to demographic data from the 5% sample of the 2000 census, 1-year American Community Survey datasets 2001-2006, the 2008 3-year ACS, and the 2012 and 2017 5-year ACSs. Variables from these datasets are complete except for total family income with 3.3% missing and education with 3.5% missing (unlike the 1980 and 1990 census data files, these later years disambiguate no schooling and n/a). Due to changes in the ACS's coding scheme for education compared to the earlier two periods and subsequent adjustments to our coding of education, descriptive estimates for education are slightly lower for this period compared to 1959-61 and 1979-91.

S5 Correlate Benchmarks

In the paper, we compare the correlates of historical state-of-birth homicide rates to the correlates of race and age on homicides in 1959–61. For the age association, we limit the model to white non-migrants and estimate how homicide rates decline with age, using the lower bound of each five-year age group as a linear predictor, with state-of-residence fixed effects. For the race effect, the model examines homicide rates among non-migrants and estimates the average difference between Black and white individuals, with age-group and state-of-residence fixed effects. For all models, we exclude Alaska and Hawaii, as we do throughout this paper because we lack historical homicide rates for these.

To compare migration to race and age, we calculate how much the homicide rate increases when moving from the least to the most violent state of birth among white migrants, using the baseline model in Table 1 (row 2). We then express that increase as a fraction of the estimated increase associated with being Black rather than white, and separately as a fraction of the decrease associated with aging from 20 to 80 among white non-migrants. These comparisons allow us to benchmark migration against two familiar sources of variation in homicide risk.

As we report in the paper, the increase in homicide rates for white migrants born in the most violent states

- Exceeded the entire decline in rates associated with aging from 20 to 80 by 141% and
- Amounted to 50% of the total gap between Black and white non-migrants.

The comparisons we report in the paper remain similar in other specifications.

- When we restrict the estimates from all three models to males, these are 140% and 53%, respectively.
- When we restrict the estimates from all three models to females, these are 99% and 39%, respectively.
- When we restrict the estimates for age and race to migrants, these are 158% and 43%, respectively.

S6 Survey

Due to space constraints, our discussion of the survey in the paper is brief. Here we provide a detailed discussion, including reporting Cronbach's alphas for all scales, discussing the results for each survey item, and discussing our preregistration and several minor deviations from it.

We conducted pilot surveys on Lucid Theorem and additional pilots and the final survey on Lucid Marketplace. Thanks to the Lucid staff, we successfully prevented respondents from participating in the survey more than once across all versions. We utilized Lucid Marketplace to exclusively survey non-Hispanic white respondents matching the age profile of migrants. While the Lucid Theorem pilots included Hispanics and nonwhites, we excluded them from our analysis. Pilot 1 of the survey ran from April 6 to April 18, 2022; 2 from April 30 to May 2, 2022; 3 from March 2 to March 4, 2023; 4 from March 15 to March 16, 2023; 5 from September 4 to September 6, 2023; the final survey ran from September 7 to September 19, 2023. We also conducted another pilot study, where several innovations failed to pan out, and so pregistered excluding it from the analysis.

In the pilots and in the full version, we drop respondents who fail to complete the survey and who fail an attention test late in the survey (preregistered). (To enter the survey, they must pass two simple attention checks.) In the pilots and in the full version, we also drop respondents who did not report their state of residence or a US state where they grew up (we didn't pre-specify these, but they are necessary since the point of the survey is to study migrants and non-migrants). We also drop a handful of respondents that Qualtrics security software labeled as duplicates (not pre-specified).

For the Lucid Marketplace respondents, we established quotas for our survey using the following categories: age and gender groups (18-29, 30-44, 45-64, 65 and over, each for female and male), geographic regions (Northeast, Midwest, South, West), household income levels (under $29k, $30k-59k, $60k and up), and education (less than college, college and above). We also applied a quota within the survey to keep the number of migrants and non-migrants similar. To ensure that this did not result in very different migrants and non-migrants, we combined it with age and gender quotas (e.g., 18 to 29-year-old female migrants, 18 to 29-year-old female non-migrants, etc.). Thus, we applied two sets of quotas, one to enter the survey and one within the survey to ensure that we were recruiting similar migrants and non-migrants. To set the quotas, we used the American Community Survey and selected quotas for non-migrants to match the demographics of migrants, who tend to be older.

Our survey is broadly representative of non-Hispanic white internal US migrants. As we discuss in the paper, we focus on this population for methodological reasons. We also do so because we aim to demonstrate that the patterns we document apply to non-minorities. Often, minority migrants from weak-institutional regions face criminalization for taking defense and justice into their own hands. We hope our findings will help people understand that these behaviors are not malicious. Convincing people is easier when whites exhibit these behaviors.

All survey instruments and replication data will be posted to the OSF replication page for this paper.

We define migrants in our survey data as those who indicate that they grew up in a different state than the one in which they currently reside. We use the state in which people say they grew up rather than the state in which they were born since, in cases where these locations differ, the state in which someone spends the majority of their childhood is more likely to impart beliefs and cultural norms.

Our analysis of the survey closely follows our analysis of homicide rates. We estimate individual-level models, regressing survey responses on the logged historical homicide rate in respondents' birth states, an indicator for migrant status, and the interaction between these variables. In the following, we present a more detailed discussion of the findings than we could include in the paper. In this discussion, we focus on the two pre-registered tables, which differ somewhat from Table 3 in the paper, though all produce similar substantive findings.

Table S30 presents the main findings. To ensure that migrants' self-selection into dangerous states does not drive the estimates, Table S31 shows the migrant estimates separately with state-of-residence fixed effects.[3] We preregistered these tables and the code to produce them, though see our note on several minor deviations from the preregistration below. In all models, we include gender and five-year age group fixed effects. We also cluster the standard errors at the level of the state where they grew up. To simplify the interpretation of the estimates, we rescale all variables to 0-1, including the logged historical homicide rate. When constructing multi-item scales, we take the simple average of the scale components using all available respondents (no listwise deletion).

It is worth emphasizing that we generally expect to find small coefficients. We hoped that these reverberations from long ago would be just strong enough to be still detected in noisy survey responses. Indeed, although we can still detect persistence in historical homicide rates in the 2000s, this persistence is much weaker than in the 1960s.

If the persistence of violence is a response to the belief that the world is a violent and dangerous place, we should see a relationship between these phenomena in survey responses. Tables S30 and S31 therefore begin by examining whether the historical homicide rate predicts witnessing violence growing up, e.g., "Did you ever see someone be hit, beat, kicked, roughed up, or deliberately hurt," using a three-item scale (Cronbach's alpha = 0.74). The estimates indicate that respondents from historically violent states witness more violence. Since all the measures are on a 0-1 scale, a shift from the least violent state for whites in the 1930s to the most violent state corresponds with

[3] As in the homicide victimization analysis, we cannot estimate these relationships with state of resident fixed effects for non-migrants because by definition all non-migrants live in the state where they grew up, so there is no within-state variation in historical homicide rates.

a 0.05 increase on the witnessing violence 0-1 measure. Table S31 shows that this relationship remains small but statistically significant among migrants within their states of residence. As in the analysis of the persistence of violent victimization, Table S30 includes a persistence column where we divide the migrant coefficient by the non-migrant coefficient multiplied by 100. In the analysis of homicides, this calculation captures the percent of persistence over time. In the survey analysis, it captures not persistence over time but carryover to migrants. We only calculate this statistic in rows where the absolute value of the coefficient for non-migrants is larger than 0.03 (a preregistered threshold). In this case, 67% of the relationship carries over.

In a similar vein, we next examine whether people who grow up in historically violent states are more likely to see the world as a dangerous place. We first use a three-item battery that asks about assault risk: the risk of being mugged, violently attacked, and having their home invaded by an armed burglar (Cronbach's alpha = 0.9). We also asked a two-item battery about how dangerous they perceive the world to be with items that include whether "there are many dangerous people in our society who will attack someone out of pure meanness" (Cronbach's alpha = 0.77). Figures S15(a) and (b) revealed that they do appear to assess a higher assault risk and to see the world as a more dangerous place, and this perception carries over to migrants. Figure S15's panels presented simple scatterplots aggregated the birth-state level. Table S31 shows that these relationships remains highly statistically significant among migrants within their states of residence. We also find that they remain unchanged when controlling for partisan identification.

Contrary to our expectations, the persistence of high homicide rates could alternatively be about disregard for laws in general. To explore this possibility, we asked a three-item legal cynicism scale (Karstedt and Farrall 2006; Sampson and Bartusch 1998), with items such as "sometimes you need to ignore the law and do what you want to." The estimates however show that, if anything, respondents from historically violent states believe more in the importance of following laws. Society tends to criminalize those involved in violence, assuming they disregard laws with impunity. At least according to what they say on surveys, however, this common perception isn't reflected in the data.

Next, Tables S30 and S31 examine whether persistence is about the perception of a lower living standards using a two-item scale (Cronbach's alpha = 0.64) with items about satisfaction with their current standard of living and about how it compares to their parents at the same age. The tables reveal essentially no relationship, providing another data point inconsistent with an economic account.

We expect that persistence emanates from peoples' responses to weak institutions in their states of origin. We asked: "How much trust and confidence did you have in local governments where you grew up when it comes to handling local problems?" We also asked the same question about local governments where they live now. Finally, we asked a three-item battery about how much they trust the police where they live now to prevent violence, catch people who committed burglaries, and arrive quickly during an emergency (Cronbach's alpha = 0.83). For most people, government institutions are remote abstractions they don't think about often, so we don't expect to find much. Nonetheless, migrants and non-migrants from states with high historical homicide rates are slightly more likely to distrust institutions, especially those where they grew up (Figures S15(c) and (d)). Table S30 presented these estimates individually and with a precision-weighted average estimate, which has enough power to detect statistically significant effects. These associations, however,

weaken among migrants when we only examine then within states of residence in Table S31.

Next, we turn to potential attitudinal, behavioral, personality, and value-based responses to these weak institutions. When people face absent or hostile governments, they tend to rely more on families when facing danger. We asked a two-item question about whether people trusted family or trusted police more when someone in their family was being attacked or someone was stealing from them (Cronbach's alpha = 0.8). As expected, we find that people are more likely to rely on families than on the police when they grew up in historically violent states, a pattern that persists among migrants in their new states, including when we only examine this relationship within states of residence. Figure 2(a) presented a scatterplot of this relationship.

Research suggests weak institutions also lead to less trust in other people, so we asked whether "most people can be trusted" or "you need to be very careful in dealing with people." We asked about trust separately for where respondents grew up and where they live now. The estimates again show that people who grew up with historically high homicide rates trust people less where they grew up, though less clearly so for where they now live.

Next, we examine a behavioral response to historically high homicide rates: whether people own guns for protection rather than for other reasons. In the survey, we asked whether they or somebody in their family owned a gun. If they answered 'yes,' we asked whether it was mainly for protection, at least somewhat for protection, or not for protection. We create indicator variables for each response, with the other responses set to zero. Figure 2(b) showed, as we would expect, that people from historically violent states report owning guns for protection at much higher levels. Tables S30 and S31 show that this pattern holds with our controls and among migrants within their states of residence. Compared to people from the least violent states, non-migrants from the most violent are about 28 percentage points more likely to say they own a gun for protection. Among migrants, this difference is about 16 percentage points. By contrast, respondents from the most violent states are not more likely to say that they own a gun partly for protection or not for protection. As these estimates imply, the own-for-protection relationship is larger than the simple own-a-gun relationship.

As we noted, research has found that individuals in weak-institutional settings respond aggressively to threats. If they back down instead, they will appear weak and therefore vulnerable to further victimization. To explore whether people from historically violent states feel the need to respond aggressively, we asked respondents about three scenarios. In each scenario, we described a person who faces aggression. In the Kevin scenario, a man pours beer on Kevin's head at a bar. In the Emma scenario, a girl scratches Emma repeatedly on their school bus. In the Doug scenario, a man cuts in line at the movies and threatens Doug when he objects. The individual then reacts aggressively, punching, slapping, or shoving, in the three scenarios respectively. We asked respondents whether they would respond aggressively, whether their friends where they grew up would do so, and whether the typical male or female (depending on the scenario) would do so, using a seven-point scale from "extremely unlikely" to "extremely likely." We asked about friends and typical males and females to assess how much respondents expect aggressiveness from the people around them and also in case respondents are reluctant to agree to aggression themselves but would admit aggression by those around them. It is important to reemphasize that we see these reactions as fundamentally defensive, part of a deterrence strategy, as they would say in international relations. Without institutions to protect them, people feel like they must respond

forcefully to threats. Tables S30 and S31 show the estimates, grouping them in various ways. It first shows estimates for scales created by averaging respondents' answers to the three scenarios by how the respondent would respond themselves (Cronbach's alpha = 0.81), how their friends would respond (Cronbach's alpha = 0.85), and how typical males or females where they grew up would respond (Cronbach's alpha = 0.87). Figure 2(c) showed the scatterplot for this last measure. Then we show the answers to these three questions averaged separately for the Kevin, Emma, and Doug scenarios (Cronbach's alphas = 0.86, 0.91, and 0.87, respectively). The estimates show that respondents from historically violent states do indeed react more forcefully themselves and expect their friends and typical males or females to also do so. Moreover, these patterns persist among migrants, including when we only examine the relationship within states of residence. About 80% of the association among non-migrants remains among migrants when we look at the precision weighted average.

To further test our interpretation that these aggressive responses are fundamentally defensive, we asked in the Kevin and Doug scenarios how respondents thought Kevin and Doug would feel if they walked away instead of responding aggressively. We asked whether he would "look weak in front of his friends" and "feel like he wasn't a real man." We take the average of these for each of the two scenarios (Cronbach's alphas = 0.69 for "look weak" and 0.71 for "a real man"). As expected, respondents from historically violent states are slightly more likely to respond in the affirmative to all three questions, a pattern that again persists for migrants.

The question about feeling like "a real man" captures a value we might expect people to hold in weak institutional settings. When the government is either absent or hostile, people may see strength and aggressiveness as central to their identities, as they keep them safe by deterring victimization. To explore this possibility, we included three items adapted from the Honor Ideology in Manhood Scale (Barnes, Brown, and Osterman 2012). We asked how important were the following to being a "real man" where you grew up: "never backs down from a fight," "isn't afraid to act with physical aggression toward another man who steals from him," and "doesn't take any crap from anybody." Using a scale of these three items (Cronbach's alpha = 0.89), Figure 2(d) and Tables S30 and S31 show that respondents from historically violent states say these are more important to being a "real man." These findings may help us understand the origins and persistence of "toxic masculinity" as an adaptation to weak institutions.

Similarly, scholars have noted the importance of what might be described as "hotheadedness" to safety in weak institutional settings, as a reputation for having a temper may protect people from victimization. In *Albion's Seed*, for example, Fischer reports that parents encouraged violent emotional responses in male children in the antebellum South, as individuals with such reputations may face less victimization (Fischer 1989). We therefore asked how well the following statements describe respondents: "Some of my friends think I am a hothead," "I have trouble controlling my temper," and "I am an even-tempered person." We adapted these from the Buss-Perry Aggression questionnaire (Bryant and Smith 2001). It is important to emphasize that we see this personality trait as fundamentally defensive. We also emphasize that we don't expect people to think through the importance of deterrence strategies in weak institutional settings. Instead, we suspect that cultural learning may lead people to adopt a response to the dangerous circumstances they face. Using a scale of these three items with the last one reverse coded (Cronbach's alpha = 0.81), respondents from historically violent states report being slightly more hotheaded, a pattern that

may again persist among migrants, but in a diminished form.

Finally, we asked a question about whether both parents raised respondents to crudely capture social disorganization (not pre-registered). We do find a relationship between historical homicide rates and this measure: respondents from the most violent states are about 10 percentage points less likely to be raised by both parents (see Table S30 for all of these findings).

As we note in the article, we find larger associations for rural respondents. We don't find larger ones for less educated respondents.

In the text, we report that results remain similar when we control for party identification. In the Lucid Theorem surveys, we have the standard American National Elections Study, seven-point party identification question. We add this to the models as a fixed effect for each of those seven points. Rescaling all measures to 0-1, the precision-weighted average estimated effect of historical homicide rate across the available culture-of-honor measures, controlling for gender, five-year age-group fixed effects, and fixed effects for seven-point party identification, is 0.093 (95% CI 0.074, 0.117) for migrants and 0.058 (95% CI 0.034, 0.082) for nonmigrants. This is similar to what we find without controlling for party identification, as we describe in the next section and show in Figure S16.

S7 Survey Analysis Preregistration

We preregistered the plan for analysis, in the form of two Tables, on September 7, after the pilots but before running the final version of the survey.[4]

- Tables S32 and S33 present these tables for only the pilot surveys.
- Tables S34 and S35 do so for the preregistered final survey.

We posted a revised version of our registration on September 11, midway through fielding the final survey, to specify two analyses not in the original plan.

- We accidentally omitted a dependent variable: belief in a dangerous world.
- We had intended to but forgot to specify that effect sizes should be larger among individuals who grew up in rural areas. We operationalized rural in the updated preregistration as scoring in the top two points of an index composed of our two questions related to ruralness: whether they grew up in a rural area and whether they currently reside in a rural area. The updated registration stated that we will present the results separately for respondents who score at the top of this index, i.e., the top two points. Table S19 shows the estimates for each item for rural respondents. Table S20 shows them for only those interviewed after the preregistration update. Figure S16 shows precision weighted averages for rural respondents before and after the preregistration update, which reveals a lack of precision for migrants postupdate but not non-migrants. Additionally, we said we would examine the interaction between a dummy

[4]https://osf.io/pr97f/?view_only=befe13d162f94af5a7533624977ae629

variable indicating whether respondents fall into the top two points and the logged historical homicide rate, using the same specifications we use throughout the analysis in the tables below.

To report these interactions, we estimate each one in a separate equation just for migrants and then pool them with a precision weighted average. Rescaling all measures to 0-1, the precision-weighted average estimated effect of historical homicide rate across all culture-of-honor measures, controlling for gender and five-year age group fixed effects, is 0.047 (95% CI 0.035, 0.059) for non-rural respondents and the interaction between the historical homicide rate and this rural indicator is 0.129 (95% CI 0.128, 0.13). These estimates are consistent with Table S19, which shows the estimates for each item for rural respondents for non-migrants and migrants (though the specifications differ slightly).

We deviate from the pregregistration in Tables S30 and S31 in the following ways.

- We include a battery called assault risk which we added in our last pilot survey but forgot to preregister. The estimates are sufficiently precise in Tables S30 and S31 that would have had to be cherry picking from many items for this to be a false positive (and we disclose all survey items).
- We include a indicator variable for being raised by both parents. The relationship between historical homicide rate and not being raised by both parents was sufficiently strong that we felt it important to report. Of course it may or may not be an important channel through which persistence occurs.

The main analysis we present in the paper, Table 3, deviates from our main pre-registered table (Table S30). Based on a thoughtful request from a reviewer, it includes state of residence fixed effects for migrants and the number of years in school. The substantive results change little with these additions, and it's worth noting that our second pre-registered table, Table S31, did include fixed effects for residents.

S8 Lessons from Pilot Surveys

We conducted several pilot surveys with various questions but eliminated them before the final version because they did not correlate with historical homicide rates for non-migrants or migrants. These include:

1. Two question sets about expectations of violence in the respondents' childhood environments, removed early from the pilot surveys. One battery inquired if people worried about being struck first during an argument, felt the need to act before being attacked, or feared appearing weak if they backed down. Another gauged surprise at the occurrence of violence in specific places or situations, like bars or during disputes. We excluded these because neither performed as well as the Assault Risk and Dangerous World batteries included in the final survey.

2. A set based on the culture of honor concept asking about teachings from friends and family, including beliefs about respect, intimidation, and the need to appear tough. This set was less effective than the Honor Ideology and Manhood battery in the final version.

3. A set about efforts to avoid escalating arguments, insulting individuals, theft, or home invasions. These items underperformed compared to the Assault Risk and Dangerous World batteries.

4. A version of the threatening scenarios asking respondents how they would react (strike or not). This approach seemed less effective than having the aggrieved individual striking back in the scenarios and asking respondents whether they would do the same.

5. The Revenge Planning Subscale from the Displaced Aggression Battery (Denson, Pedersen, & Miller 2006). While there were indications of a relationship with historical homicide rates, it was too weak for the final survey. Instead, our takeaway from these questions are that young people and especially younger men think a great deal about getting back at people who have harmed them. Those thoughts do not seem to vary with the historical homicide rate. These questions don't explicitly ask about violent revenge. So our hunch is that young men from historically safe states are much more likely to be thinking about non-violent revenge.

6. A set inspired by a Chicago schools study, focusing on qualities sought in friends, such as avoiding fights and dependability in a fight. These did not correlate with historical homicide rates.

7. A set about disagreeableness, with statements like being critical and quarrelsome, showing no relationship with historical homicide rates.

8. Items adapted from the street-code battery (Stewart & Simons, and Piquero 2012), such as the counterproductiveness of arguing or fighting, the respect garnered by toughness, and the importance of not being intimidated. These were less effective than our three scenarios or the Honor Ideology and Manhood battery.

9. A police-battery about the effectiveness of police where respondents grew up. Interestingly, historical homicide rates correlated more with perceptions of police effectiveness where they live now, suggesting respondents might struggle to accurately recall distant governmental institutions.

10. A pilot randomizing most survey questions resulted in lower correlations with historical homicide rates. We speculate that the order of questions in our survey better frames respondents' mindsets about their youth. This pilot was excluded in our pre-registration. For replicating our results, surveys should stick closely to the order we settled on (see the survey in the next section).

S9 Survey Questions

The survey questions below are from the final version of the survey. In the pilot studies, the language may differ slightly. We will post the survey questions of all pilot version to our OSF project page.

Attention Checks

Hello and welcome!

We need to make sure that everyone who is taking our survey is paying close attention.

Please read carefully and answer the following questions.

Please click BOTH "I understand" AND "I don't understand."

- I understand (1)
- I don't understand (2)

In order to proceed, we request that you complete this attention check. Please select "I am a robot" below.

- I am a robot (1)
- I am not a robot (2)

Legal cynicism

Given where you grew up, how much do you agree or disagree with the following ...

- Sometimes you need to ignore the law and do what you want to
- People who obey rules often disadvantage themselves

Given where you grew up, how much do you agree or disagree with the following ...

- To make money, there are no right and wrong ways, only easy ways and hard ways

Answers: Strongly disagree (1) Somewhat disagree (2) Neither agree nor disagree (3) Somewhat agree (4) Strongly agree (5)

Hotheadness

Given where you grew up, how well do the following statements describe you?

- Some of my friends think I am a hothead
- I have trouble controlling my temper

Answers: Very unlike me (1) Somewhat unlike me (2) A bit unlike me (3) A bit like me (4) Somewhat like me (5) Very like me (6)

(New page)

Given where you grew up, how well does the following statement describe you?

- I am an even-tempered person

Answers: Very unlike me (1) Somewhat unlike me (2) A bit unlike me (3) A bit like me (4) Somewhat like me (5) Very like me (6)

Witness violence

Did you ever see someone be hit, beat, kicked, roughed up, or deliberately hurt . . .

- at your high school? (1)

- at your junior high? (2)

Answers: Never (1) Rarely (2) Sometimes (3) Frequently (4)

When you were growing up, did you ever see adult males fighting (striking, punching, hitting, stabbing, shooting, etc.)?

Answers: Never (1) Rarely (2) Sometimes (3) Frequently (4)

Assault risk

Given where you grew up, what do you estimate is the likelihood the following will happen in your lifetime (in your future)?

- Likelihood you will be mugged
- Likelihood you will be violently attacked
- Likelihood your home will be invaded by an armed burglar

Answers: Highly unlikely (1) Somewhat unlikely (2) Neither likely nor unlikely (3) Somewhat likely (4) Highly likely (5)

Kevin Scenario

We'd like to ask about the following scenario: Kevin is at a bar one evening. He is sitting at a table having a beer and cheering on his team as it wins the game. A fan of the other team walks by and pours beer on Kevin's head and doesn't apologize. Kevin gets up and punches him. How likely is punching (like Kevin did) in this scenario for . . .

- You?
- One of your friends where you grew up?
- A typical male in the neighborhood where you grew up?

Answers: Extremely unlikely (1) Moderately unlikely (2) Slightly unlikely (3) Neither likely nor unlikely (4) Slightly likely (5) Moderately likely (6) Extremely likely (7)

If Kevin walked away instead, would he . . .

- Feel like he wasn't a real man
- Look weak in front of his friends

Answers: 0-10 slider with and labeled Strongly disagree and Strongly agree

---

Doug Scenario

We'd like to ask about the following scenario:

Doug is standing in line for a movie with his friend. A guy cuts into the line right in front of Doug. When Doug politely says something, the guy starts insulting and threatening Doug.

After taking it for several minutes, Doug shoves him. How likely is shoving (like Doug did) in this scenario for . . .

- You?
- One of your friends where you grew up?
- A typical male in the neighborhood where you grew up?

Answers: Extremely unlikely (1) Moderately unlikely (2) Slightly unlikely (3) Neither likely nor unlikely (4) Slightly likely (5) Moderately likely (6) Extremely likely (7)

Emma scenario

One final scenario:

On the last two school bus trips home, a girl deliberately scratched Emma hard with her nails.

Emma decides she has to do something if it happens again.

Sure enough, on the next trip, the girl again attacks Emma for no reason.

Emma slaps her in the face.

- You?
- One of your friends where you grew up?
- A typical female in the neighborhood where you grew up?

How likely is slapping her (like Emma did) in this scenario for . . .

Answers: Extremely unlikely (1) Moderately unlikely (2) Slightly unlikely (3) Neither likely nor unlikely (4) Slightly likely (5) Moderately likely (6) Extremely likely (7)

Honor ideology for manhood How important are the following to being a "real man" where you grew up?

- Never backs down from a fight (2)
- Isn't afraid to act with physical aggression toward another man who steals from him (3)
- Doesn't take any crap from anybody (4)

Answers: Not at all important (1) | Slightly important (2) | Moderately important (3) | Very important (4) | Extremely important (5)

Belief in a dangerous world

Given where you grew up, how much do you agree or disagree with the following?

Answers: Strongly disagree (1) | Somewhat disagree (2) | Neither agree nor disagree (3) | Somewhat agree (4) | Strongly agree (5)

- Any day now chaos and anarchy could erupt around us. (1)

- There are many dangerous people in our society who will attack someone out of pure meanness. (2) (New page)

Given where you grew up, how much do you agree or disagree with the following?

Answers: Strongly disagree (1) | Somewhat disagree (2) | Neither agree nor disagree (3) | Somewhat agree (4) | Strongly agree (5)

- The social world we live in is safe. (1) (Reverse coded)

Social trust

Generally speaking, where you grew up, would you say that most people can be trusted or that you need to be very careful in dealing with people?

Answer on a 0-10 slider With the endpoints labeled "Need to be very careful" and "Most people can be trusted."

How much trust and confidence did you have in the local government where you grew up when it comes to handling local problems?

Answer: None, Not very much, A fair amount, A great deal

Trust family or police

Imagine adults you knew growing up got into a dispute so heated that it could turn violent.How likely is it that they would call the police to help resolve it?

Answers: Extremely unlikely, Somewhat unlikely, Neither likely nor unlikely, Somewhat likely, Extremely likely

Would you mostly trust the police or would you mostly trust family and friends to help if . . .

- Someone was attacking me
- Someone was stealing from me

Answers: Completely trust police to handle it (1) | Mostly trust police to handle it (2) | Trust police, family and friends equally (3) | Mostly trust family and friends to handle it (4) | Completely trust family and friends to handle it (5)

Trust measures

Generally speaking, where you live now, would you say that most people can be trusted or that you need to be very careful in dealing with people?

Need to be very careful - Most people can be trusted

0 1 2 3 4 5 6 7 8 9 10

Now, how much trust and confidence do you have in the local government where you live now when it comes to handling local problems?

- None (1)
- Not very much (2)
- A fair amount (3)
- A great deal (4)

Trust in police response

How successful do you think the police are where you live now at preventing violent crimes?

How successful do you think the police are where you live now at catching people who committed burglaries?

- Very successful (1)
- Somewhat successful (2)
- Moderately successful (3)
- A little successful (4)
- Not at all successful (5)

How quickly would the police arrive where you live now when called to an emergency?

- Very quickly (1)
- Fairly quickly (2)
- Moderately quickly (3)

- Fairly slowly (4)
- Very slowly (5)

Standard of living

How satisfied are you with your standard of living?

- Extremely dissatisfied (1)
- Somewhat dissatisfied (2)
- Neither satisfied nor dissatisfied (3)
- Somewhat satisfied (4)
- Extremely satisfied (5)

Compared to your parents at your age, do you think your own standard of living is . . .

- Much better (1)
- Somewhat better (2)
- About the same (3)
- Somewhat worse (4)
- Much worse (5)

Parent birth

In which state was your mother born?

Who was the last question about?

- You (1)
- Your mother (2)
- Your father (3)
- Your pet (4)

In which state was your father born?

Urban v rural

Would you say you live in . . .

- A large city (1)

- Suburb near a large city (2)

- Small city or town (3)

- A rural area (4)

Would you say you grew up in . . .

- A large city (1)

- Suburb near a large city (2)

- Small city or town (3)

City What city/town and state did you go to high school in? E.g., Springfield, Illinois.

Gun ownership

Do you or anyone in your family own a gun?

- Yes (1)
- No (2)

[If yes] Is the gun . . .

- Mainly for protection (1)
- At least somewhat for protection (2)
- Not for protection (3)

Parents

Were you primarily raised by . . .

- Both parents (1)
- Mother (2)
- Father (3)
- Grandparent or grandparents (4)
- Other guardian (5)

S10 SI Citations

Barnes, Collin D., Ryan P. Brown, and Lindsey L. Osterman. 2012. “Don’t Tread on Me: Masculine Honor Ideology in the US and Militant Responses to Terrorism.” *Personality and Social Psychology Bulletin* 38 (8): 1018–29.

Bryant, Fred B., and Bruce D. Smith. 2001. “Refining the Architecture of Aggression: A Measurement Model for the Buss–Perry Aggression Questionnaire.” *Journal of Research in Personality* 35 (2): 138–67.

Denson, Thomas F., William C. Pedersen, and Norman Miller. 2006. “The Displaced Aggression Questionnaire.” *Journal of Personality and Social Psychology* 90 (6): 1032.

Fischer, David Hackett. 1989. *Albion’s Seed: Four British Folkways in America.* Oxford, England: Oxford University Press.

Goldsmith, Andrew. 2005. “Police Reform and the Problem of Trust.” *Theoretical Criminology* 9 (4): 443–70.

Karstedt, Susanne, and Stephen Farrall. 2006. “The Moral Economy of Everyday Crime: Markets, Consumers and Citizens.” *The British Journal of Criminology* 46 (6): 1011–36.

Ruggles, Steven, Sarah Flood, Daniel Backman, Annie Chen, Matthew Sobek, Grace Cooper, Stephanie Richards, Renae Rogers, and Megan Schouweiler. 2023. “IPUMS USA.” Minneapolis, MN.

Sampson, Robert J., and Dawn Jeglum Bartusch. 1998. “Legal Cynicism and (Subcultural?) Tolerance of Deviance: The Neighborhood Context of Racial Differences.” *Law & Society Review* 32 (4): 777–804.

Stewart, Eric A., and Ronald L. Simons. 2010. “Race, Code of the Street, and Violent Delinquency: A Multilevel Investigation of Neighborhood Street Culture and Individual Norms of Violence.” *Criminology* 48 (2): 569–605.
